# Comparing Neutral (Monometallic) and Anionic (Bimetallic) Aluminum Complexes in Hydroboration Catalysis: Influences of Lithium Cooperation and Ligand Set

**DOI:** 10.1002/anie.201806168

**Published:** 2018-07-09

**Authors:** Victoria A. Pollard, M. Ángeles Fuentes, Alan R. Kennedy, Ross McLellan, Robert E. Mulvey

**Affiliations:** ^1^ WestCHEM Department of Pure and Applied Chemistry University of Strathclyde Glasgow G1 1XL UK

**Keywords:** aluminum, bimetallic synergy, homogeneous catalysis, hydroboration, lithium

## Abstract

Bimetallic lithium aluminates and neutral aluminum counterparts are compared as catalysts in hydroboration reactions with aldehydes, ketones, imines and alkynes. Possessing Li–Al cooperativity, ate catalysts are found to be generally superior. Catalytic activity is also influenced by the ligand set, alkyl and/or amido. Devoid of an Al−H bond, *i*Bu_2_Al(TMP) operates as a masked hydride reducing benzophenone through a β‐Η transfer process. This catalyst library therefore provides an entry point into the future design of Al catalysts targeting substrate specific transformations.

The synthetic value of main‐group metal complexes aside from the highly reactive and versatile organolithium and organomagnesium reagents have, from a historical perspective, been overshadowed by the illustrious reputation of transition‐metal (notably precious metals) and lanthanide‐metal counterparts especially in catalysis.^1^ To a large extent main‐group research has been driven by fundamental curiosity and the understanding of the nature of chemical bonding and structure. A step‐change occurred when it was realised that such main‐group‐metal species can act in homogeneous catalytic roles, previously the exclusive province of transition‐metal and lanthanide complexes. Emulating the high reactivity, selectivity and versatility of the often toxic and scarce precious metal complexes is a tantalising challenge that needs addressing. In this regard, the pioneering work of Harder, Hill, Jones, Okuda, Power, Roesky, Wright among others, are expanding the vistas of main‐group complexes in homogeneous catalysis.^1^, ^2^ Since aluminum is the most abundant metal in the earth's crust, and also benefits from low toxicity, harnessing its reactivity is given high prominence in this main group uprising with longer term sustainability being a key issue. Thus, recently aluminum complexes have made significant strides forward in important stoichiometric and catalytic transformations.^3^ For example, they are utilised in C−C cross coupling chemistries, and in deprotonative metalation.^4^ Catalytic hydroelementation reactions have also witnessed impressive progress in the past few years. Roesky and co‐workers demonstrated that a β‐diketiminato stabilised aluminium hydride complex is an excellent catalyst for hydroboration of alkynes and carbonyl groups.^5^ More recently, Cowley, Thomas and Bismuto revealed that DIBAL(H), and Et_3_Al⋅DABCO can catalyse hydroboration of alkynes.^6^ Our group's interests lie in exploiting the synergistic reactivity imparted by two distinct metal centres^7^, ^8^ installed within a bimetallic complex. In this regard we introduced ate complexes (Figure 1), detailing that heteroleptic lithium diamido‐dihydridoaluminates and lithium monoamido‐monohydrido‐dialkylaluminates implicate that the alkali metal influences the ensuing “aluminum reactivity” in the hydroboration of aldehydes, ketones and terminal alkynes.^8^

**Figure 1:**
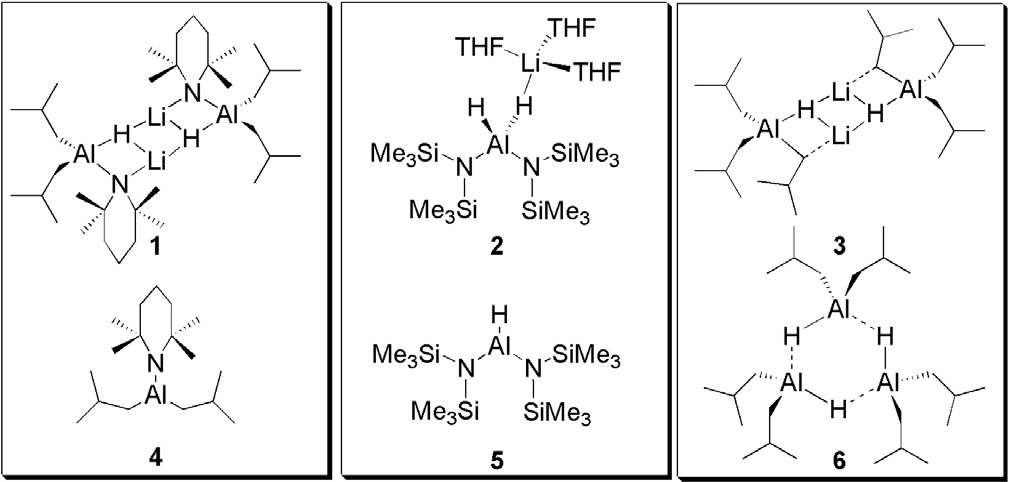
Al complexes **1**–**6** assessed in this study: ates **1**–**3**; neutral **4**–**6**.

Further, the catalytic chemistry of LiAlH_4_ has recently been explored by Cowley, Thomas and Bismuto in the challenging hydroboration of alkenes, however the role of the alkali metal was not elaborated.^9^ Thus, the current state of the field dictates that a systematic analysis of the secondary metal cooperative effects and various ligand factors that contribute to efficient hydroboration, is required in order to establish empirical rules for a posteriori design of future catalysts.

Hydroboration of unsaturated substrates under aluminum catalysis is gaining a foothold in the literature, and a variety of neutral aluminum complexes are displaying excellent potential in this role.2a, 3, 10 Previously, we reported that bimetallic lithium [*i*Bu_2_AlTMP(H)Li]_2_ (**1**) and [(HMDS)_2_AlH(μ‐H)Li⋅3 THF)] (**2**) are both efficient bimetallic (pre)catalysts in the hydroboration of aldehydes and ketones.^8^ However, any synthetic advantages/disadvantages of using ate complexes are yet to be fully uncovered, despite their potential. Thus, here, for the first time ate complexes are compared with their neutral aluminum counterparts to fully quantify their value in synthesis, and to glean understanding of their mode of action. Moreover, the complexes chosen vary in their ligand constitution, that is, alkyl versus amido constituents, providing further comparison. Mechanistically a frequently postulated two‐step reaction pathway is: 1) insertion of an unsaturated substrate into an Al−H bond; 2) σ‐bond metathesis with a borane, regenerating an active species and liberating product (Scheme 1).

**Scheme 1:**
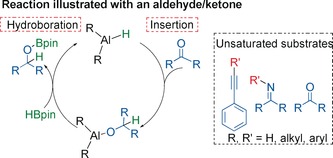
Postulated insertion mechanism in Al‐catalysed hydroboration.

Catalytic activities were screened with aldehydes, ketones, imines and alkynes, providing reaction scope to determine key divergences in catalyst reactivity. We previously reported **1** and **2** in catalytic hydroboration and these are compared with the neutral analogues **4** and **5**, which differ by formal removal of LiH.^8^ We prepared new complex **3**, an all alkyl variant of **1** (established via NMR characterisation, including DOSY) by a simple co‐complexation procedure (see Supporting Information). Compound **3** can be considered an ate version of DIBAL(H), **6**. Our results from comparative studies (reaction conditions are identical between different catalysts) are summarized in Table 1. Complexes **1**–**6** (5 mol %) were all tested in hydroboration reactions of benzophenone with pinacolborane (HBpin) at room temperature in J. Young's tubes in C_6_D_6_. Each bimetallic complex exhibits superior activity to its monometallic counterpart, affording quantitative conversion after 30 mins, apart from **4**, which is 94 % complete after 30 mins. This is surprising since **4** does not possess an Al−H bond. Rationalising that an Al−H bond must form in situ during the catalysis we performed a stoichiometric reaction between **4** and benzophenone in hexane and C_6_D_6_, where clear, facile quantitative reaction occurs rapidly at room temperature (isobutene, the coproduct of β‐hydride elimination, is seen in the ^1^H NMR spectra). X‐ray diffraction studies of colourless crystals grown from the hexane solution revealed formation of [(TMP){Ph_2_(H)CO}Al{μ‐OC(H)Ph_2_}]_2_ (**7**) in a 45 % isolated yield (Scheme 2). It is germane to note that Et_3_Al⋅DABCO can catalyse hydroboration of alkynes due to a redistribution reaction with HBpin generating the active Et_2_AlH species.^6^ The structure of **7** (Figure 2, left) reveals a dimer wherein both *i*Bu^−^ groups of **4** have been replaced, by Ph_2_(H)CO^−^ ligands, formed by an apparent β‐hydride process from the parent complex. β‐Hydride elimination is known in alkyl‐aluminum chemistry with carbonyls,^11^ but to our knowledge this is the first example in hydroboration catalysis used to generate a transient aluminum hydride. Thus **4** may be considered a masked hydride complex in hydroboration of ketones. Elaborating this step further, it is pertinent to consider the Meerwein–Ponndorf–Verley (MPV) reduction,11a, 12, 13 employing aluminum alkoxides as the hydride source to reduce ketones. Two competing mechanisms have been studied in silico.^13^ The first involves β‐hydride transfer from the alkoxide ligand giving a high energy Al–H intermediate, which can then follow the pathway represented in Scheme 1. The second pathway is much lower in energy and describes a concerted process containing a 6‐membered transition state, facilitating direct hydride transfer to the substrate.

**Figure 2:**
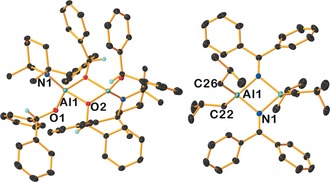
Molecular structures of **7** (left) and **8** (right). All hydrogen atoms are omitted for clarity except those on the reduced benzophenone anions. Thermal ellipsoids are set at 30 % probability. See Supporting Information for crystallographic details and CCDC numbers.

**Scheme 2:**
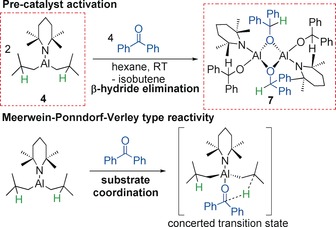
Top: Reaction between **4** and benzophenone, revealing formation of the active catalytic species **7** via β‐hydride elimination. Bottom: Reaction between **4** and benzophenone proceeding via a possible Meerwein–Ponndorf–Verley type reaction.

**Table 1:** Hydroboration catalysis results for carbonyls, imines and acetylenes using **1**–**6** as catalysts.^[a]^

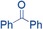	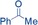	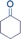
**1** ^[b]^ 99 % 0.5 h; **4** 94 % 0.5 h **2** ^[c]^ 99 % 0.5 h; **5** 69 % 5 h **3** 79 % 0.5 h; **6** 17 % 4 h	**1** ^[b]^ 97 % 2 h; **4** 40 % 6 h **2** ^[c]^ 80 % 3 h; **5** 55 % 5 h **3** 99 % 0.5 h	**1** ^[b]^ 93 % 2.5 h; **4** 53 % 2 h **2** ^[c]^ 91 % 2 h; **5** 71 % 1 h **3** 98 % 0.25 h
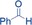	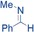	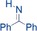
**1** ^[b,c]^ 99 % 0.25 h; **4** 79 % 1 h **2** ^[c]^ 81 % 2 h; **5** 88 % 0.25 h **3** 99 % 0.25 h	**1** 42 % 2 h; **4** 3 % 2 h **2** 35 % 2 h; **5** 22 % 2 h **3** 53 % 2 h; **6** 5 % 2 h	**1** 73 % 0.5 h; **4** 34 % 5 h **2** 78 % 0.75 h; **5** 56 % 4 h **3** 80 % 0.5 h; **6** 33 % 4 h
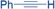	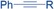	
**1** 71 % 2 h; **4** 0 % 17 h **3** 83 % 2 h; **6** ^[d]^85 % 2 h	R=Ph: **1** 0 %, 2 h **3** 10 %, 2 h; **6** ^[d]^ 40 % 2 h R=Me, **3** 60 % 2 h (2.2:1 ratio); **6** ^[d]^ trace	

[a] Aldehyde/ketones 5 mol % [Al] cat. loading, C_6_D_6_, room temperature. Imines 10 mol % [Al] cat. loading, C_6_D_6_, room temperature. Alkynes 10 mol % [Al] cat. loading, in [D_8_]toluene at 110 °C. [b] data for **1** from Ref. 8b. [c] data for **2** (1 mol % cat.) from Ref. 8a. [d] data for **6** from Ref. 6; All yields against ^1^H NMR internal standard hexamethylcyclotrisiloxane.

Compound **7** (2.5 mol %) is shown to be catalytically active in a reaction with benzophenone and HBpin, where quantitative hydroboration occurs after 3 hours. Since **4** seems a reactivity outlier, showing comparable reactivity to **1**, they were both screened catalytically with one aldehyde and two further ketones. In each case the bimetallic complex **1** showed far superior activity.

Furthermore, a control reaction employing LiH as a catalyst (5 mol %) for hydroboration of benzophenone gave a yield of only 10 % after 4 h. This illustrates that, in this regard, the neutral aluminum or lithium reagents in isolation deliver markedly reduced reactivities compared with the bimetallic formulations. Importantly, for the first time direct competition experiments reveal the synthetic superiority of lithium aluminate complexes in the context of hydroboration.

Hypothesising that any “ate effect” would be magnified with more challenging substrates we turned our attention to imines, which hitherto have not been catalytically hydroborated with Al complexes. That said, examples exist of main group complexes catalysing this transformation, and of Al complexes catalysing hydrosilylation or hydrogenation of imines,2c, 10e, 14 suggesting that imine hydroboration is a viable synthetic target.

Catalytic hydroboration reactions of N‐benzylidenemethylamine, using **1**–**6** showed lower reactivity at room temperature than with aldehydes and ketones, however the same reactivity pattern emerges, in that the bimetallic complexes are superior to monometallic counterparts. After two hours, conversions are with **1** (42 %), **2** (35 %), **3** (53 %), **4** (3 %), **5** (22 %) and **6** (5 %). Nevertheless, these results with **1**–**3** constitute the first use of Al complexes in imine hydroboration. Stoichiometric reactions between **1**, **3**, **4** and **6** with the imine provide further insight. Compound **4** forms only a coordination adduct with the imine in contrast to the β‐hydride elimination product with benzophenone, whereas, **1**, **3** and **6** add across the C=N double bond, with **6** displaying higher insertion reactivity (see Supporting Information). Notably Stephan and co‐workers reported a dimeric structure of an analogous reaction between **6** and a related imine.14a However, faster substrate insertion does not translate into fast catalytic transformation. Thus we infer that the σ‐bond metathesis step with HBpin is greatly facilitated by the additional polarity imposed by the bimetallic ate constitution. Reinforcing this hypothesis, Harder and co‐workers’ imine hydrogenation using catalytic LiAlH_4_ illuminates the important role of the alkali metal, via DFT studies, wherein Al‐H‐Li interactions are retained throughout the proposed catalytic cycle.14d

We next screened benzophenone imine in the catalysis with **1**, **3**, **4** and **6** (10 mol %), since this substrate has an acidic N‐H atom amenable to deprotonation and therefore provides the possibility of reaction proceeding via an alternative deprotonation pathway. Furthermore, amido groups in **1** and **4** can be directly compared with alkyl groups in **3** and **6. 1** and **3** achieve 73 % and 80 % conversion after 2 h or 30 minutes, respectively. Compounds **4** and **6** perform poorly, showing no catalytic activity at room temperature, prompting further consideration. Two stoichiometric reactions between benzophenone imine and **1**, and **4** were conducted, wherein both exhibit amido basicity. In the reaction with **4** [*i*Bu_2_Al(μ‐N=CPh_2_)]_2_ (**8**; Figure 2, right), was isolated as single crystals in a 24 % yield (^1^H NMR yield of 86 % against hexamethylcyclotrisiloxane as internal standard). In contrast to the benzophenone case where catalysis proceeds after a β‐hydride process step, the reactivity here ceases after an initial deprotonation by the TMP basicity. Interestingly, both **3** and **6** display trace amounts of H_2_ evolution in their catalytic reactions as evidenced by a low intensity singlet resonance in the respective ^1^H NMR spectra at *δ* 4.47 ppm.

The catalytic results with benzophenone imine merit further comment. Both **1** and **4** exhibit deprotonation, suggesting that in a catalytic regime, reaction (using **1**) may proceed in the pathway outlined in Scheme 3, that is, deprotonation followed by hydroboration then protonolysis to liberate product and generate a catalytically active species.

**Scheme 3:**
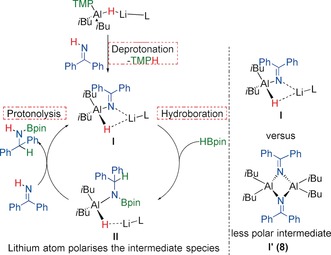
Left: Suggested mechanism for hydroboration of benzophenone imine catalysed by **1**. Right: Alternative intermediate for catalytic profile using **4**. See text for details.

That **1** is active and **4** is not, may be assigned to the nature of deprotonation products, which clearly demonstrates the key role of bimetallic (Li–Al) cooperativity. **I** is the proposed deprotonation intermediate using **1** and **I′** using **4**, which corresponds to the crystallographically authenticated **8**. In **I** the alkali metal would instil a different molecular charge distribution to that in **I′**. This scenario clearly facilitates the hydroboration step, which is not the case with **I′**. A final comment on imine hydroboration is that in both cases **1** (73 % 0.5 h) offers marginally less reactivity than **3** (80 % 0.5 h). This difference may describe a subtle alkyl versus amido effect, whereby the replacement of one TMP anion for an *i*Bu anion imparts greater nucleophilicity onto the hydride, priming it for addition across the unsaturated substrate. Alternatively, the increased steric demand of TMP may slow reactivity. Moreover, it is apparent that even when the deprotonation pathway is available (catalyst **1** with benzophenone imine), the pathway that follows, insertion (catalyst **3** with benzophenone imine) is favoured, albeit marginally.

Finally, we turned to acetylene hydroboration comparing reactivity once more between **1**, **3**, **4** and **6**. Stoichiometric reactions of TMP‐containing **1** and **4** with terminal alkyne phenylacetylene (PhCCH) in C_6_D_6_, reveal deprotonation of PhCCH at room temperature, in agreement with the fact that hydroboration of PhCCH with **1** implicated deprotonation as a key step.8b Alternatively **3** is unreactive with PhCCH, and **6** only very slowly hydroaluminates PhCCH, at room temperature. Catalysis, using 10 mol % loadings in [D_8_]toluene at 110 °C, in line with the reported reaction conditions using **6** (85 % conversion after 2 hours),^6^ reveal that **1** and **3** catalyse the transformation to the anti‐Markovnikov vinylboronate ester in yields of 71 % and 83 % respectively. Conversely, **4** as expected, does not function as a catalyst. Thus **3** is comparable to **6** however, for the first time we note that a clear ate effect is not in operation. Furthermore, **3** is a better catalyst than **1** underlying that increased hydride nucleophilicity is more important, mechanistically, than deprotonation, though reduced steric effects may also be a factor.

A similar picture is seen with the internal alkyne diphenylacetylene. **6** (10 mol %) is reported to convert diphenylacetylene to the boronic ester in 40 % yield after 2 hours at 110 °C in [D_8_]toluene,^6^ whereas **1** is completely inactive, and **3** only reaches conversions of approximately 10 % after 2 hours, which is surprising given our preceding observations. One potential rationale for this marked reduction in ate reactivity with diphenylacetylene may be attributed to a steric effect (Scheme 4).

**Scheme 4:**
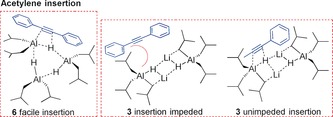
Comparative insertion profiles for reaction of diphenylacetylene with **6** and **3** and of 1‐phenyl‐propyne with **3**.

Considering the required initial insertion step at the sp‐C of diphenylacetylene, insertion into the Al−H bond of **3** (three *i*Bu groups, one hydride) is likely to be slower than for **6** (two *i*Bu groups, one hydride) due to the inherently more sterically demanding ate constitution, even given the trimeric solution constitution of **6** (via DOSY NMR spectroscopy, see Supporting Information). Clearly, with ketones and imines any insertion step at the sp^2^ O/N would be considerably less congested, thus facile insertion would occur, thereby facilitating the ate enhancement seen in the ensuing hydroboration catalysis. Elaborating further, we attempted one further substrate in comparative catalytic experiments with **6** and **3**. With **6**, 1‐phenyl‐propyne is only hydroborated in trace amounts, despite the intrinsically smaller CH_3_ group with respect to diphenylacetylene.^6^ On the other hand, **3** catalyses the transformation to a mixture of regio‐isomers (60 % conversion overall) in favour of borylation at the least sterically hindered alkyne carbon atom, demonstrating once more the advantage of ate complexes in these catalytic transformations.

This study into hydroboration of aldehydes, ketones and imines reveals that anionic ate complexes are important additions to the main‐group catalyst toolbox, providing higher conversions in shorter timescales. We attribute this superiority to the greater polarisation of key reaction intermediates induced by the heterobimetallic complexes. Moreover, a novel new catalytic activation pathway was elucidated for ketone hydroboration involving a β‐hydride process. With internal alkynes the scenario is different and mononuclear species are the catalysts of choice when steric constraints override the ate effect. Overall this study illuminated that while ate complexes are beneficial in most cases, the mononuclear species are more effective in others. Thus, in the field of aluminum‐catalysed hydroelementation, there is a high degree of substrate dependence, governing the appropriate choice of catalyst.

## Conflict of interest

The authors declare no conflict of interest.

## Supporting information

As a service to our authors and readers, this journal provides supporting information supplied by the authors. Such materials are peer reviewed and may be re‐organized for online delivery, but are not copy‐edited or typeset. Technical support issues arising from supporting information (other than missing files) should be addressed to the authors.

Supplementary
