# Supplementary material for: Comparing Neutral (Monometallic) and Anionic (Bimetallic) Aluminum Complexes in Hydroboration Catalysis: Influences of Lithium Cooperation and Ligand Set
Source: Angew Chem Int Ed Engl. 2018 Jul 9;57(33):10651–5. doi: 10.1002/anie.201806168 (PMC6099445; doi:10.1002/anie.201806168)
Supplement: Supplementary file 1 — Supplementary [file ANIE-57-10651-s001.pdf]

Angewandte Chemie
Eine Zeitschrift der Gesellschaft Deutscher Chemiker

Supporting Information

Comparing Neutral (Monometallic) and Anionic (Bimetallic) Aluminum Complexes in Hydroboration Catalysis: Influences of Lithium Cooperation and Ligand Set

Victoria A. Pollard, M. Ángeles Fuentes, Alan R. Kennedy, Ross McLellan, and Robert E. Mulvey**

anie_201806168_sm_miscellaneous_information.pdf

Supplementary information

Contents

General Experimental: 1

Catalysis Reactions: 1

NMR spectroscopy 1

X-ray crystallography 1

Synthesis of Compounds: 2

Synthesis of $[iBu_3Al(\mu\text{-}H)Li]_2$, 3 2

Synthesis of $[(TMP)(Ph_2(H)CO)Al(\mu\text{-}OC(H)Ph_2)]_2$, 7 4

Synthesis of $[iBu_2Al(\mu\text{-}NCPh_2]_2$, 8 6

^1H DOSY NMR Spectra for DIBAL in d_8-toluene: 9

^1H DOSY NMR Spectra for DIBAL in C_6D_6: 10

Stoichiometric Reactions of Carbonyls: 11

Hydroboration of Carbonyls 14

Stoichiometric Reactions of Imines: 26

Hydroboration of Imines 33

Stoichiometric Reactions of Alkynes: 40

Hydroboration of Alkynes 43

Table S1: Crystal structure data and refinement details for compounds 7 and 8 48

General Experimental:

All reactions and manipulations were performed under a protective argon atmosphere using either standard Schlenk techniques or glove box techniques. Hexane, THF, diethyl ether and toluene were dried by heating to reflux over sodium benzophenone ketyl and then distilled under nitrogen prior to use. C_6D_6, d_8-toluene and d_8-THF were degassed by freeze-pump-thaw methods and stored over activated 4 Å molecular sieves. All reagents were purchased from commercial sources and used as received, unless stated otherwise. TMEDA, PMDETA and HMDS(H) were distilled and stored over activated 4 Å molecular sieves prior to use. $iBu_2Al(TMP)$,[1] $(HMDS)_2AlH$,[2] and $[iBu_2Al(TMP)(H)Li]_2$,[3] were prepared according to literature methods.

Catalysis Reactions:

The desired catalyst **1** – **6** at the desired catalyst loading was added to 0.5 mL of a deuterated NMR solvent (C_6D_6, or d_8-toluene as appropriate) solution containing 0.5 mmol of the substrate precursor and of pinacolborane (80 µL, 0.55 mmol), as well as 10 mol% of internal standard; hexamethylcyclotrisiloxane. The mixture was transferred to a sealed J. Young's tap NMR tube and the reaction was regularly monitored by ^{1}H and ^{11}B NMR until the formation of products was completed. The yields reported are based on ^{1}H NMR relative to the internal standard. In all cases, the bulk of the NMR solution can be attributed to either the boronate esters or starting material.

NMR spectroscopy

NMR spectra were recorded on a Bruker AV3 or AV 400 MHz spectrometer operating at 400.13 MHz for ^{1}H, 155.47 MHz for ^{7}Li, 104.2 MHz for ^{27}Al, 128.3 MHz for ^{11}B and 100.62 MHz for ^{13}C. All ^{13}C spectra were proton decoupled. ^{1}H, $^{13}C\{^{1}H\}$, ^{7}Li, and ^{11}B chemical shifts are expressed in parts per million (δ, ppm) and referenced to residual solvent peaks. DOSY measurements were recorded on an AV 400 MHz spectrometer operating at 400.13 MHz, using the pulse program ledbpgp2s. Tetraphenylnaphthalene has been used as reference in the DOSY measurements.

X-ray crystallography

Data for **7** and **8** were collected on Oxford Diffraction Gemini S or Xcalibur E instruments with graphite-monochromated Mo Kα (λ = 0.71073 Å) or Cu Kα (λ 1.54184 Å) radiation. Data collection and processing used Rigaku and Bruker software.[4, 5] All structures were solved and refined to convergence on F 2 for all independent reflections by the full-matrix least squares method using SHELXL-2014/7[4, 6] , or by the Gauss Newton algorithm using OLEX2.[7] All non-hydrogen atoms were refined using anisotropic thermal parameters. Selected crystallographic data are shown in Table S1 and full details in .cif format are available from CCDC (1845056 and 1845057).

Synthesis of Compounds:

Synthesis of $[iBu_3Al(\mu\text{-}H)Li]_2$, **3**

To a solution of DIBAL-H (1 M/hexane; 5 mL; 5 mmol) in hexane (5 mL) was added iBuLi (1.7 M/heptane; 5 mmol; 2.9 mL). Spontaneous precipitation of the product was observed. The white suspension was stirred at room temperature for 1 hour, then the product isolated by filtration. (0.816 g; 3.96 mmol; 89% yield).

^{1}H NMR (400.1 MHz, C6D6, 300 K): δ -0.003 (d, J = 6.64 Hz, 6H CH_2); 1.17 (d, J = 6.69 Hz, 18H CH_3); 2.01 (sept, J = 6.92 Hz, 3H, CH) ppm.

^{13}C{^{1}H} NMR (100.6 MHz, C6D6, 300 K): δ 22.9 (s, CH_2); 27.8 (s, CH); 28.7 (s, CH_3) ppm.

^{7}Li NMR (155.5 MHz, C6D6, 300 K): δ -2.42 (s) ppm.

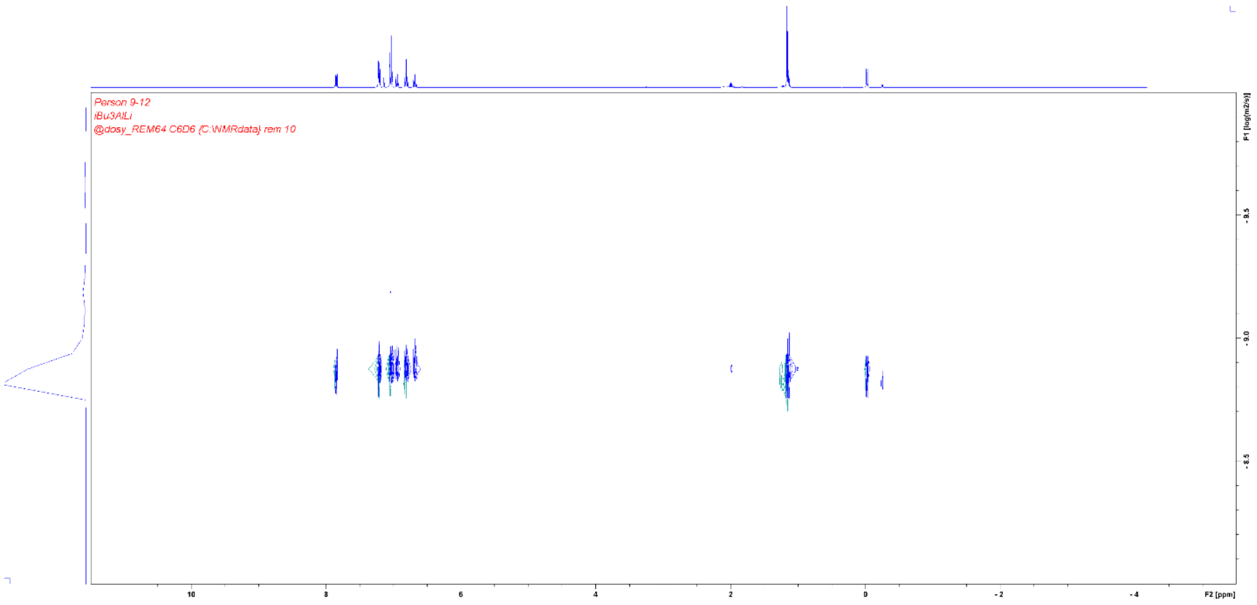

Figure S1: ^{1}H DOSY NMR for **3** with internal standard tetraphenylnaphthalene, in C_6D_6.

**Compound**	**D [m²s⁻¹]**	**MW$_{calc}$ [g mol^{-1}]**	**MW$_{est}$ [g mol^{-1}]**	**Error %**
$[iBu_3AlHLi]_2$	$1.217x10^{-9}$	412.44	465	-7
Tetraphenylnaphthalene	$1.211X10^{-9}$	432.55	465	-11

The diffusion coefficient of iBu_3AlHLi indicates a molecular weight of 465 g mol^{-1} . This result is consistent with the calculated molecular weight for the dimeric structure $[iBu_3AlHLi]_2$.

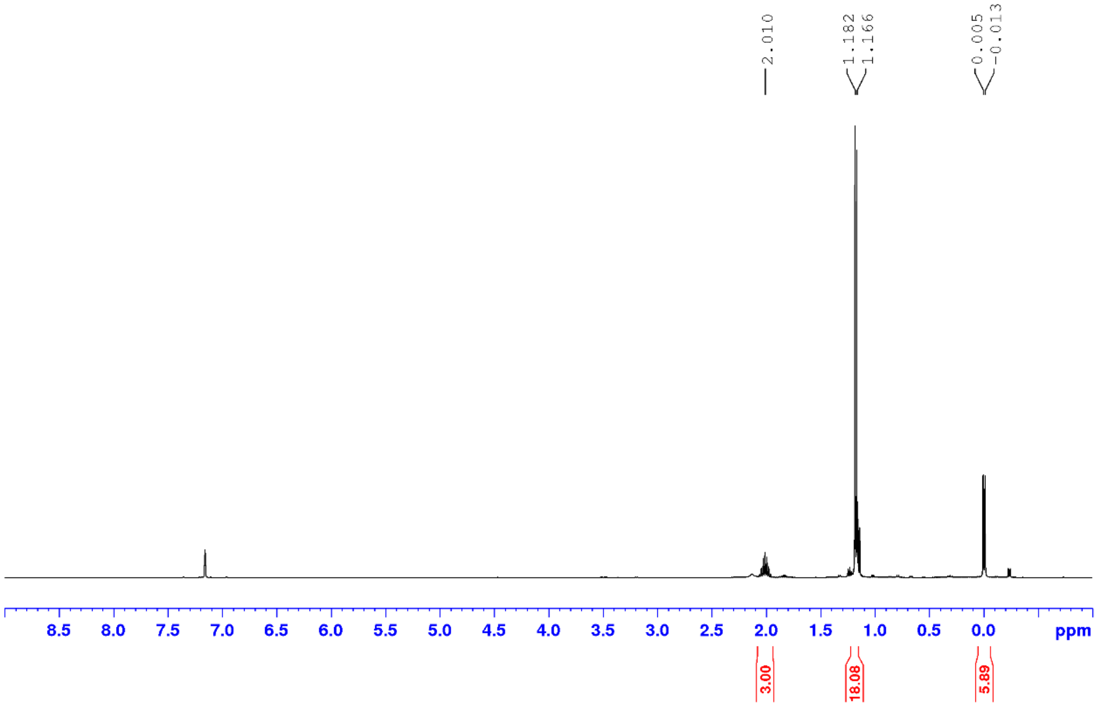

Figure S2: ^{1}H NMR spectrum of $[iBu_3Al(\mu\text{-}H)Li]_2$, **3**, in C_6D_6

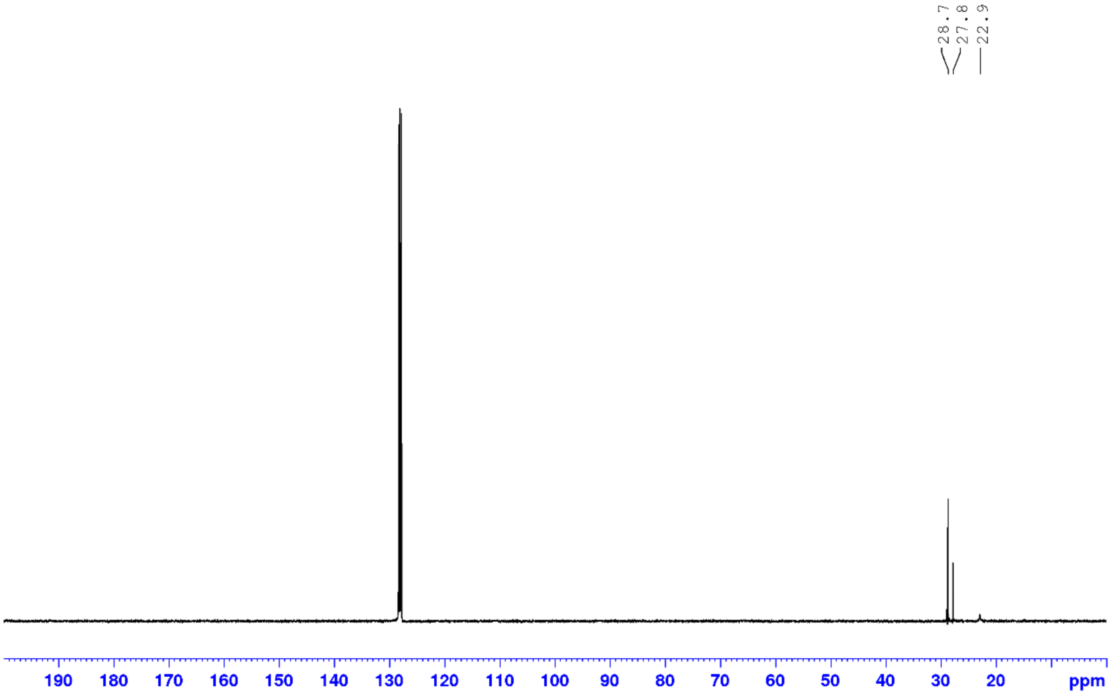

Figure S3: $^{13}C\{^{1}H\}$ NMR spectrum of $[iBu_3Al(\mu\text{-}H)Li]_2$, **3**, in C_6D_6

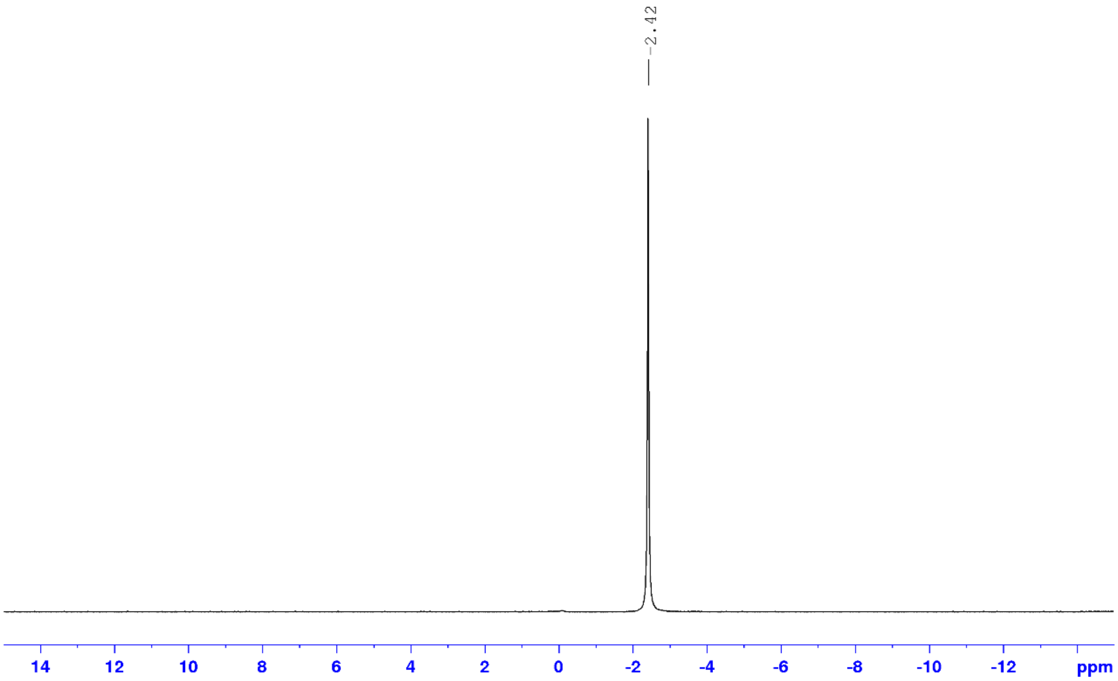

Figure S4: ^{7}Li NMR spectrum of $[iBu_3Al(\mu\text{-}H)Li]_2$, **3**, in C_6D_6

Synthesis of $[(TMP)(Ph_2(H)CO)Al(\mu\text{-}OC(H)Ph_2)]_2$, 7

To a solution of $iBu_2Al(TMP)$ (0.84 g; 3 mmol) in hexane (10 mL) was added benzophenone (0.5462 g; 3 mmol) and the reaction solution turned immediately purple. The reaction was stirred at room temperature for 15 minutes during which time the colour dissipated. Subsequent standing at room temperature overnight yielded a crop of colourless crystals (0.78 g; 0.66 mmol; 45 % yield).

^{1}H NMR (400.1 MHz, C6D6, 300 K): δ 0.99 (t, J = 6.65 Hz, 8H, 4 x TMP β-CH_2); 1.29 (s, 24 H 4 x TMP Me); 1.44 (m, 4H, 2 x TMP ɣ-CH_2); 6.68 + 6.80 (s, 2 H each, 2 x OC*H*Ph_2 each); 6.93 – 6.98 (m, 12H, Ar-C*H*); 7.08 (t, J = 7.35 Hz, 4 H, Ar-C*H*); 7.20 – 7.22 (m, 4H Ar-C*H*); 7.29 (t, J = 7.58 Hz, 8 H, Ar-C*H*); 7.51 – 7.53 (m, 4 H Ar-C*H*); 7.88 (d, J = 7.17 Hz, 8 H Ar-C*H*) ppm.

^{13}C{^{1}H} NMR (100.6 MHz, C6D6, 300 K): δ 17.8 (s, TMP ɣ-CH_2); 35.0 (s, TMP CH_3); 39. 5 (s, TMP β-CH_2); 51.7 (s, TMP $\underline{C}(CH_3)_2$); 80.1 (s, O$\underline{C}$(H)Ph_2); 81.0 (s, O$\underline{C}$(H)Ph_2); 127.5 + 128.0 + 128.7 + 128.9 + 129.0 + 129.5 (s, Ar-$\underline{C}$H); 141.1 (s, ipso Ar-C); 142.6 (s, ipso Ar-C) ppm.

Elemental analysis (%) for $C_{70}H_{80}Al_2N_2O_4$: calc'd C 78.77 H 7.55 N 2.62; found: C 78.25 H 7.58 N 2.85

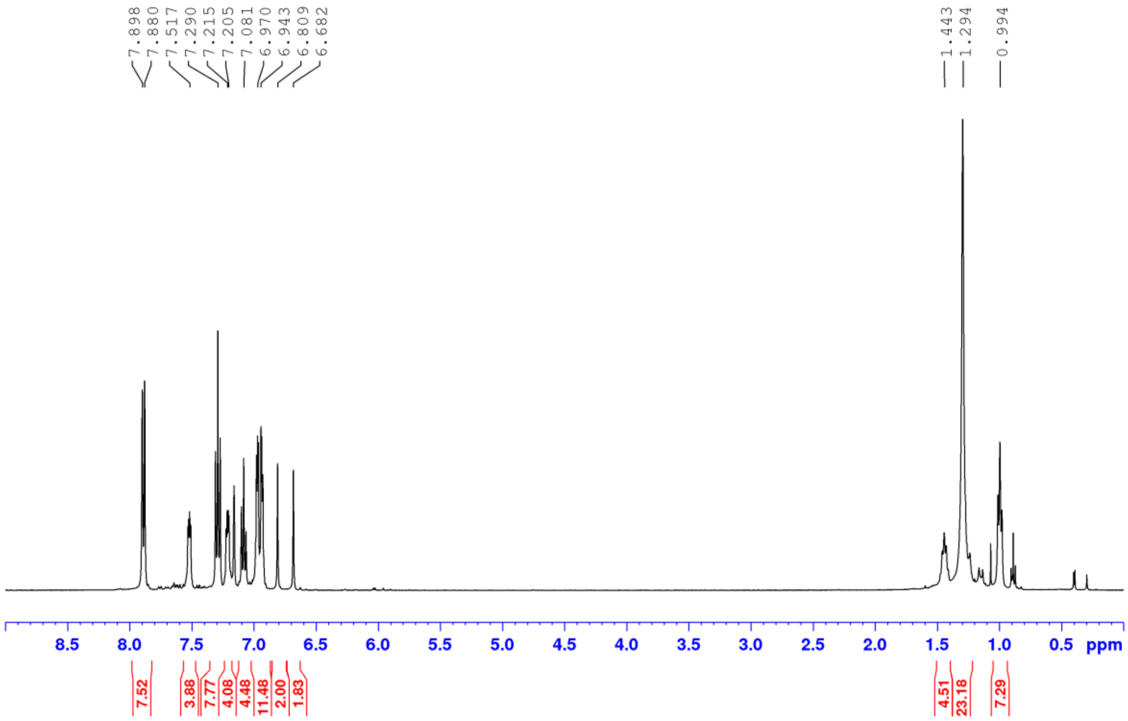

Figure S5: ^{1}H NMR spectrum of [(TMP)(Ph_2(H)CO)Al(μ-OC(H)Ph_2)]$_2$, **7**, in C_6D_6

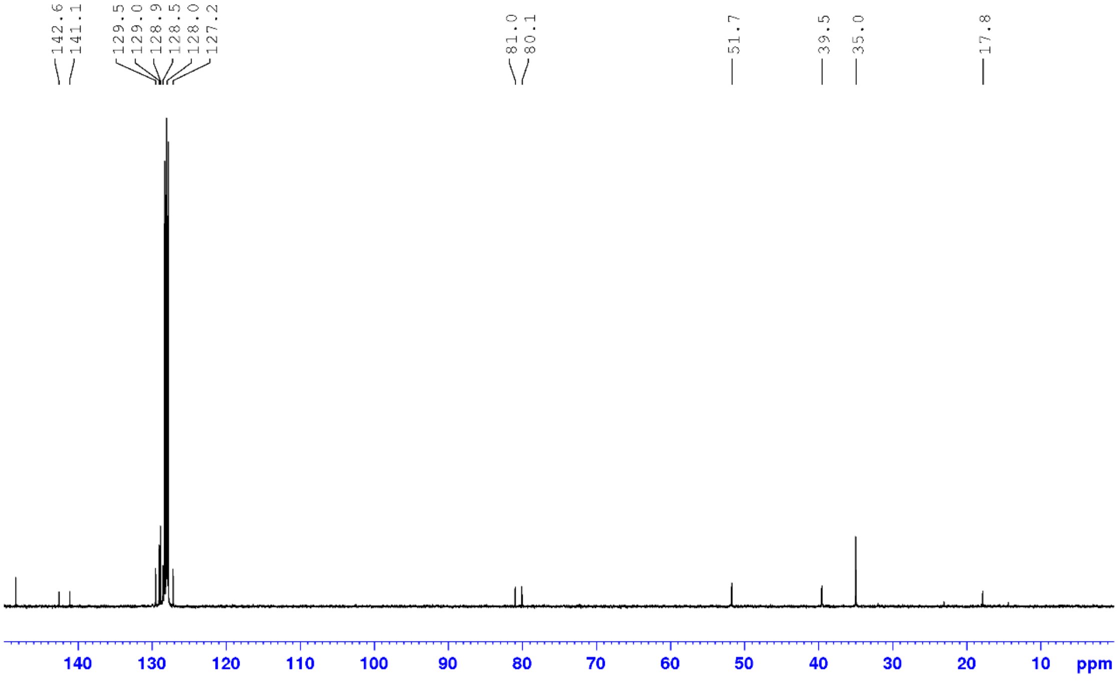

Figure S6: $^{13}C\{^{1}H\}$ NMR spectrum of [(TMP)(Ph_2(H)CO)Al(μ-OC(H)Ph_2)]$_2$, **7**, in C_6D_6

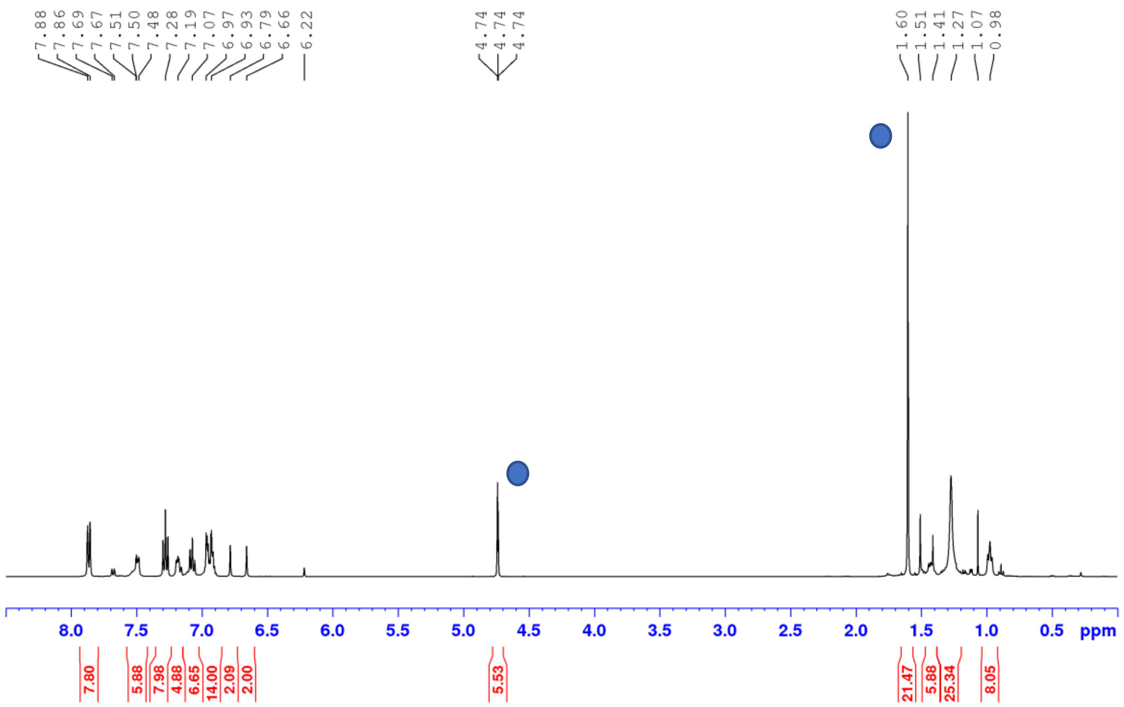

Figure S7: ^{1}H NMR spectrum from J. Young's NMR experiment for the formation of **7** from reaction of $iBu_2Al(TMP)$ + 2 equivalents benzophenone in C_6D_6. The signals corresponding to isobutene have been labelled

Synthesis of $[iBu_2Al(\mu\text{-}NCPh_2]_2$, 8

To a solution of $iBu_2Al(TMP)$ (0.84 g;3 mmol) in hexane (10 mL) was added benzophenone imine (0.55 mL; 3 mmol). The reaction solution turned immediately bright yellow and was stirred for 1 h. Cooling at – 30 ^{o}C overnight provided yellow crystal suitable for X-ray diffraction. (0.468 g; 0.73 mmol; 24 % yield).

^{1}H NMR (400.1 MHz, C6D6, 300 K): δ 0.18 (d, J = 6.92 Hz, 8 H, CH_2); 1.01 (d, J = 6.41 Hz, 24 H, CH_3); 1.79 (nonet J = 6.65 Hz, 4 H, CH); 7.04 – 7.12 (m, 12 H, Ar-C*H*); 7.53 – 7.55 (m, 8 H, Ar-C*H*) ppm.

^{13}C{^{1}H} NMR (100.6 MHz, C6D6, 300 K): δ 25.6 (s, CH_2); 26.7 (s, CH); 28.7 (s, CH_3); 128.4 + 129.0 + 142.9 (s, Ar-C̲H); 142.9 (s, ipso Ar-C); 185.8 (s, C=N) ppm.

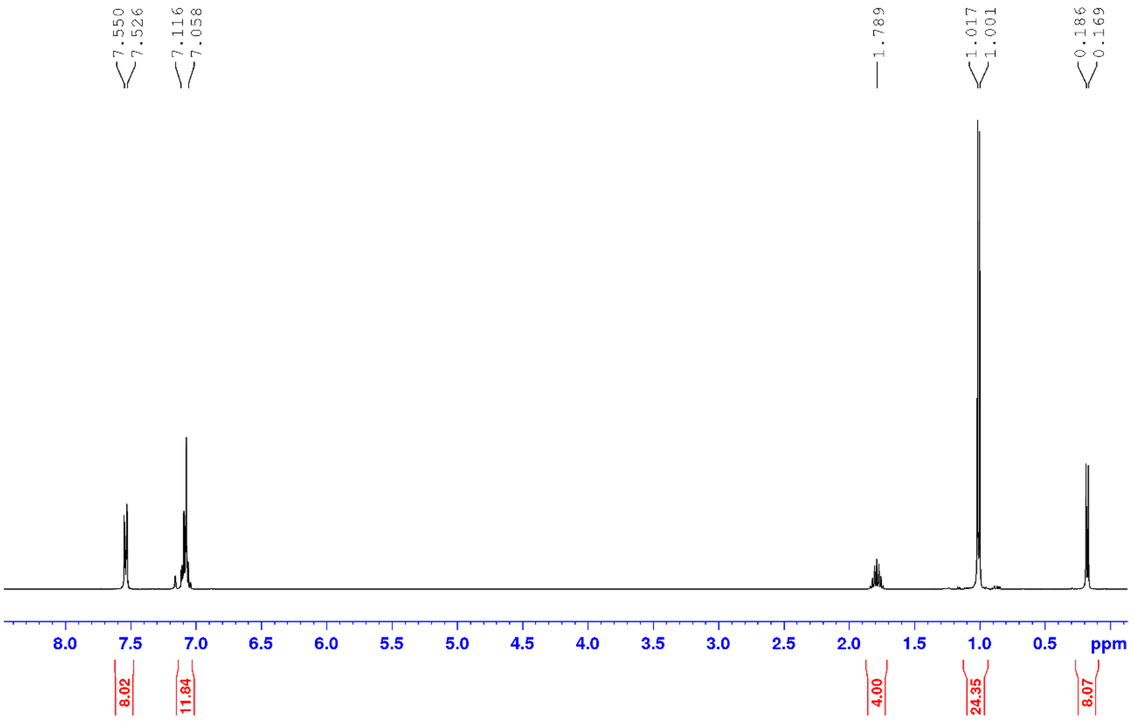

Figure S8: ^{1}H NMR spectrum of $[iBu_2Al(\mu\text{-}NCPh_2]_2$, **8**, in C_6D_6

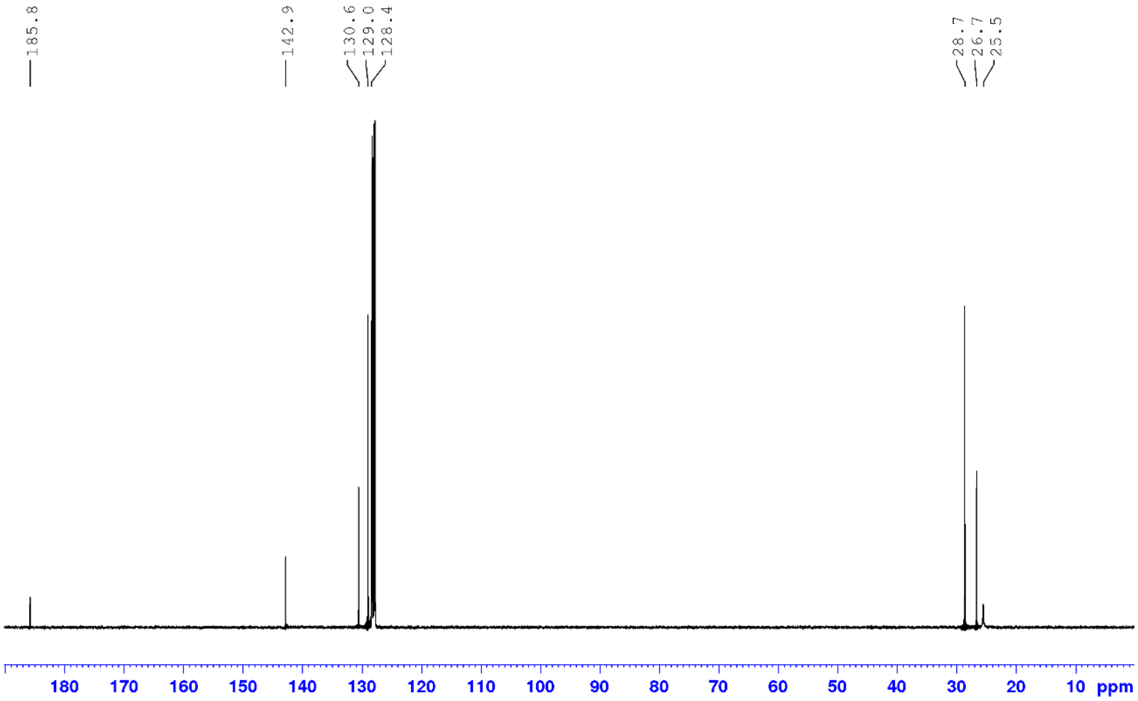

Figure S9: $^{13}C\{^{1}H\}$ NMR spectrum of $[iBu_2Al(\mu\text{-}NCPh_2]_2$, **8**, in C_6D_6

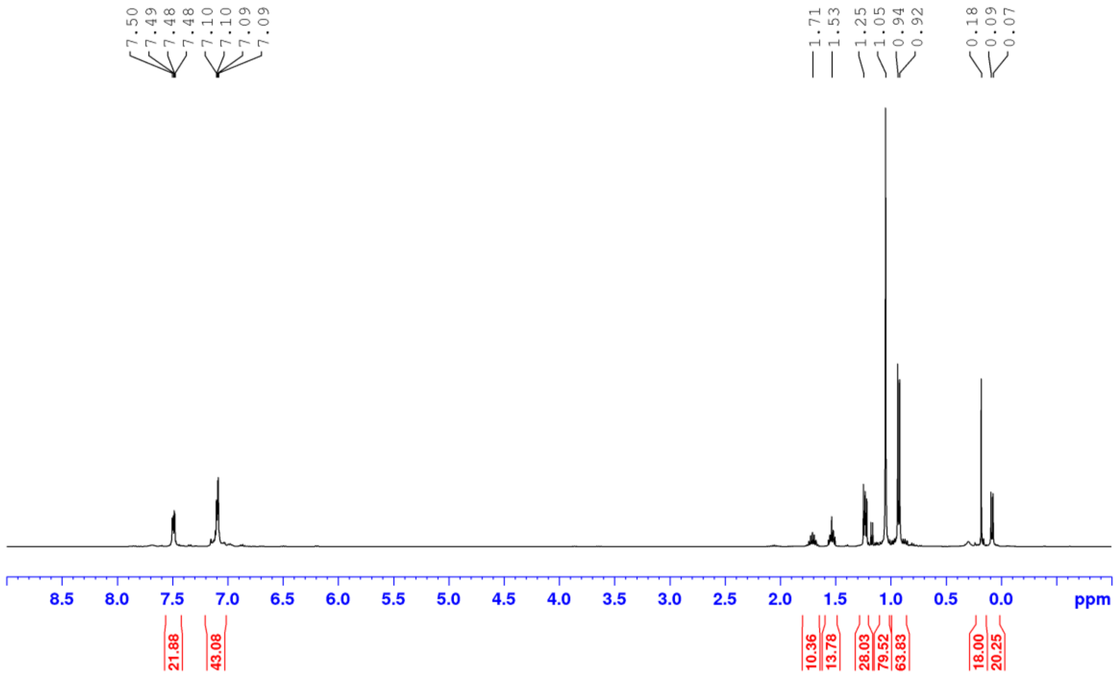

Figure S10: ^{1}H NMR spectrum for the J Young's NMR tube reaction between $iBu_2Al(TMP)$ and benzophenone imine in C_6D_6 to form $Bu2Al(\mu\text{-}NCPh_2]_2$, **8**. Gives an ^{1}H NMR yield of 86% with respect to internal ^{1}H NMR standard hexamethylcyclotrisiloxane.

^{1}H DOSY NMR Spectra for DIBAL in d$_8$-toluene:

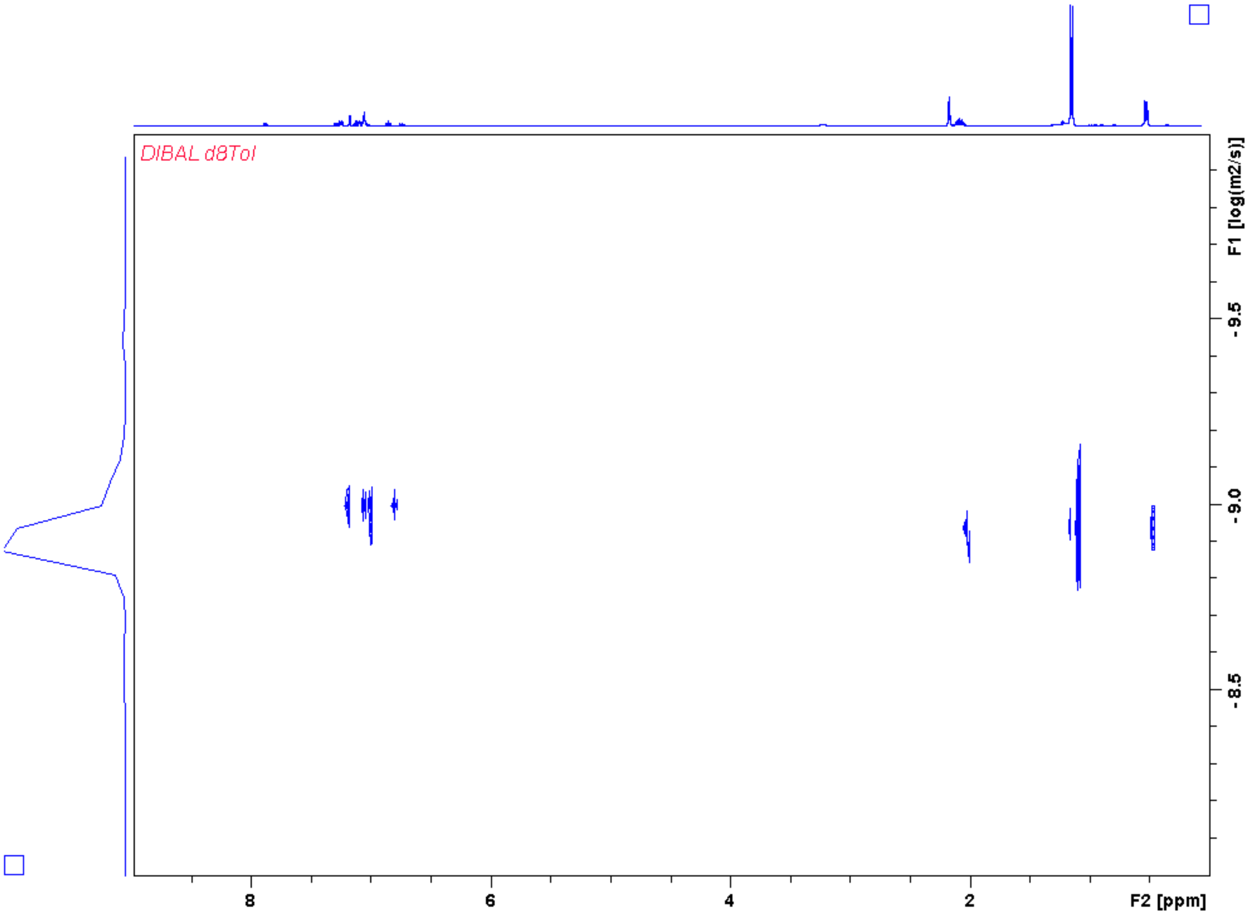

Figure S11: ^{1}H DOSY NMR spectrum for DIBAL in d$_8$tolene

Compound	D [m^2s^{-1}]	MW_{calc} [g mol^{-1}]	MW_{est} [g mol^{-1}]	Error %
DIBAL	$1.0897x10^{-9}$	142.13 (monomer)<br>426.39 (trimer)	401	6 %
Tetraphenylnaphthalene	$8.778x10^{-10}$	432.55	433	0%

The diffusion coefficient of DIBAL in d$_8$-toluene indicates a molecular weight of 401 g mol^{-1}. This result is consistent with the calculated molecular weight for a trimeric structure $(iBu_2AlH)_3$ = 426.39 g.

^{1}H DOSY NMR Spectra for DIBAL in C_6D_6:

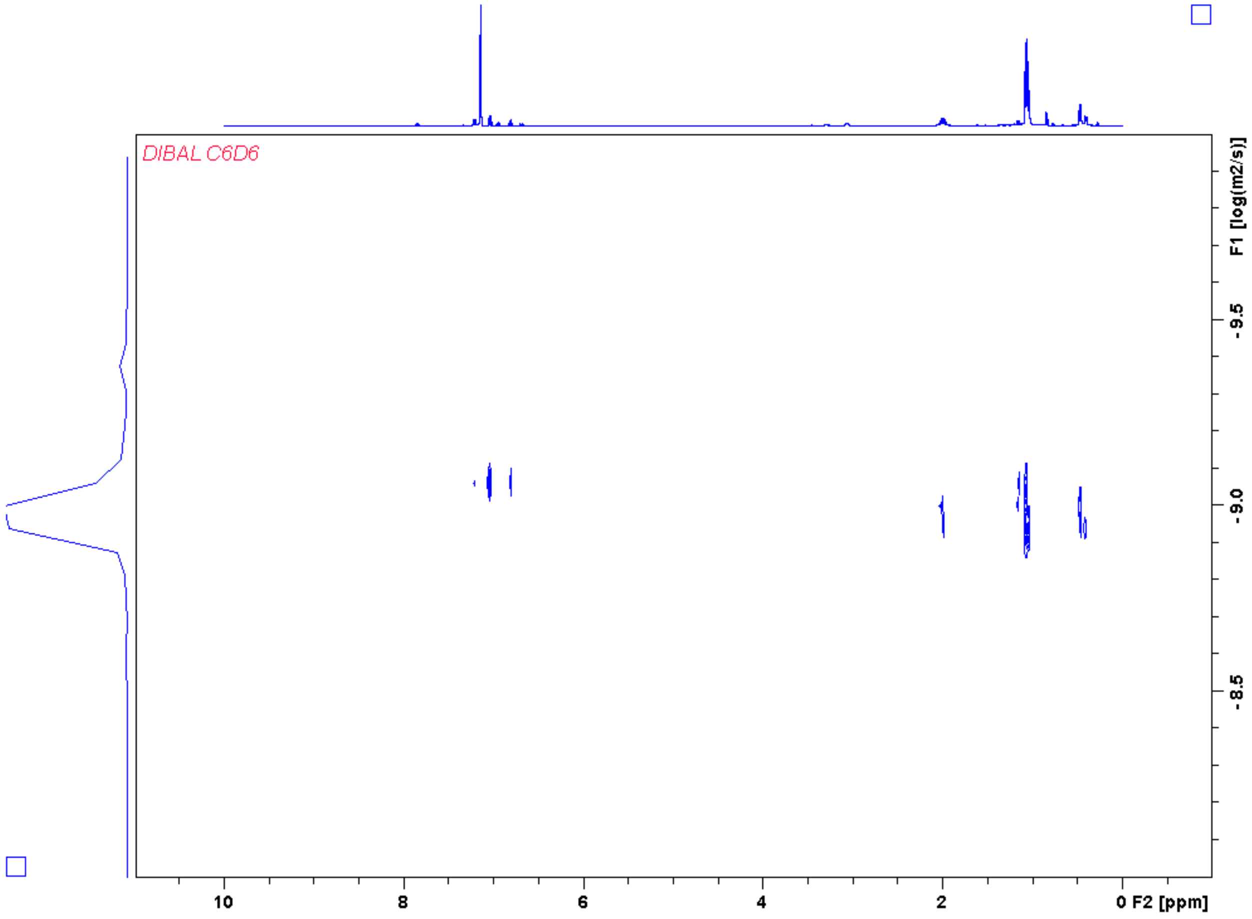

Figure S12: ^{1}H DOSY NMR spectrum for DIBAL in C_6D_6

Compound	D [m^2s^{-1}]	MW_{calc} [g mol^{-1}]	MW_{est} [g mol^{-1}]	Error %
DIBAL	$8.981x10^{-10}$	142.13 (monomer)<br>426.39 (trimer)	431	-1%
Tetraphenylnaphthalene	$8.162x10^{-10}$	432.55	433	0%

The diffusion coefficient of DIBAL in C_6D_6 indicates a molecular weight of 431 g mol^{-1}. This result is consistent with the calculated molecular weight for a trimeric structure $(iBu_2AlH)_3$ = 426.39 g.

Stoichiometric Reactions of Carbonyls:

Benzophenone + iBu$_3$Al(H)Li, **3**

In a J. Young's NMR tube iBu$_3$Al(H)Li (0.023 g; 0.125 mmol) and benzophenone (0.026 g; 0.125 mmol) were dissolved in C_6D_6 (0.5 mL).

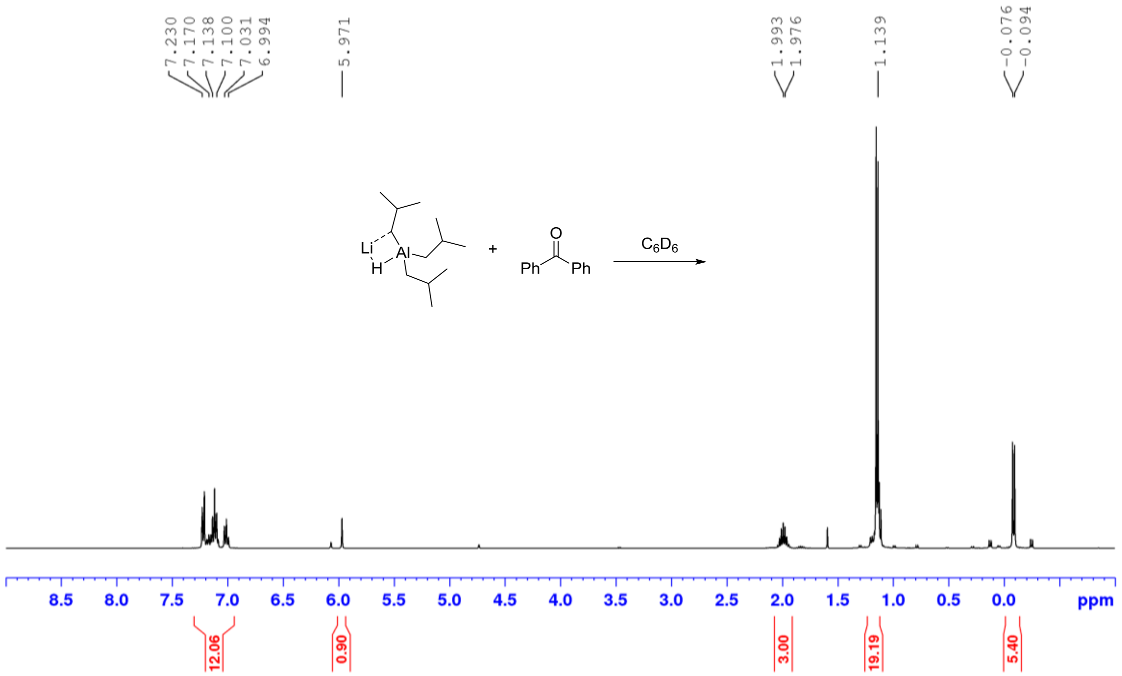

Figure S13: J Young's NMR reaction between benzophenone + [iBu$_3$Al(H)Li]$_2$, **3**, in C_6D_6 showing hydroelementation of the carbonyl.

Benzophenone + $HMDS_2AlH$, **5**

In a J. Young's NMR tube $HMDS_2Al(H)$ (0.087 g; 0.25 mmol) and benzophenone (0.046 g; 0.25 mmol) were dissolved in C_6D_6 (0.5 mL).

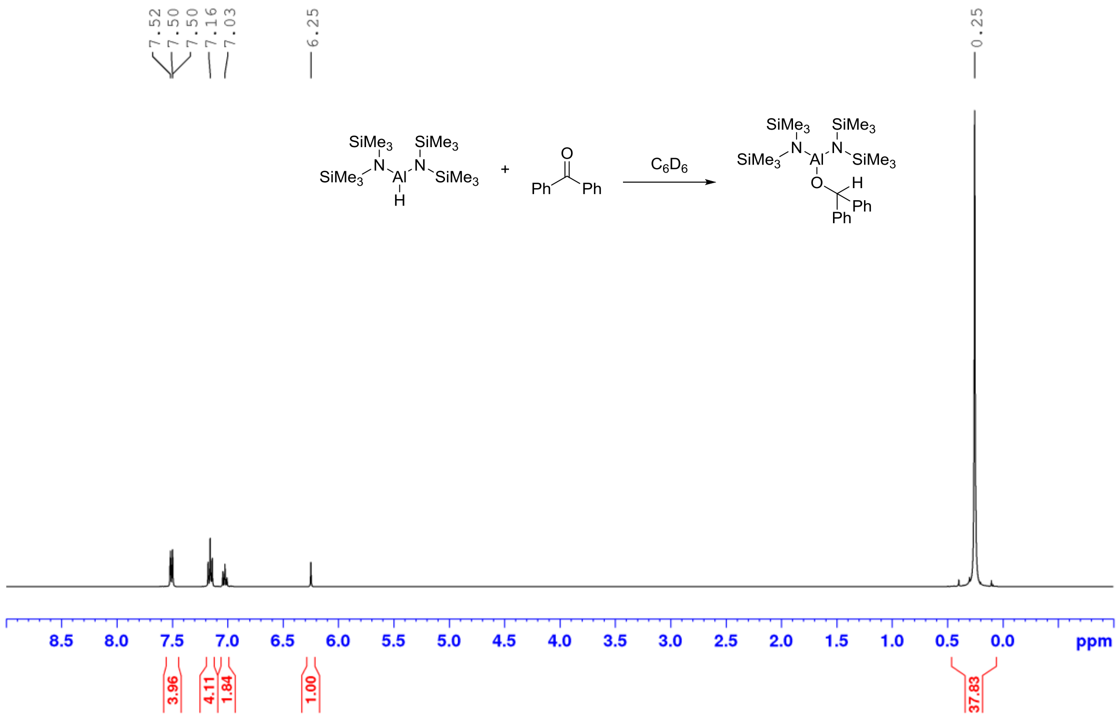

Figure S14: J Young's NMR reaction between benzophenone + $HMDS_2Al(H)$, **5**, in C_6D_6 showing hydroalumination

Benzophenone + DIBAL(H), 6

In a J. Young's NMR tube benzophenone (0.0911 g; 0.5 mmol) and DIBAL(H) (0.0711 g; 0.5 mmol) were dissolved in C_6D_6.

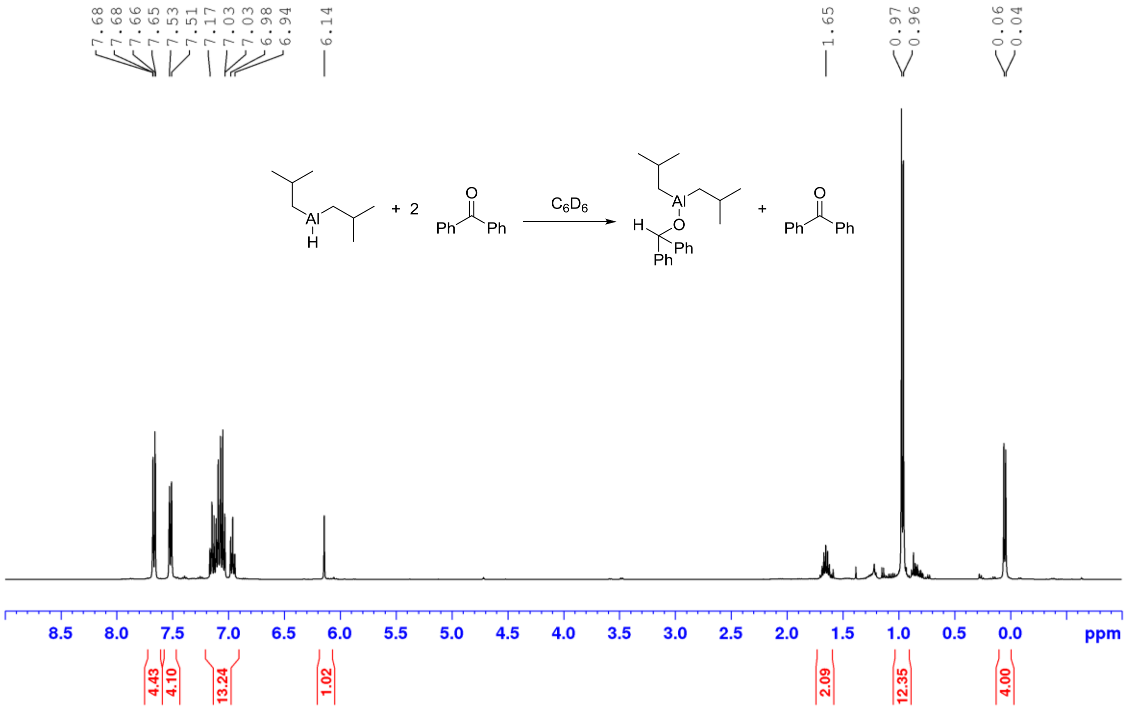

Figure S15: J Young's NMR reaction between 2 equivalents benzophenone + DIBAL(H), **6**, in C_6D_6 showing hydroalumination and unreacted benzophenone

Hydroboration of Carbonyls

All hydroboration reactions of carbonyl substrates were performed at 0.5 mmol scale, room temperature, in C_6D_6 solution with 5 mol% [Al] catalyst **1** – **7**, or 5 mol% LiH.

Benzophenone catalysed by $[iBu_3Al(H)Li]_2$, 3

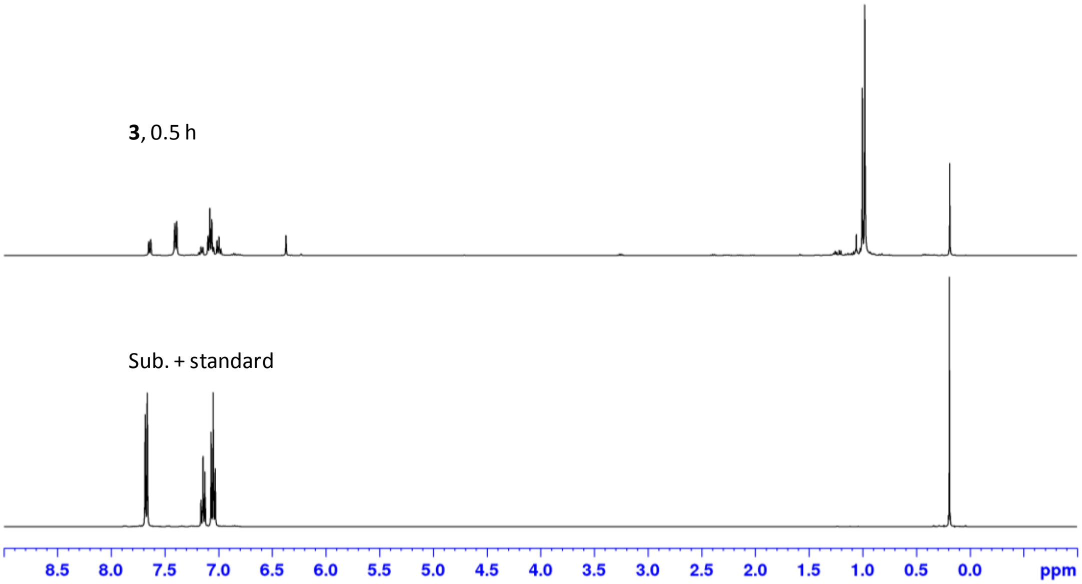

Figure S16: ^{1}H NMR spectrum for the hydroboration of benzophenone catalysed by **3** (5 mol% Al) in C_6D_6

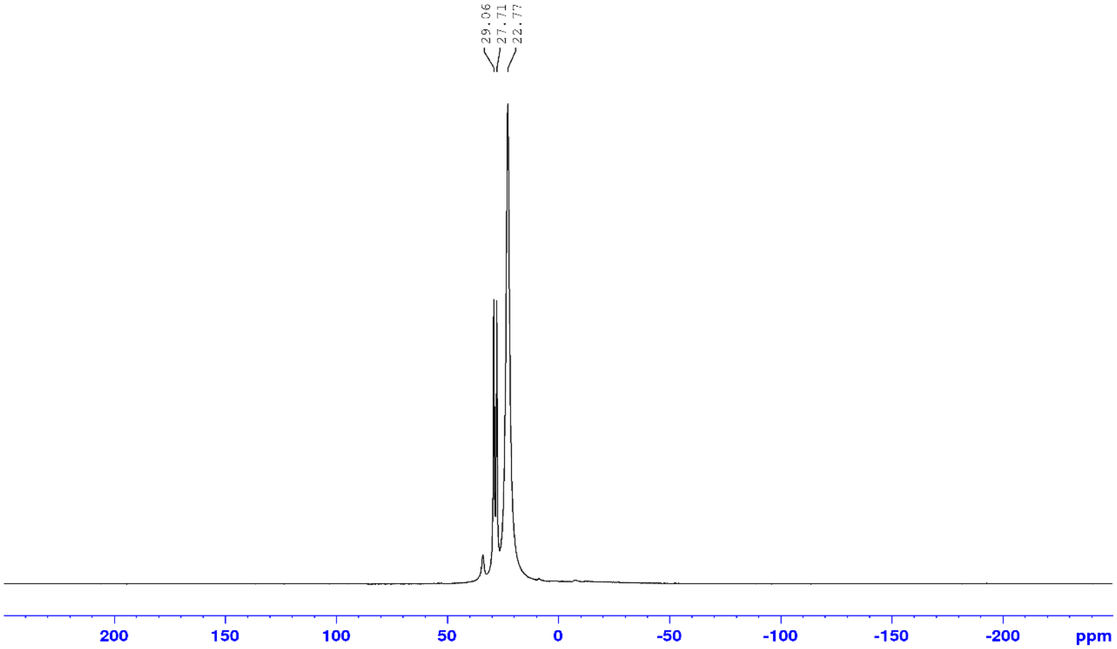

Figure S17: ^{11}B NMR spectrum for the hydroboration of benzophenone catalysed by **3** (5 mol% Al) in C_6D_6

Benzophenone catalysed by $iBu_2Al(TMP)$, **4**

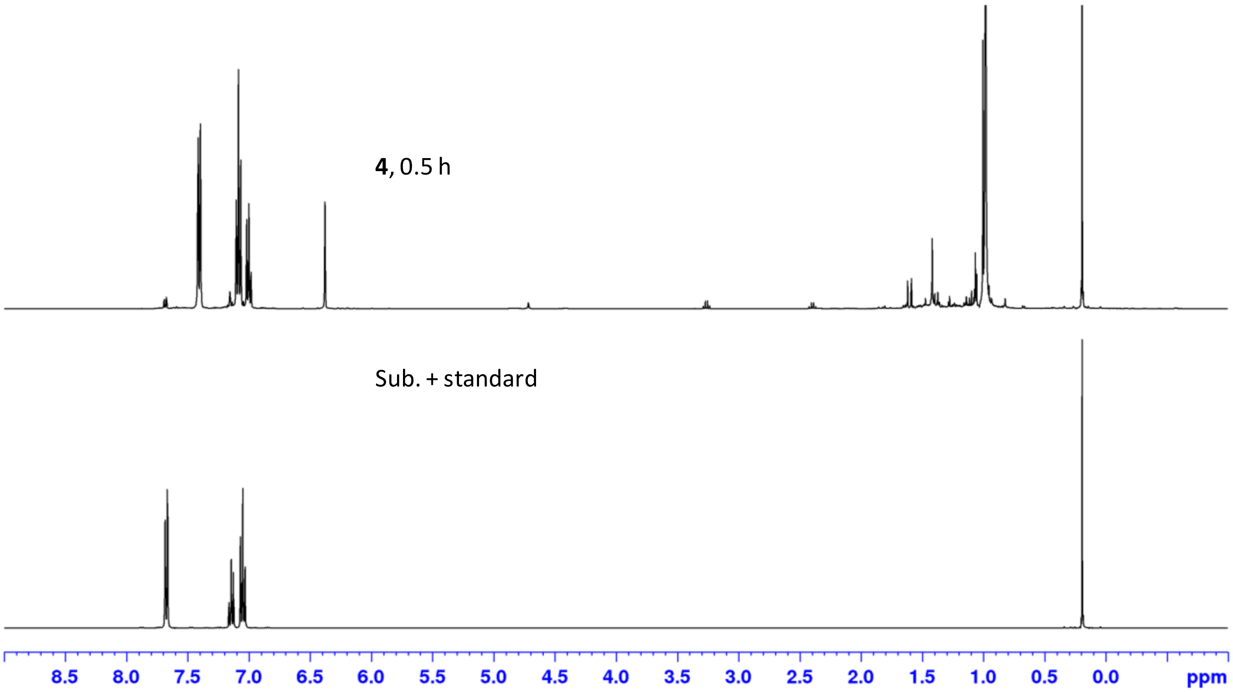

Figure S18: ^{1}H NMR spectrum for the hydroboration of benzophenone catalysed by **4** (5 mol% Al) in C_6D_6

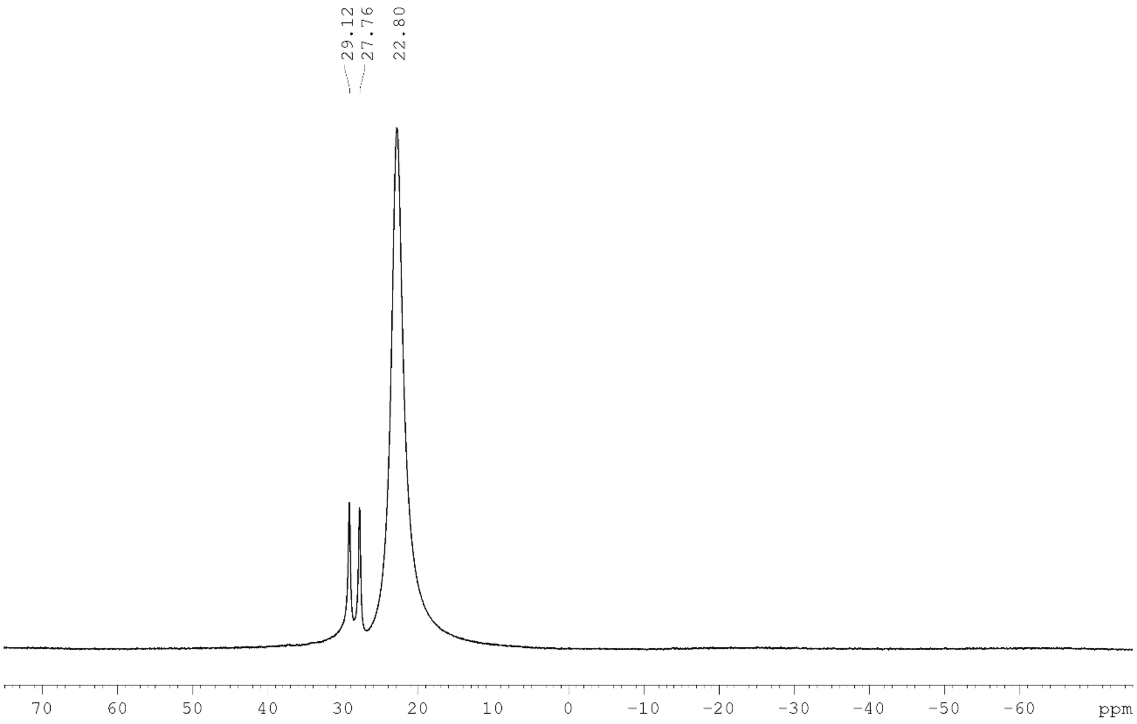

Figure S19: ^{11}B NMR spectrum for the hydroboration of benzophenone catalysed by **4** (5 mol% Al) in C_6D_6

Benzophenone catalysed by $HMDS_2AlH$, 5

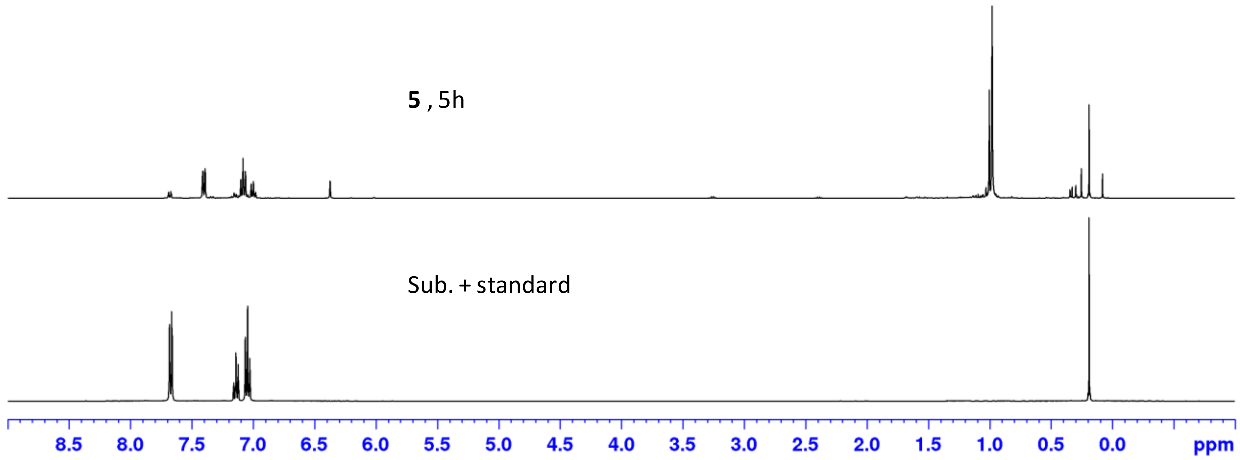

Figure S20: ^{1}H NMR spectrum for the hydroboration of benzophenone catalysed by **5** (5 mol% Al) in C_6D_6

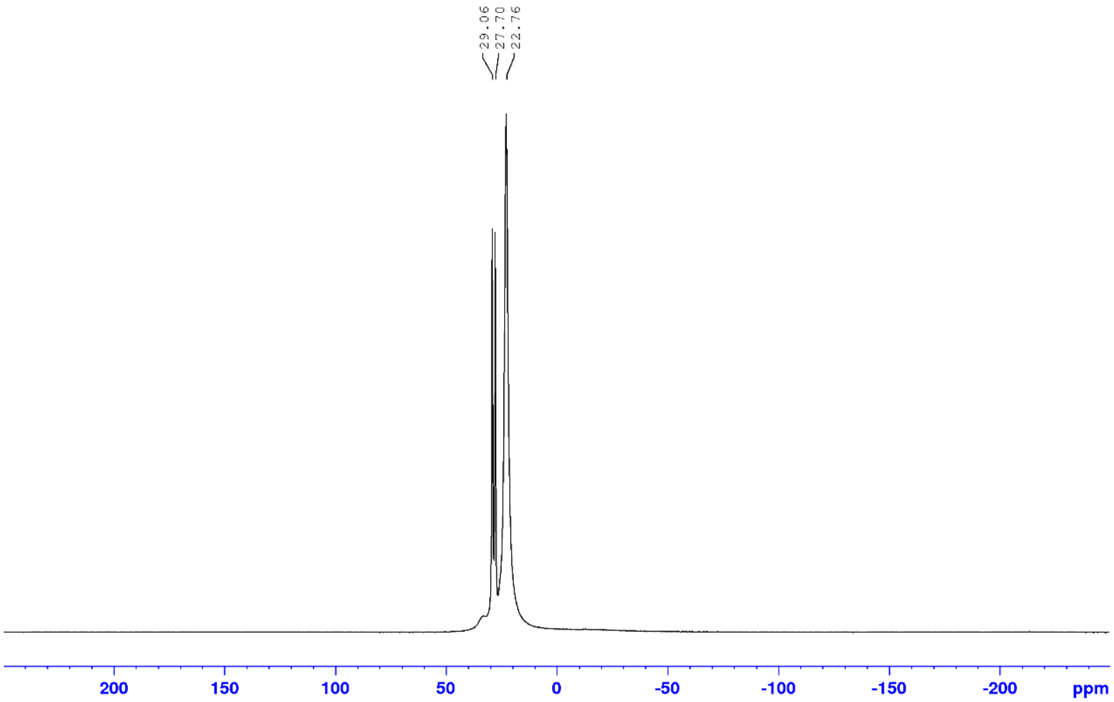

Figure S21: ^{11}B NMR spectrum for the hydroboration of benzophenone catalysed by **5** (5 mol% Al) in C_6D_6

Benzophenone catalysed by DIBAL-H, **6**

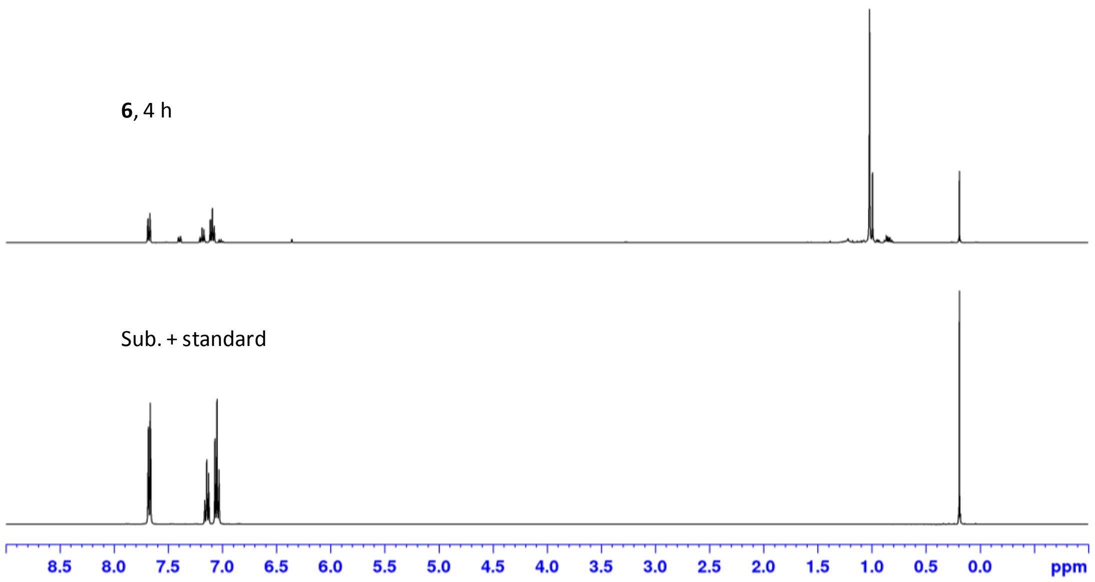

Figure S22: ^{1}H NMR spectrum for the hydroboration of benzophenone catalysed by **6** (5 mol% Al) in C_6D_6

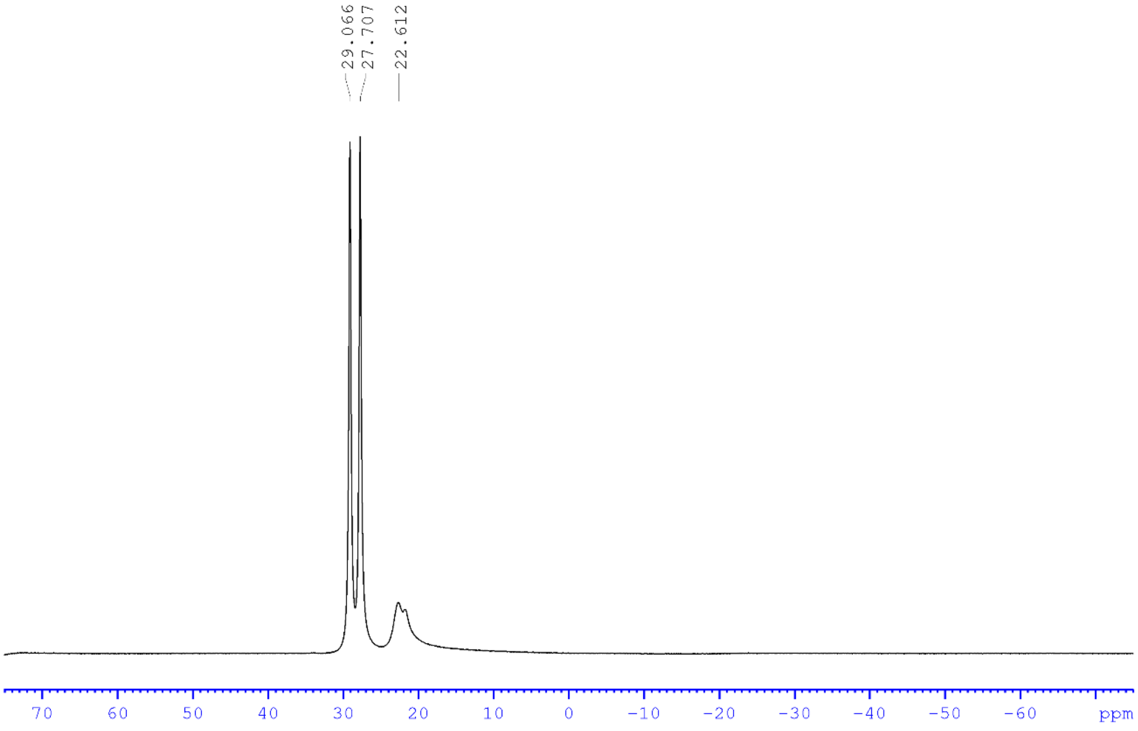

Figure S23: ^{11}B NMR spectrum for the hydroboration of benzophenone catalysed by **6** (5 mol% Al) in C_6D_6

Benzophenone catalysed by LiH

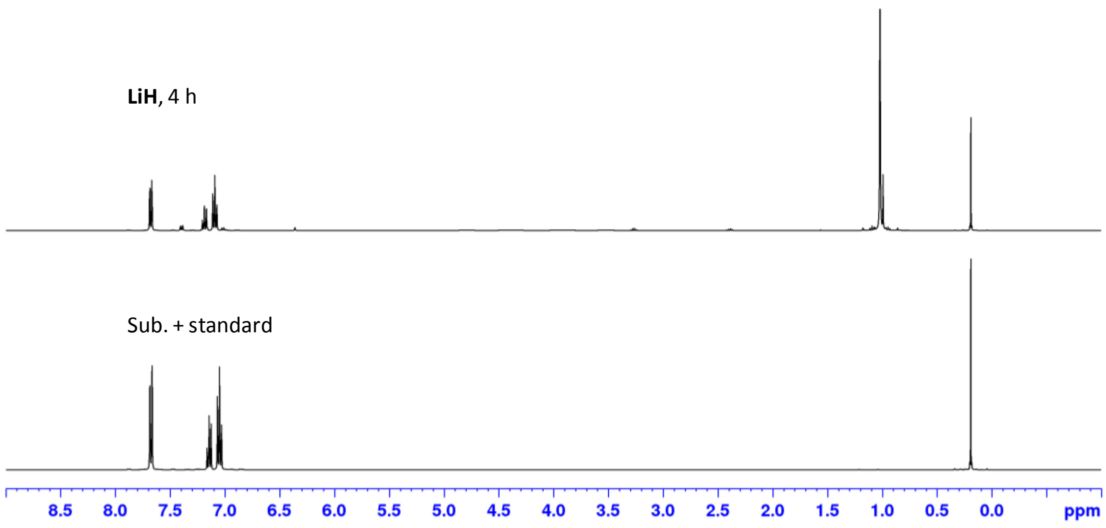

Figure S24: ^{1}H NMR spectrum for the hydroboration of acetophenone catalysed by 5 mol% LiH in C_6D_6

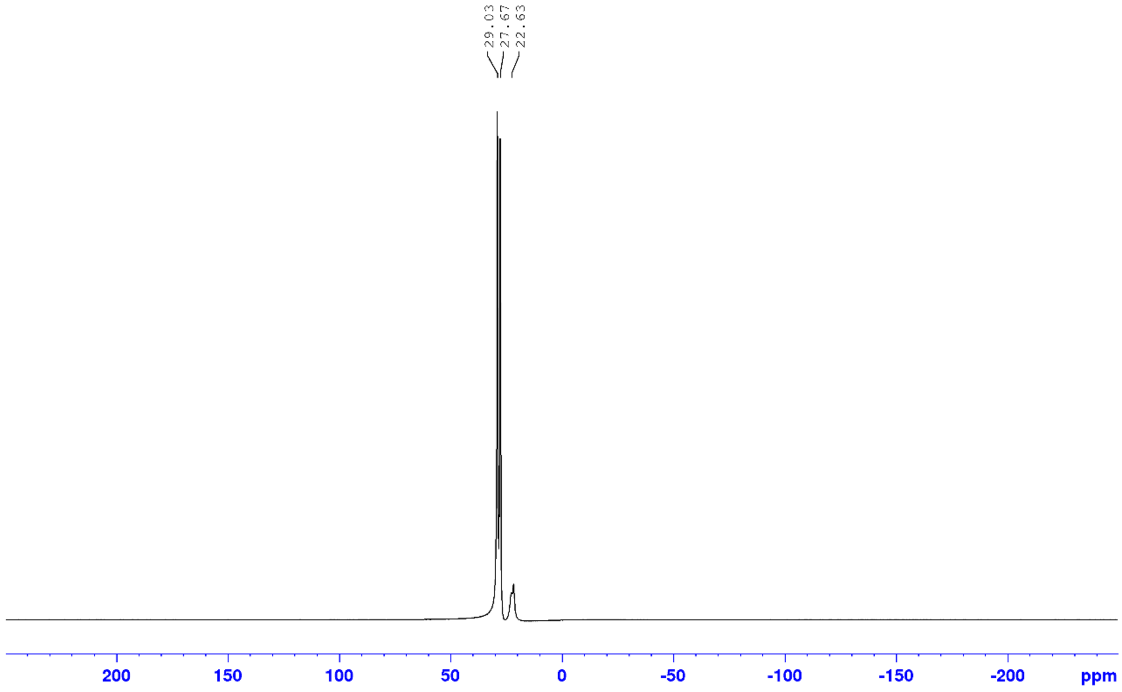

Figure S25: ^{11}B NMR spectrum for the hydroboration of benzophenone catalysed by **LiH** (5 mol%) in C_6D_6

Benzophenone catalysed by [(TMP)(Ph_2(H)CO)Al(μ-OC(H)Ph_2)]$_2$, 7

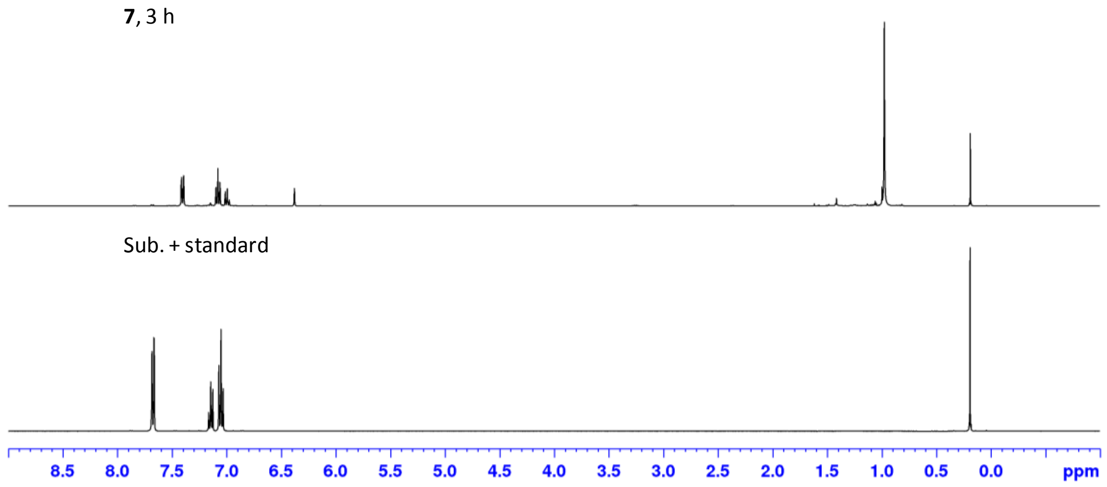

Figure S26: ^{1}H NMR spectrum for the hydroboration of acetophenone catalysed by 2.5 mol% $[(TMP)(Ph_2(H)CO)Al(\mu\text{-}OC(H)Ph_2)]_2$, **7**, in C_6D_6

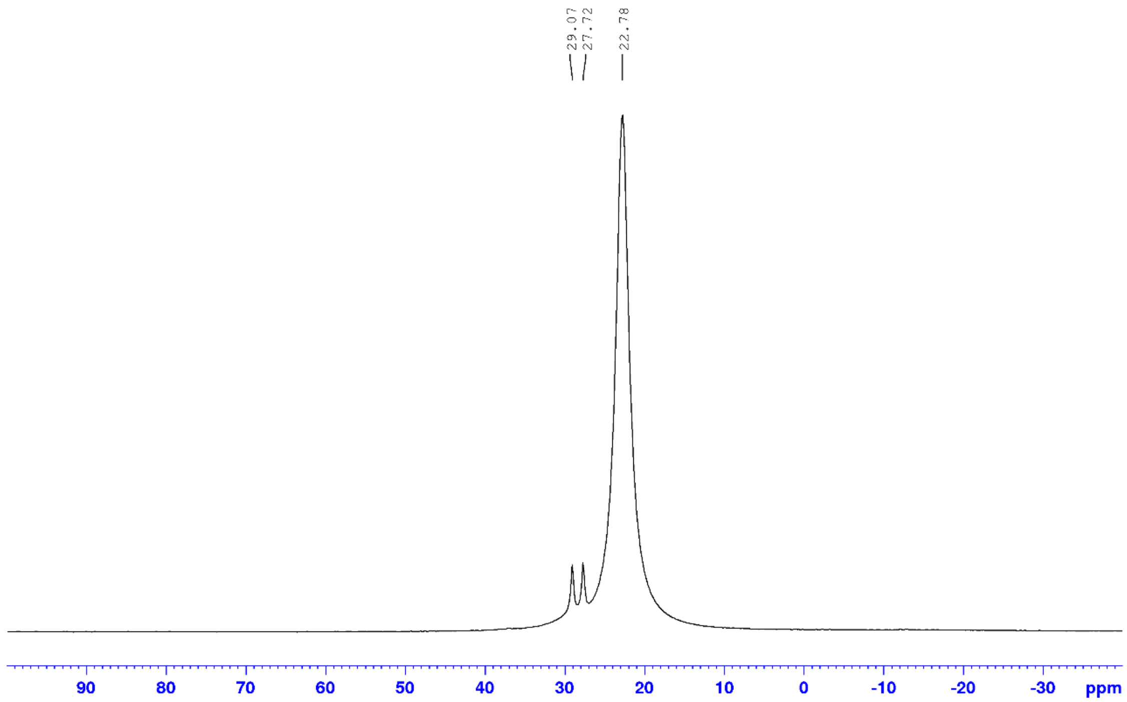

Figure S27: ^{11}B NMR spectrum for the hydroboration of acetophenone catalysed by 2.5 mol% $[(TMP)(Ph_2(H)CO)Al(\mu\text{-}OC(H)Ph_2)]_2$, **7**in C_6D_6

Acetophenone catalysed by $[iBu_3Al(H)Li]_2$, 3

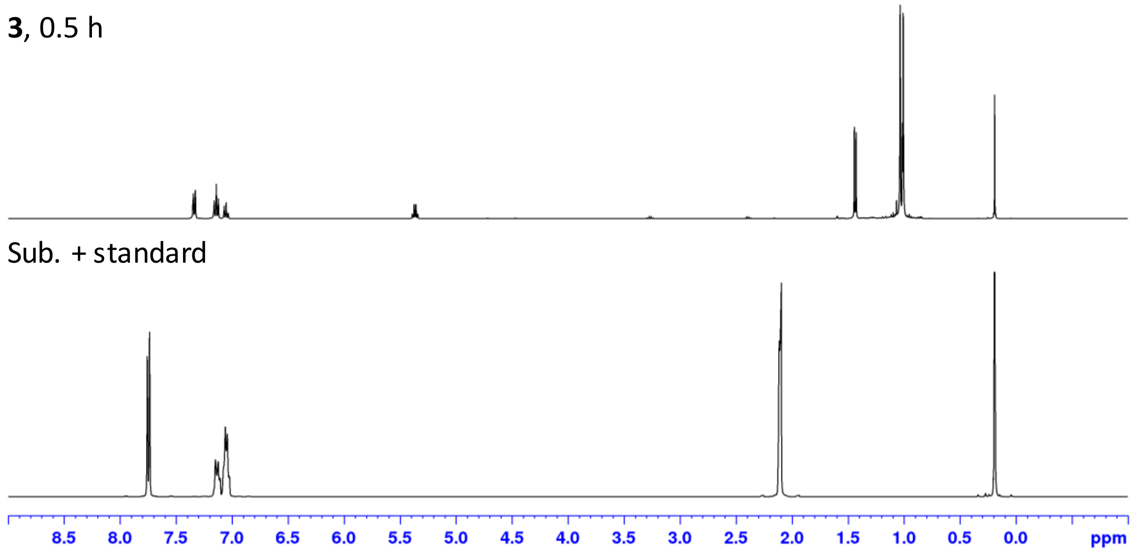

Figure S28: ^{1}H NMR spectrum for the hydroboration of acetophenone catalysed by **3** (5 mol% Al) in C_6D_6

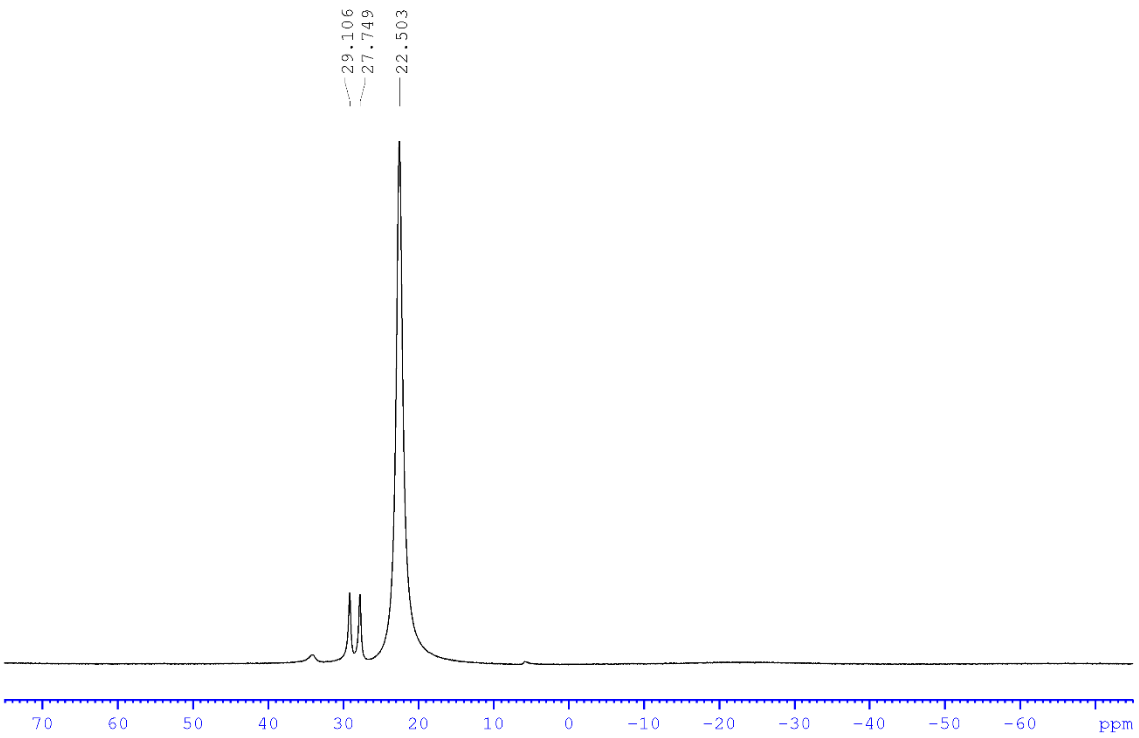

Figure S29: ^{11}B NMR spectrum for the hydroboration of acetophenone catalysed by **3** (5 mol% Al) in C_6D_6

Acetophenone catalysed by $iBu_2Al(TMP)$, **4**

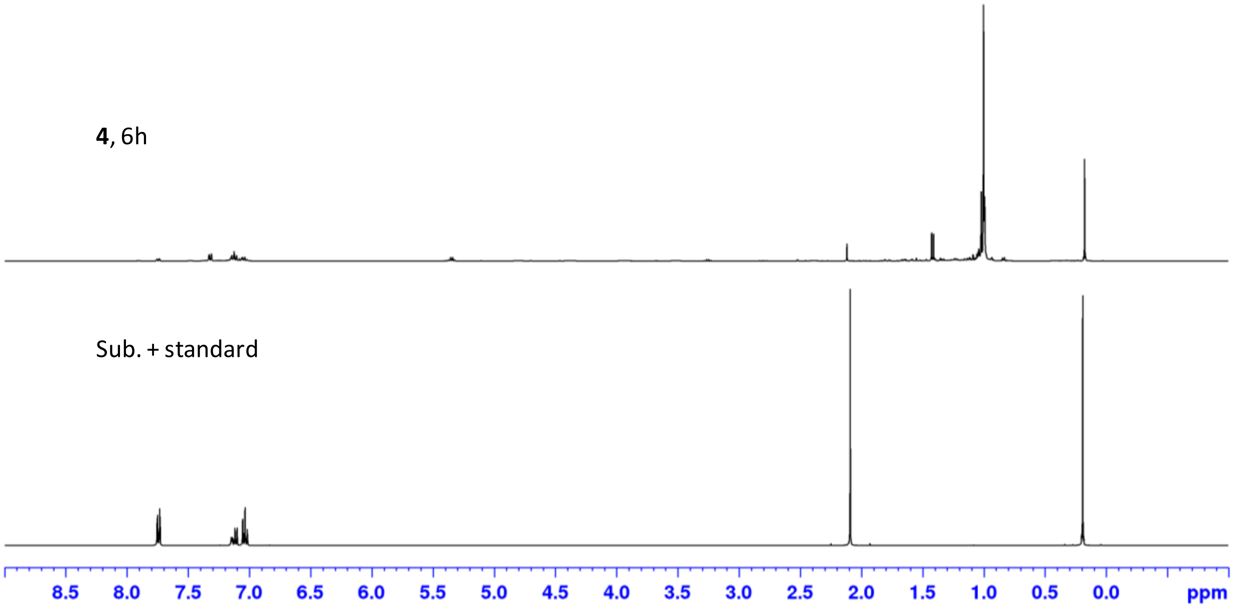

Figure S30: ^{1}H NMR spectrum for the hydroboration of acetophenone catalysed by **4** (5 mol% Al) in C_6D_6

Acetophenone catalysed by $HMDS_2AlH$, **5**

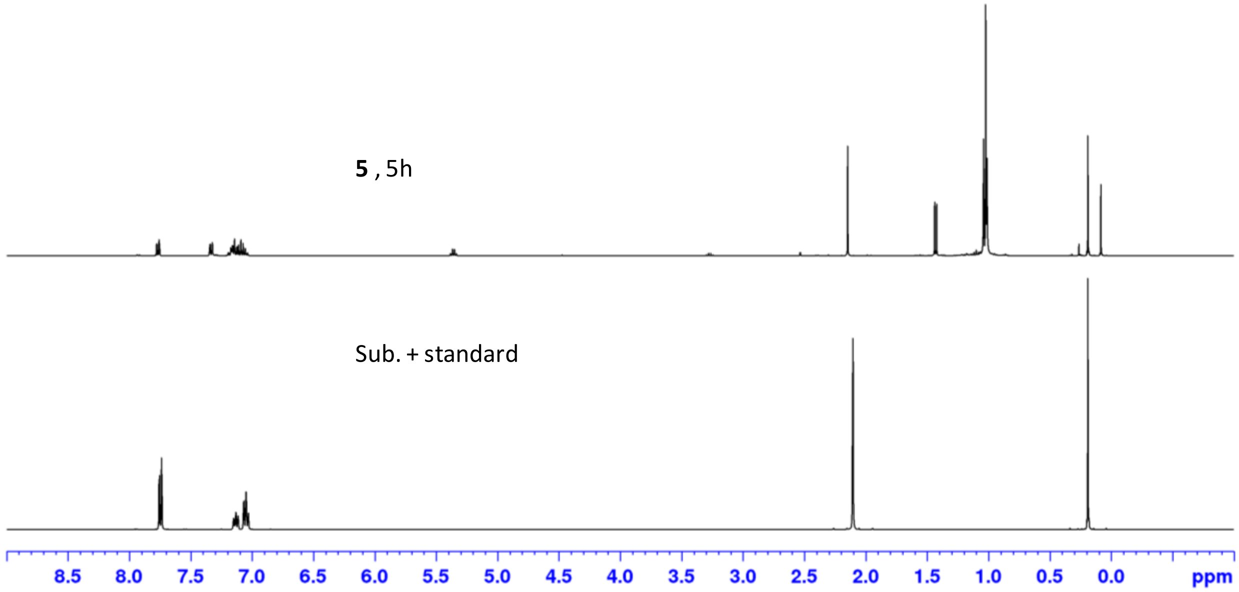

Figure S31: ^{1}H NMR spectrum for the hydroboration of acetophenone catalysed by **5** (5 mol% Al) in C_6D_6

Cyclohexanone catalysed by $[iBu_3Al(H)Li]_2$, 3

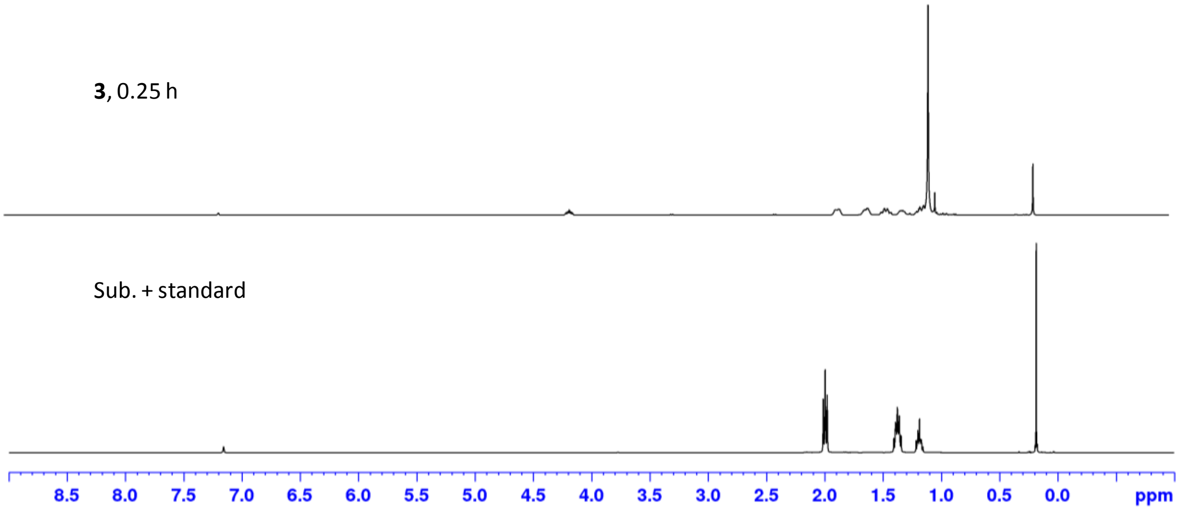

Figure S32: ^{1}H NMR spectrum for the hydroboration of cyclohexanone catalysed by **3** (5 mol% Al) in C_6D_6

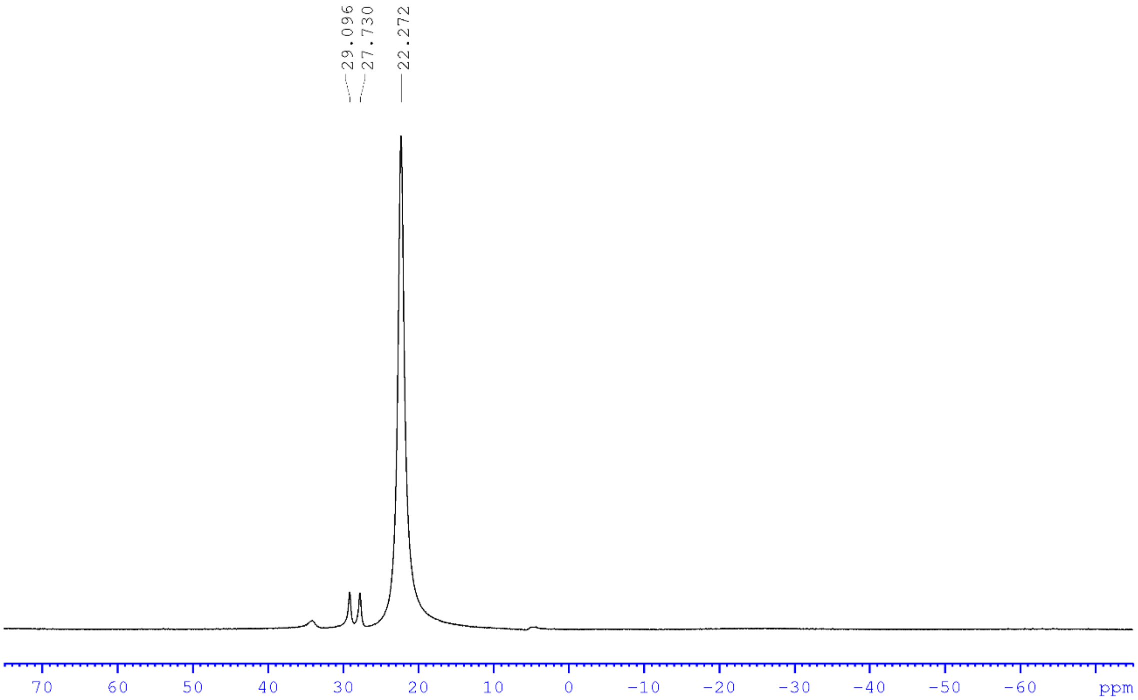

Figure S33: ^{11}B NMR spectrum for the hydroboration of cyclohexanone catalysed by **3** (5 mol% Al) in C_6D_6

Cyclohexanone catalysed by $iBu_2Al(TMP)$, 4

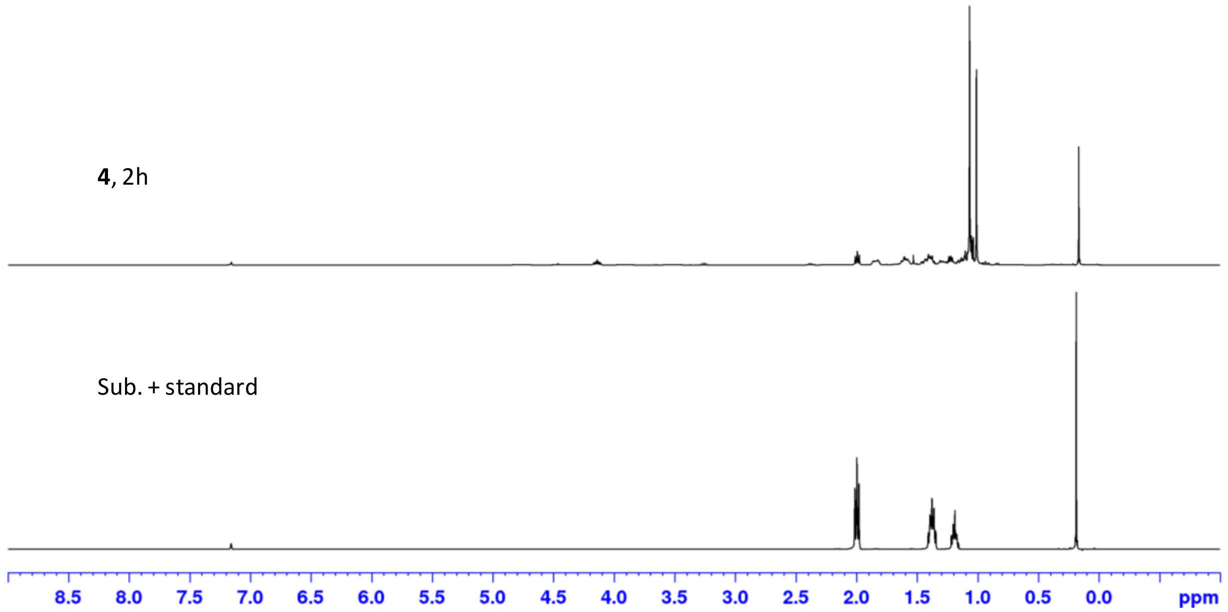

Figure S34: ^{1}H NMR spectrum for the hydroboration of cyclohexanone catalysed by **4** (5 mol% Al) in C_6D_6

Cyclohexanone catalysed by $HMDS_2AlH$, 5

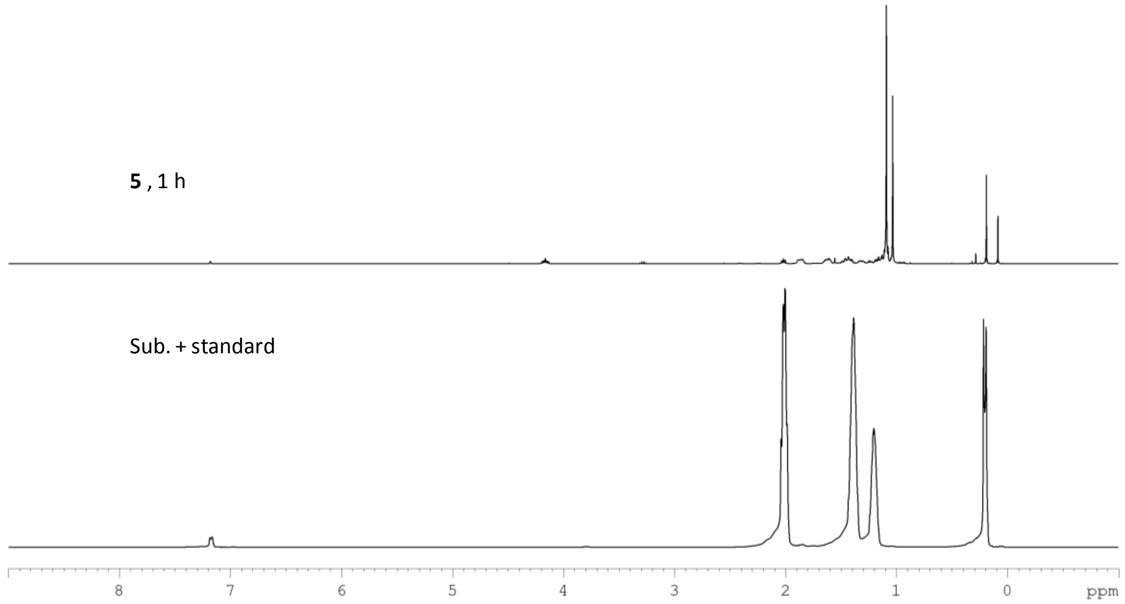

Figure S35: ^{1}H NMR spectrum for the hydroboration of cyclohexanone catalysed by **5** (5 mol% Al) in C_6D_6

Benzaldehyde catalysed by $[iBu_3Al(H)Li]_2$, 3

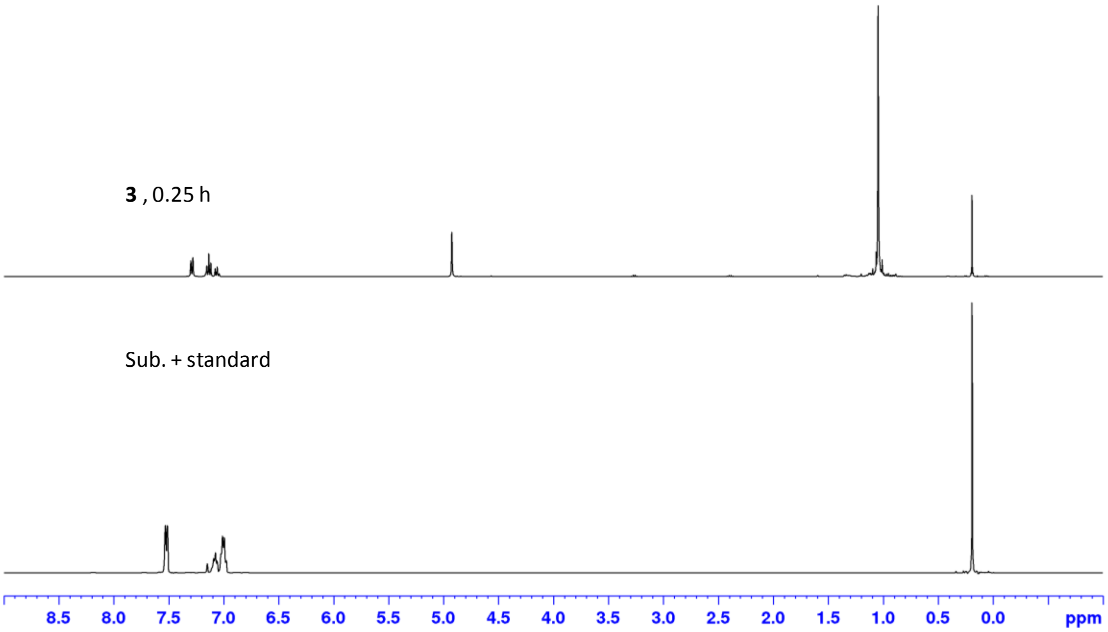

Figure S36: ^{1}H NMR spectrum for the hydroboration of benzaldehyde catalysed by **3** (5 mol% Al) in C_6D_6

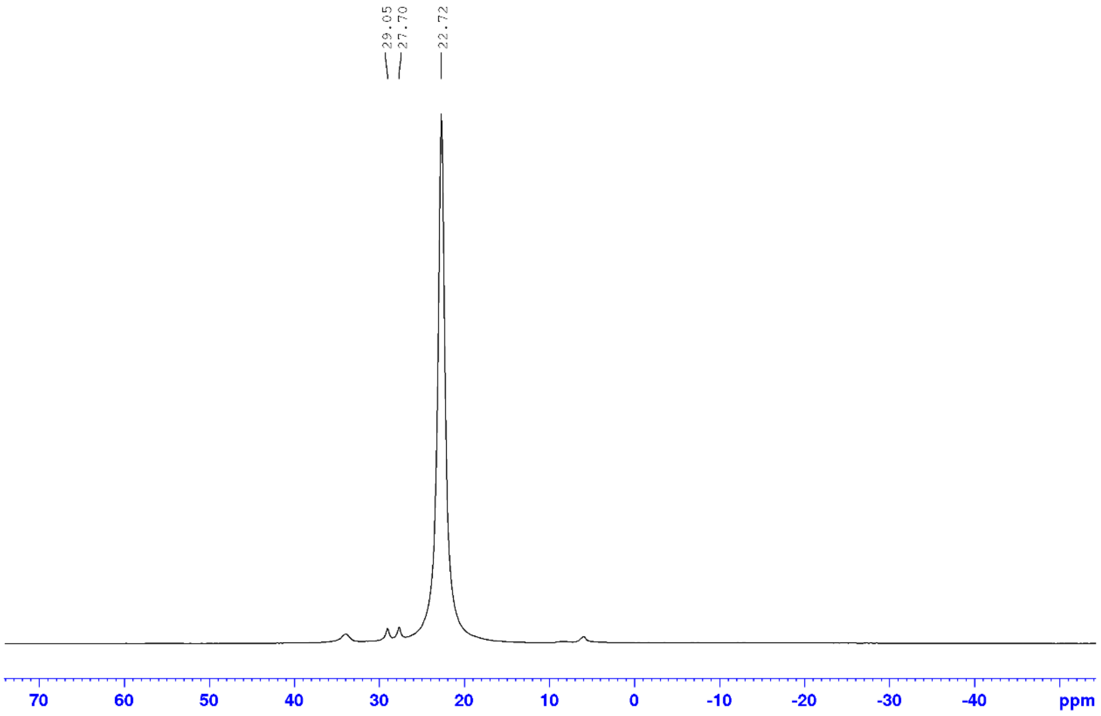

Figure S37: ^{11}B NMR spectrum for the hydroboration of benzaldehyde catalysed by **3** (5 mol% Al) in C_6D_6

Benzaldehyde catalysed by iBu$_2$Al(TMP), **4**

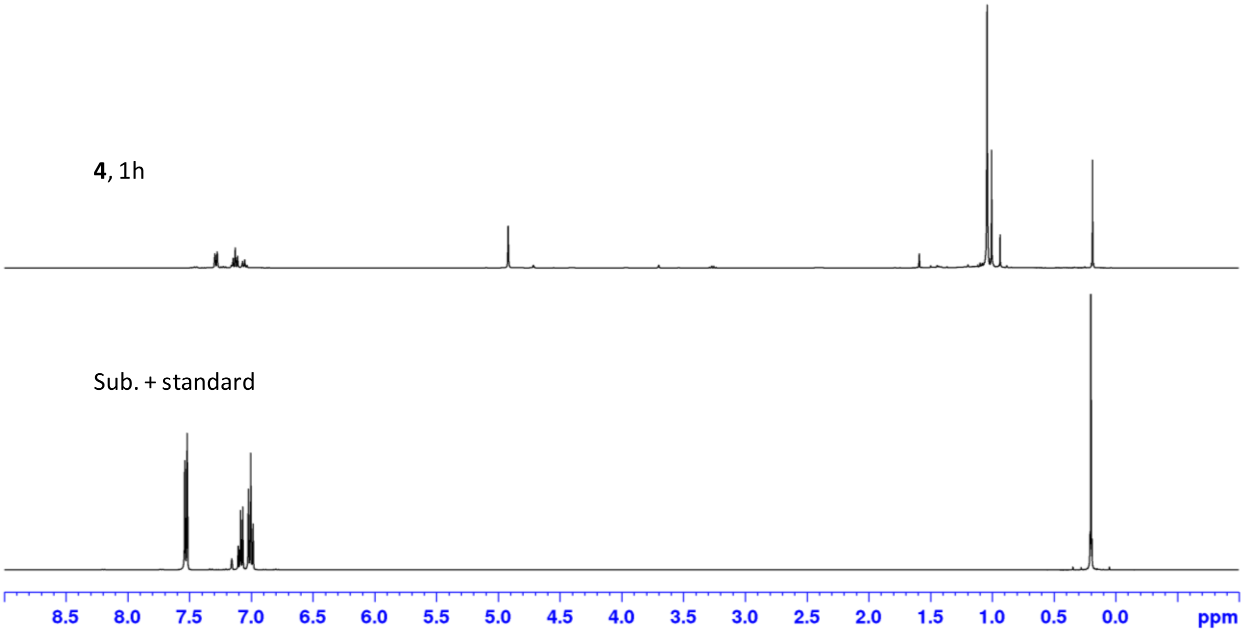

Figure S38: ^{1}H NMR spectrum for the hydroboration of benzaldehyde catalysed by **4** (5 mol% Al) in C_6D_6

Benzaldehyde catalysed by HMDS$_2$AlH, **5**

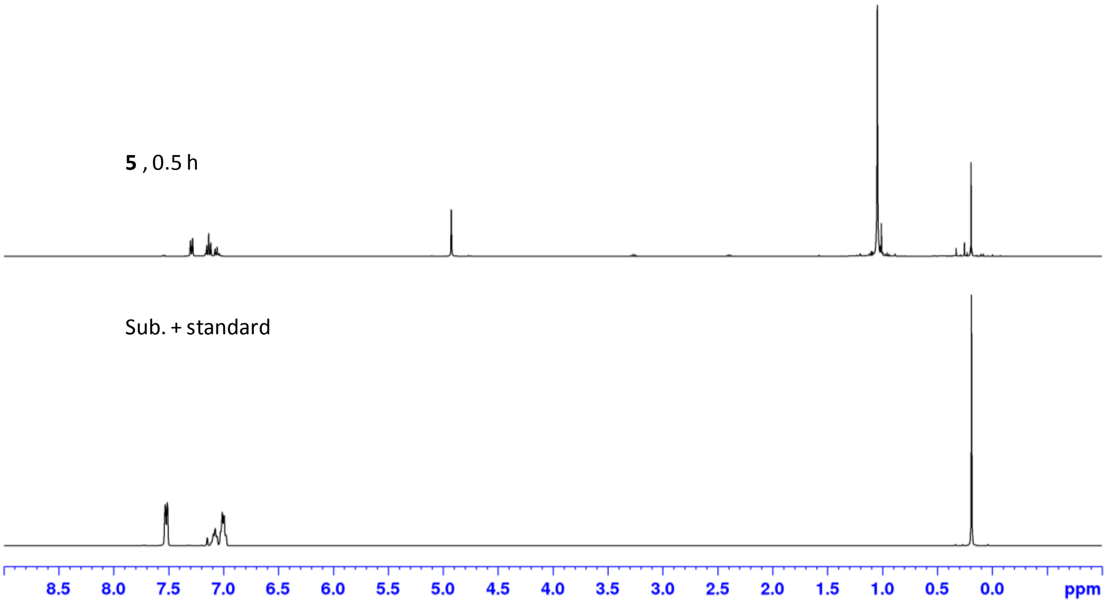

Figure S39: ^{1}H NMR spectrum for the hydroboration of benzaldehyde catalysed by **5** (5 mol% Al) in C_6D_6

Stoichiometric Reactions of Imines:

N-benzylidenemethylamine + [iBu$_2$Al(TMP)(H)Li]$_2$, 1

In a J. Young's NMR tube [iBu$_2$Al(TMP)(H)Li]$_2$ (0.036 g 0.0625 mmol) was dissolved in C_6D_6 and N-benzylidenemethylamine (0.015 mL; 0.125 mmol) added.

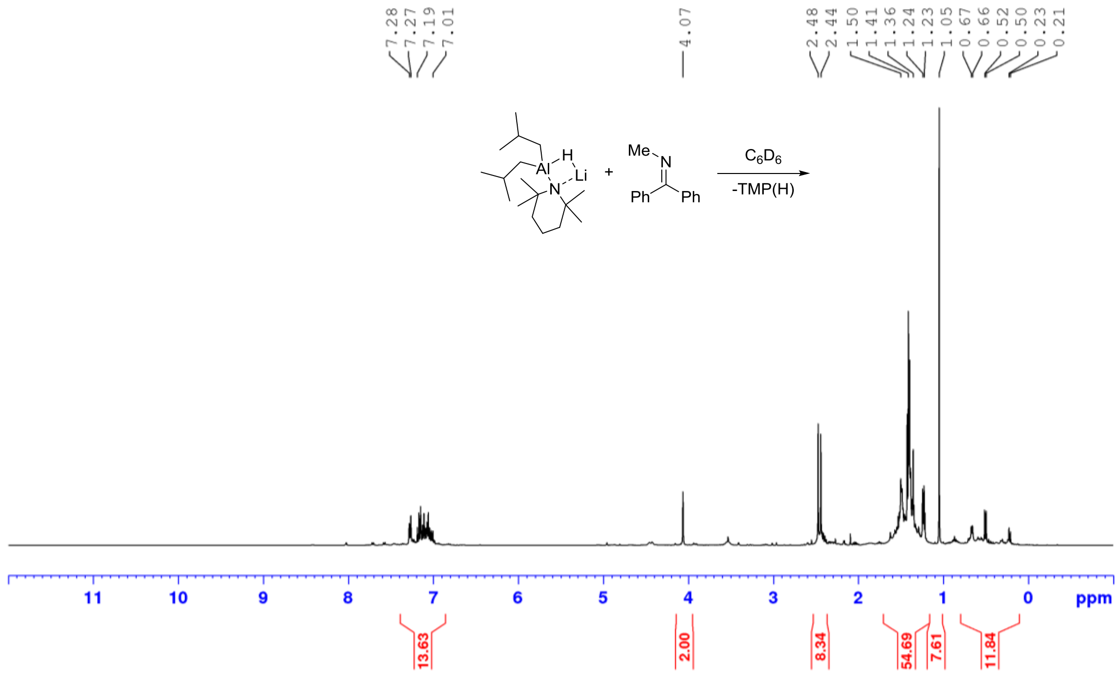

Figure S40: J Young's NMR reaction between N-benzylidenemethylamine + iBu$_2$Al(TMP)(H)Li, **1**, in C_6D_6, showing hydroalumination.

N-benzylidenemethylamine + [iBu$_3$Al(H)Li]$_2$, 3

In a J. Young's NMR tube[iBu$_2$Al(H)Li]$_2$ (0.026 g; 0.0625 mmol) was dissolved in C_6D_6 (0.5 mL) and N-N-benzylidenemethylamine (0.015 mL; 0.125 mmol) was added.

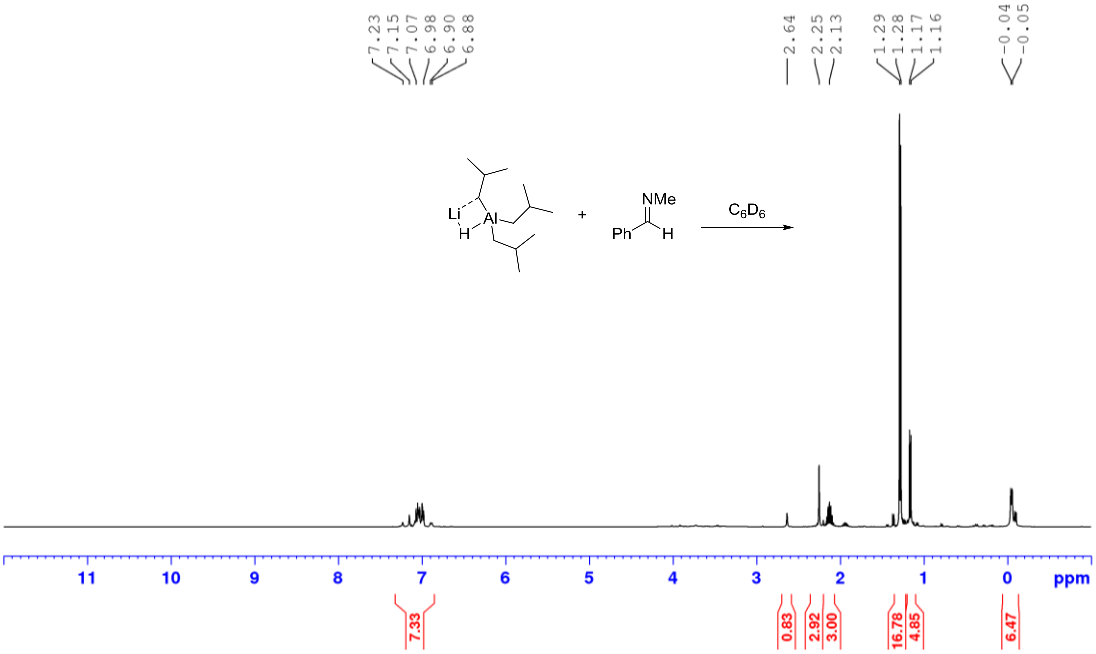

Figure S41: J Young's NMR reaction between N-benzylidenemethylamine + iBu$_3$Al(H)Li, **3**, in C_6D_6, showing hydroalumination.

N-benzylidenemethylamine + iBu$_2$Al(TMP), **4**

In a J. Young's NMR tube iBu$_2$Al(TMP) (0.070 g; 0.25 mmol) was dissolved in C_6D_6 and then N-benzylidenemethylamine (0.031 mL; 0.25 mmol) was added.

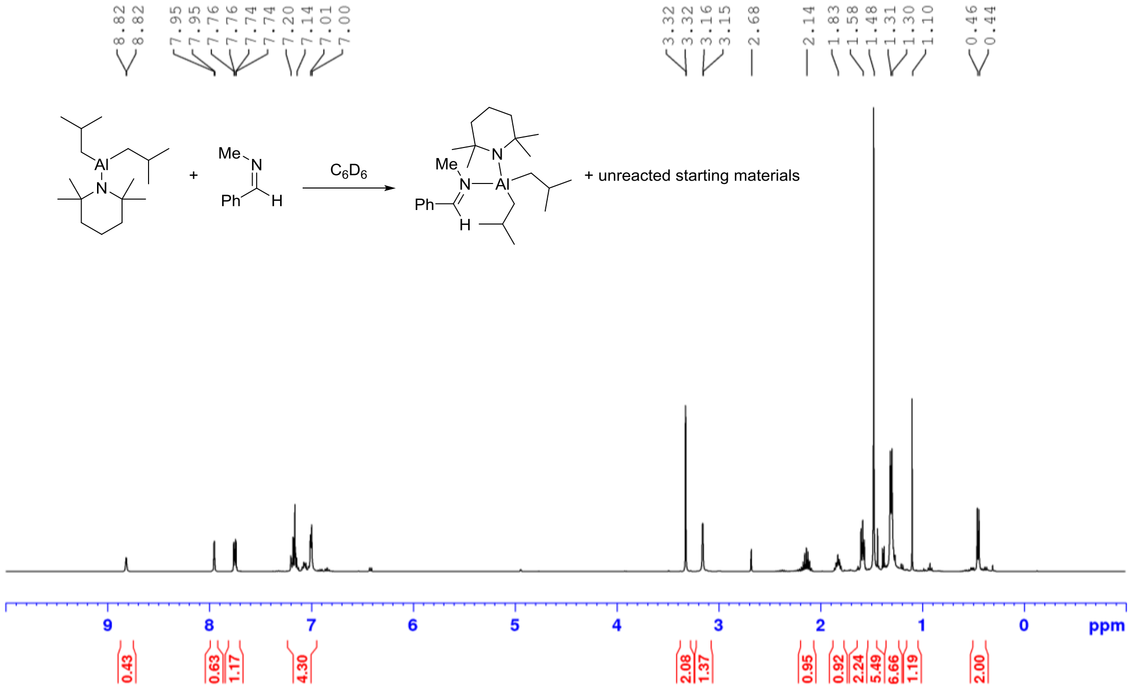

Figure S42: J Young's NMR reaction between N-benzylidenemethylamine + iBu$_2$Al(TMP), **4**, in C_6D_6, showing coordination

N-benzylidenemethylamine + DIBAL, 6

In a J. Young's NMR tube DIBAL (0.1422 g; 1 mmol) and N-benzylidenemethylamine (0.12 mL; 1 mmol) were reacted in C_6D_6 (0.5 mL) solution.

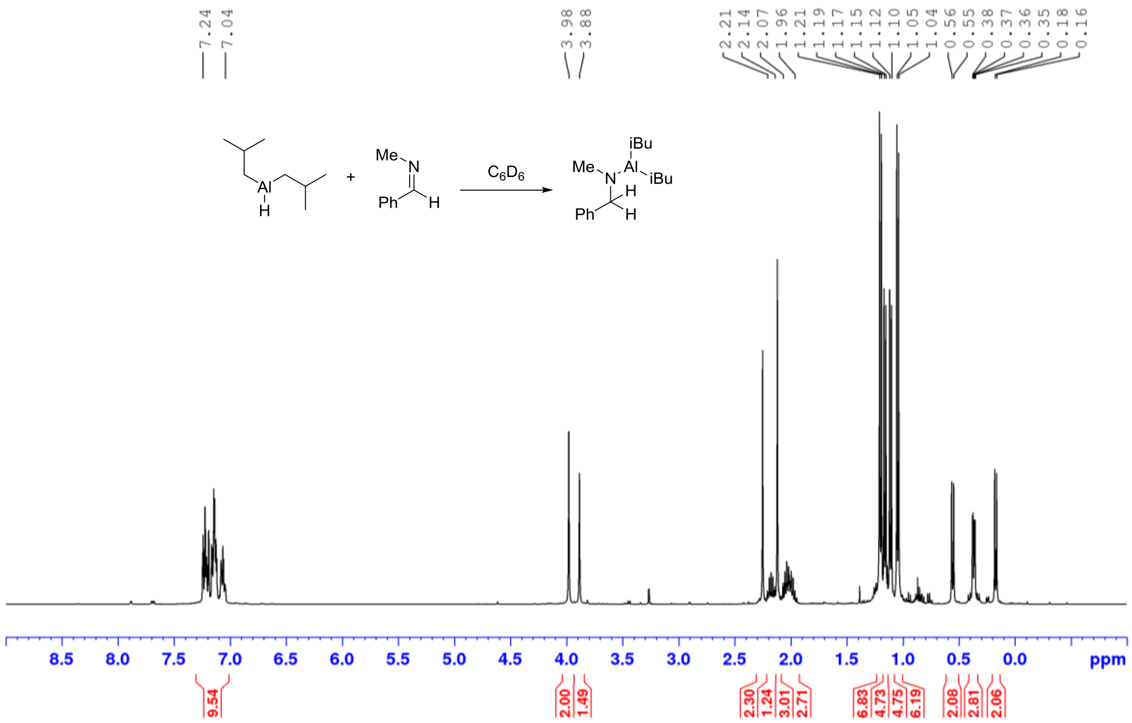

Figure S43: J Young's NMR reaction between N-benzylidenemethylamine + DIBAL(H), **6**, in C_6D_6, showing hydroalumination

Benzophenone imine + [iBu$_2$Al(TMP)(H)Li]$_2$, 1

In a J. Young's NMR tube [iBu$_2$Al(TMP)(H)Li]$_2$ (0.125 mmol; 0.058 g) was dissolved in C_6D_6 (0.5 mL) and benzophenone imine (0.04 mL; 0.25 mmol) added.

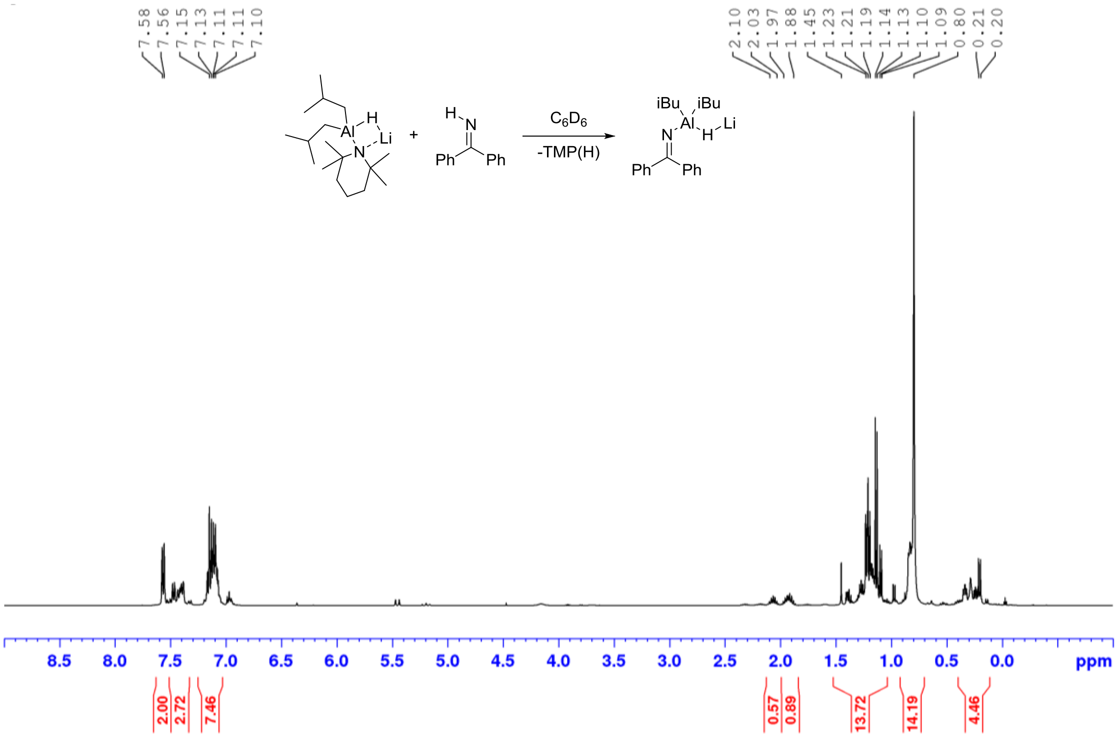

Figure S44: J Young's NMR reaction between benzophenone imine + iBu$_2$Al(TMP)(H)Li, **1**, in C_6D_6, showing deprotonation

Benzophenone imine + $[iBu_3Al(H)Li]_2$, **3**

In a J. Young's NMR tube $[iBu_2Al(H)Li]_2$ (0.026 g; 0.0625 mmol) was dissolved in C_6D_6 (0.5 mL) solution and benzophenone imine (0.021 mL; 0.125 mmol) added.

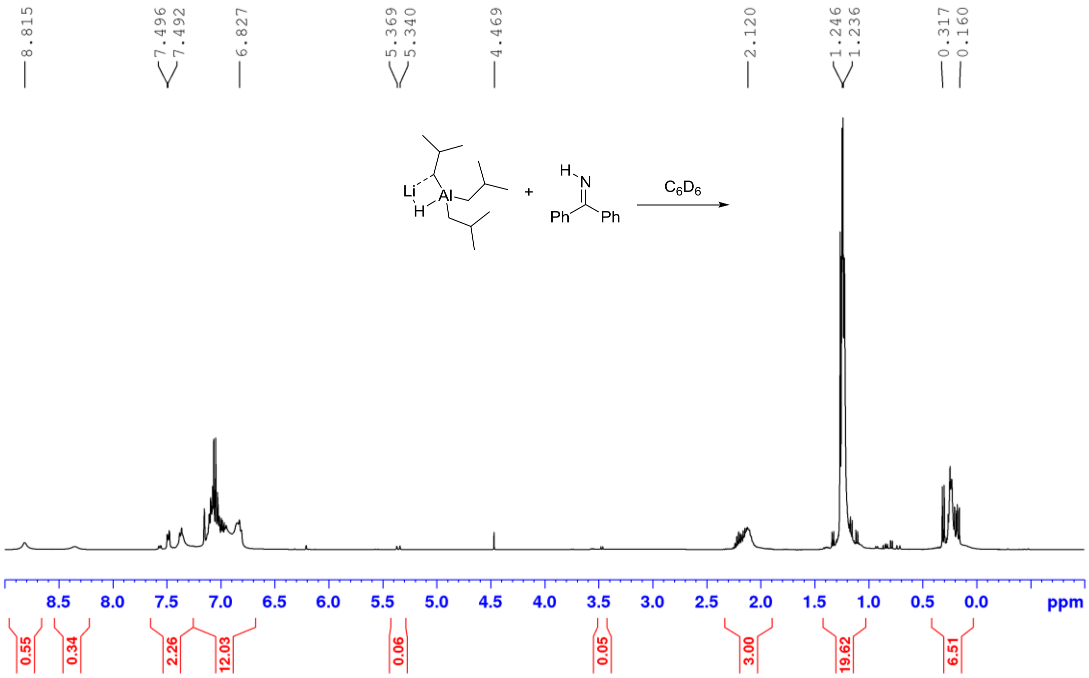

Figure S45: J Young's NMR reaction between benzophenone imine + $[iBu_3Al(H)Li]_2$, **3**, in C_6D_6, showing slow hydroalumination, and some trace H_2 evolution

Benzophenone imine + DIBAL(H), **6**

In a J. Young's NMR tube DIBAL(H) (0.0711 g; 0.5 mmol) in C_6D_6 (0.5 mL) was added to benzophenone imine (0.08 mL; 0.5 mmol).

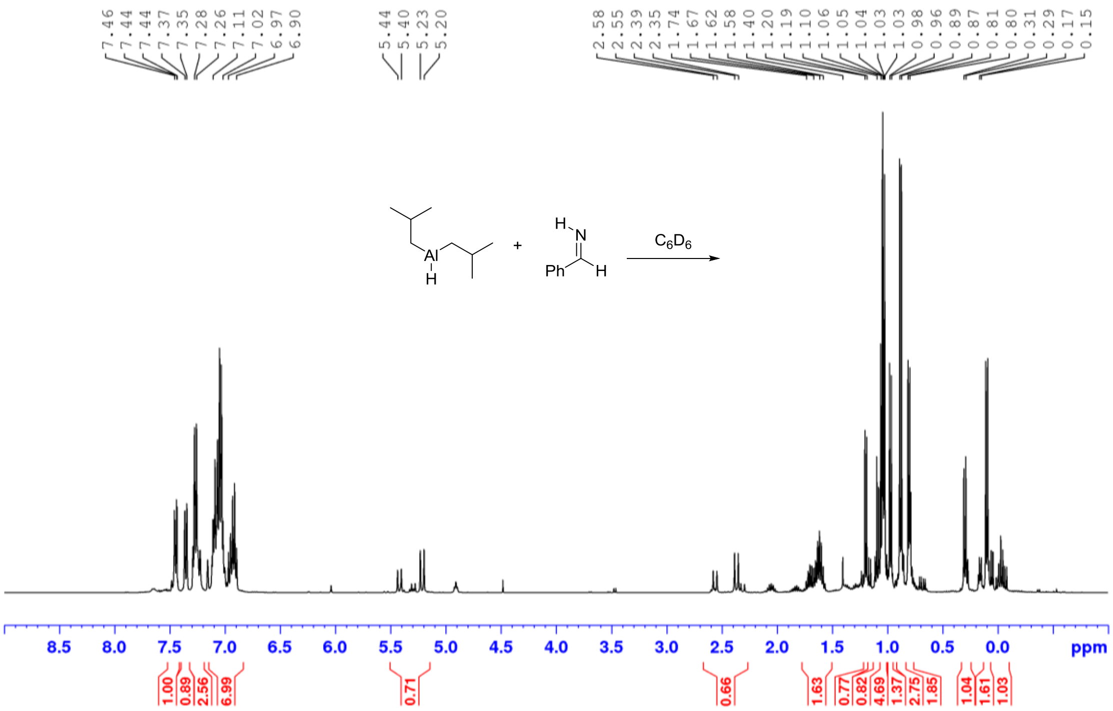

Figure S46: J Young's NMR reaction between benzophenone imine + DIBAL(H), **6**, in C_6D_6, showing hydroalumination of the imine.

Hydroboration of Imines

Imine hydroboration reactions were undertaken at 0.5 mmol scale, at room temperature using 10 mol% [Al] catalyst **1** – **6** in C_6D_6 solution.

N-benzylidenemethylamine catalysed by $[iBu_2Al(TMP)(H)Li]_2$, **1**

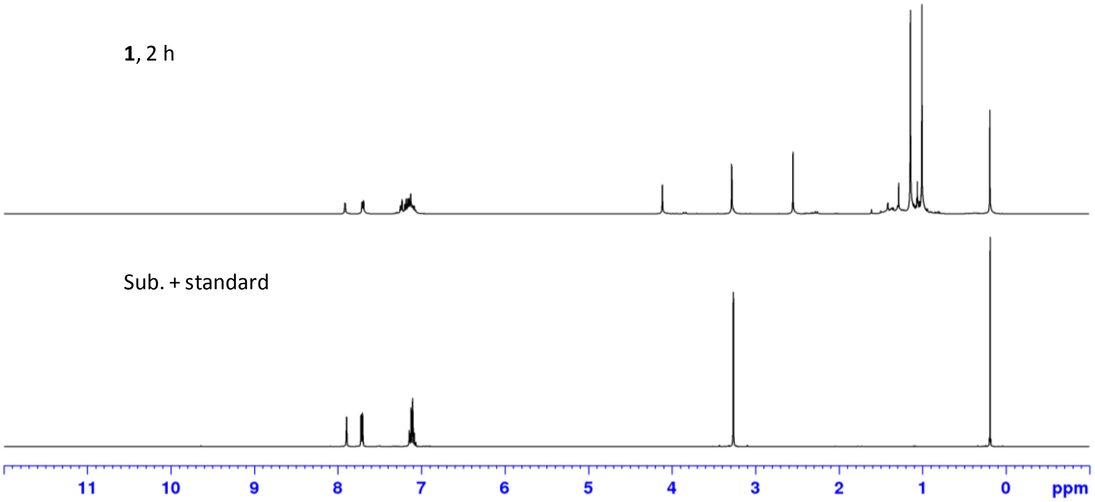

Figure S47 ^{1}H NMR spectrum for the hydroboration of N-benzylidenemethylamine catalysed by **1** (10 mol% Al) in C_6D_6

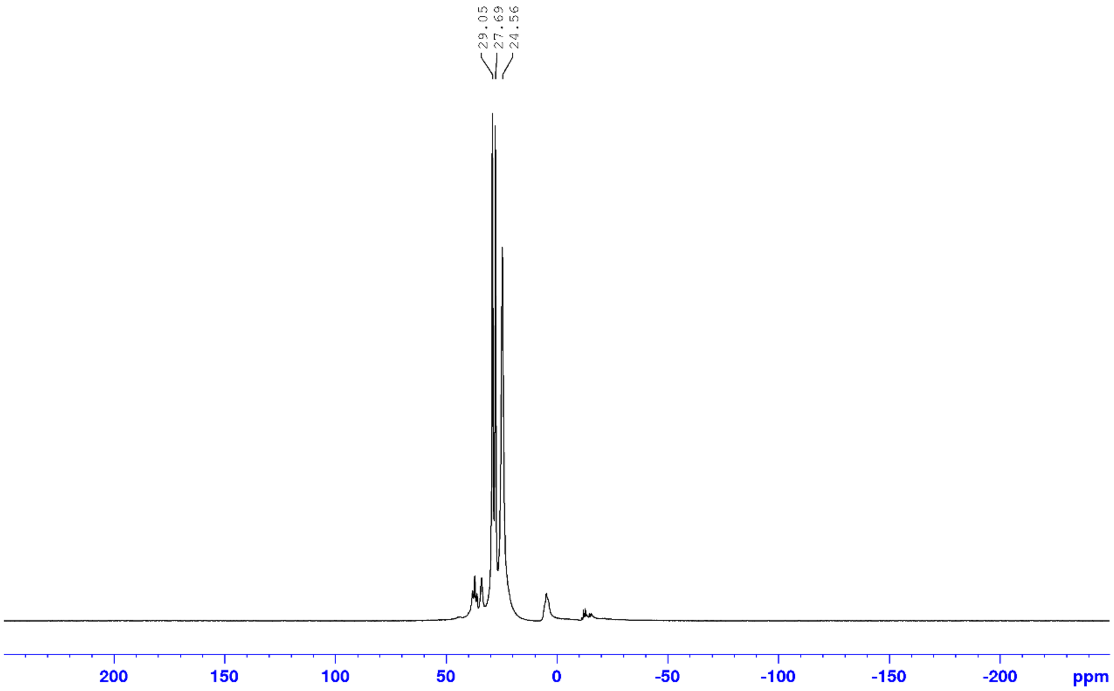

Figure S48: ^{11}B NMR spectrum for the hydroboration of N-benzylidenemethylamine catalysed by **1** (10 mol% Al) in C_6D_6

N-benzylidenemethylamine catalysed by $(HMDS)_2AlH(\mu\text{-}H)Li(THF)_3$, 2

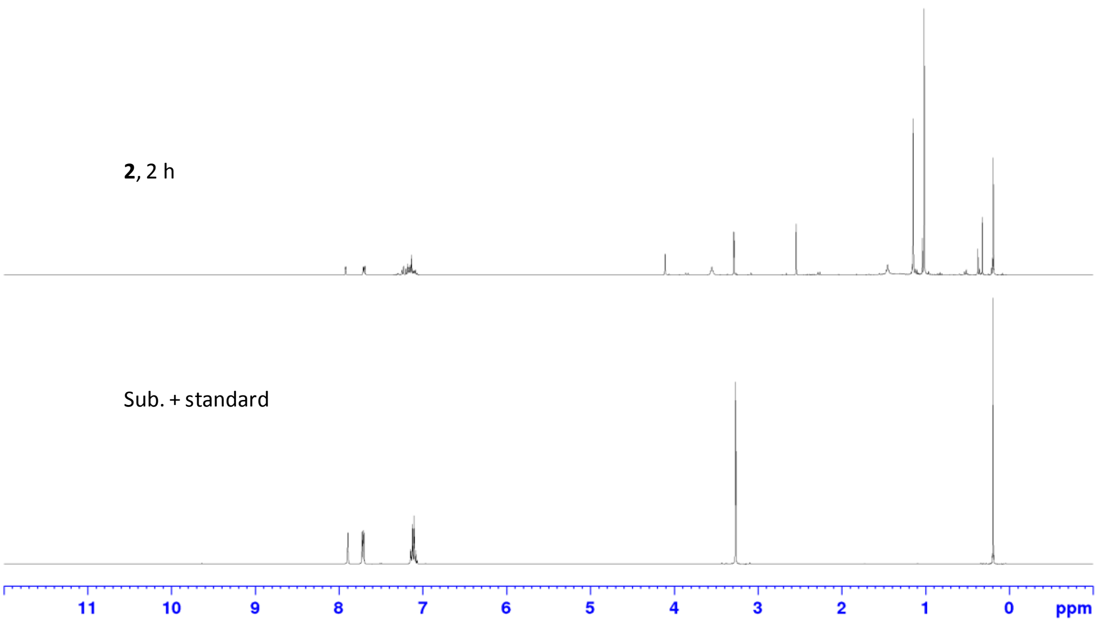

Figure S49: ^1H NMR spectrum for the hydroboration of N-benzylidenemethylamine catalysed by **2** (10 mol% Al) in C_6D_6

N-benzylidenemethylamine catalysed by $[iBu_3Al(H)Li]_2$, 3

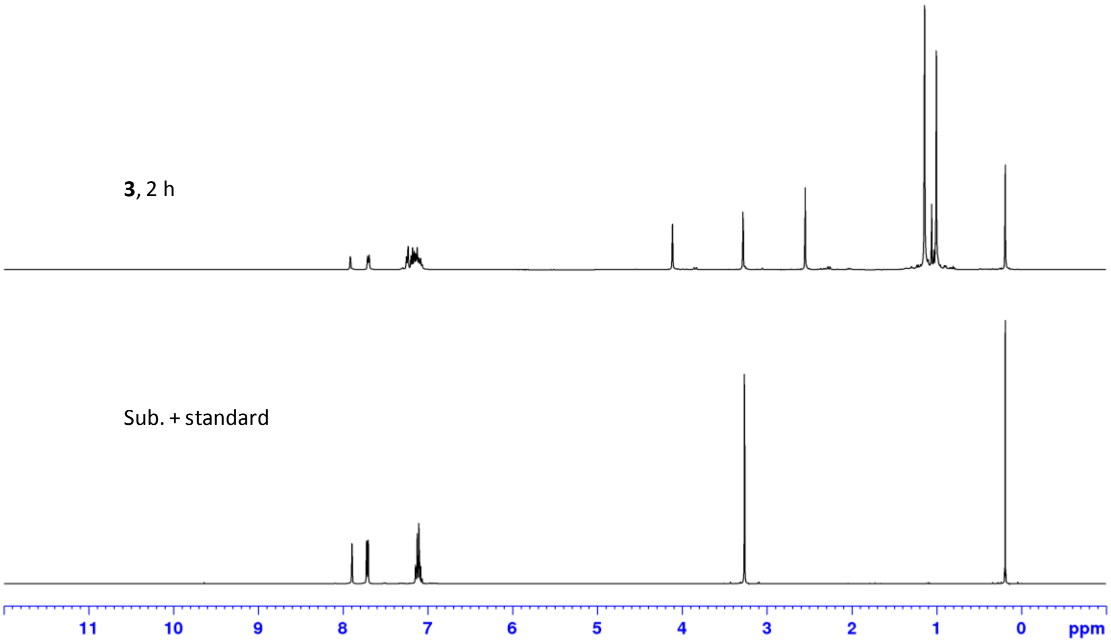

Figure S50: ^1H NMR spectrum for the hydroboration of N-benzylidenemethylamine catalysed by **3** (10 mol% Al) in C_6D_6

N-benzylidenemethylamine catalysed by $iBu_2Al(TMP)$, 4

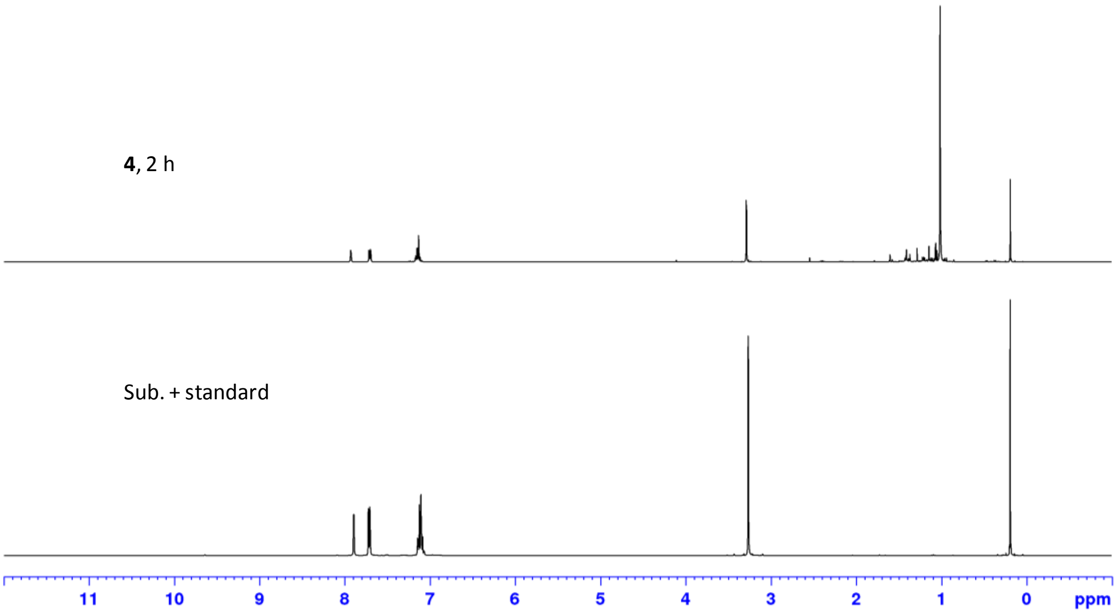

Figure S51: ^{1}H NMR spectrum for the hydroboration of N-benzylidenemethylamine catalysed by **4** (10 mol% Al) in C_6D_6

N-benzylidenemethylamine catalysed by $HMDS_2AlH$, 5

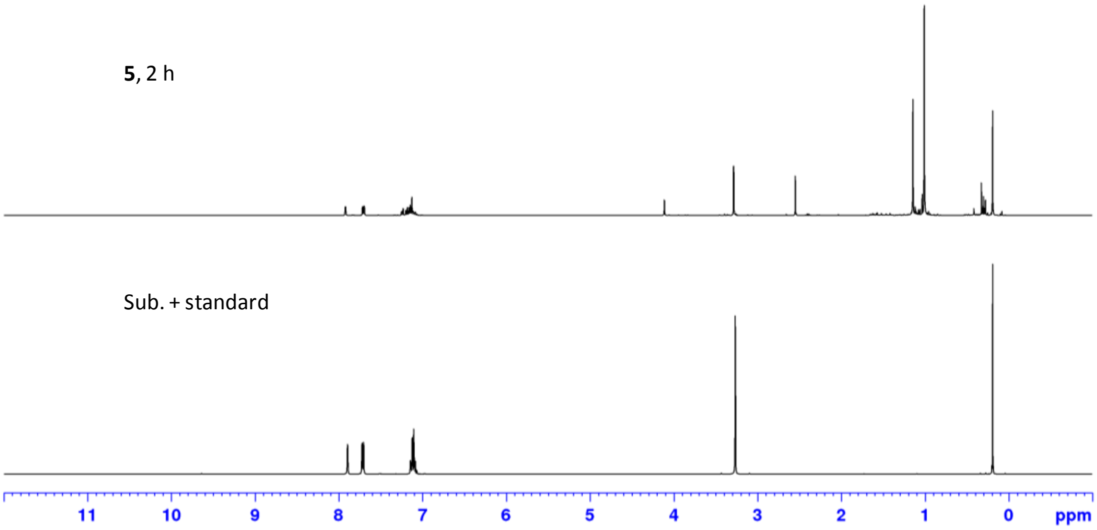

Figure S52: ^{1}H NMR spectrum for the hydroboration of N-benzylidenemethylamine catalysed by **5** (10 mol% Al) in C_6D_6

N-benzylidenemethylamine catalysed by DIBAL(H), **6**

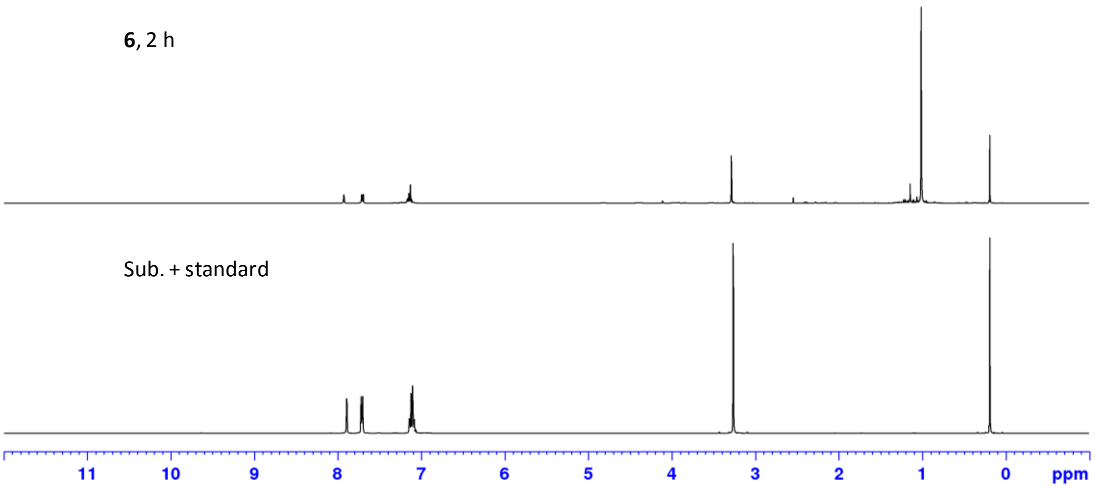

Figure S53: ^{1}H NMR spectrum for the hydroboration of N-benzylidenemethylamine catalysed by **6** (10 mol% Al) in C_6D_6

Benzophenone imine catalysed by $[iBu_2Al(TMP)(H)Li]_2$, **1**

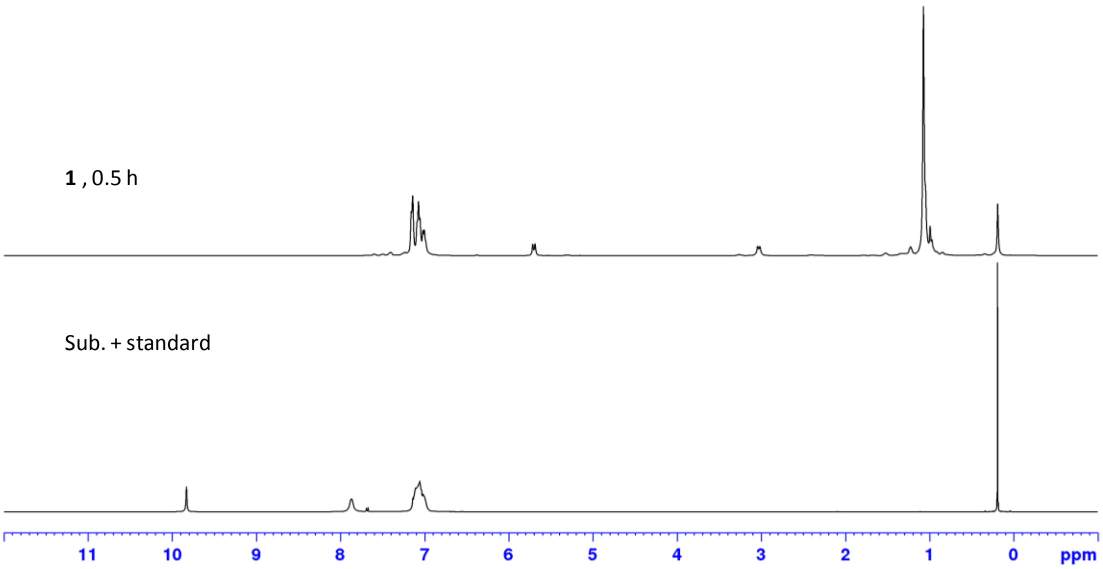

Figure S54: ^{1}H NMR spectrum for the hydroboration of benzophenone imine catalysed by **1** (10 mol% Al) in C_6D_6

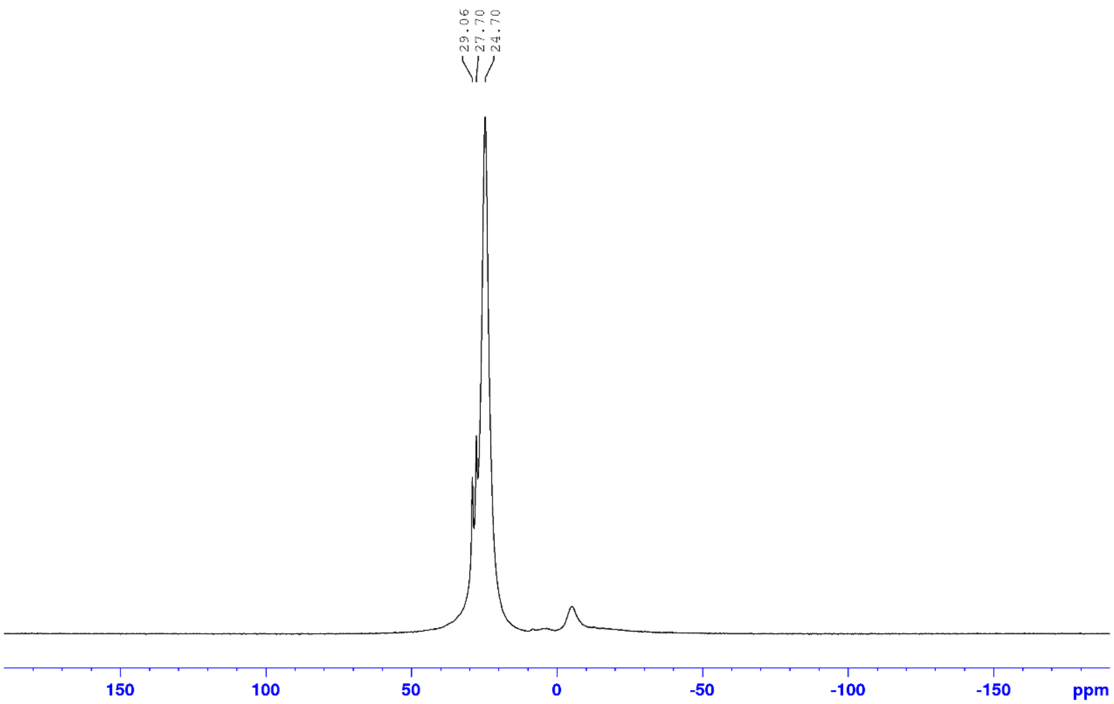

Figure S55: ^{11}B NMR spectrum for the hydroboration of benzophenone imine catalysed by **1** (10 mol% Al) in C_6D_6

Benzophenone imine catalysed by $(HMDS)_2AlH(\mu\text{-}H)Li(THF)_3$, 2

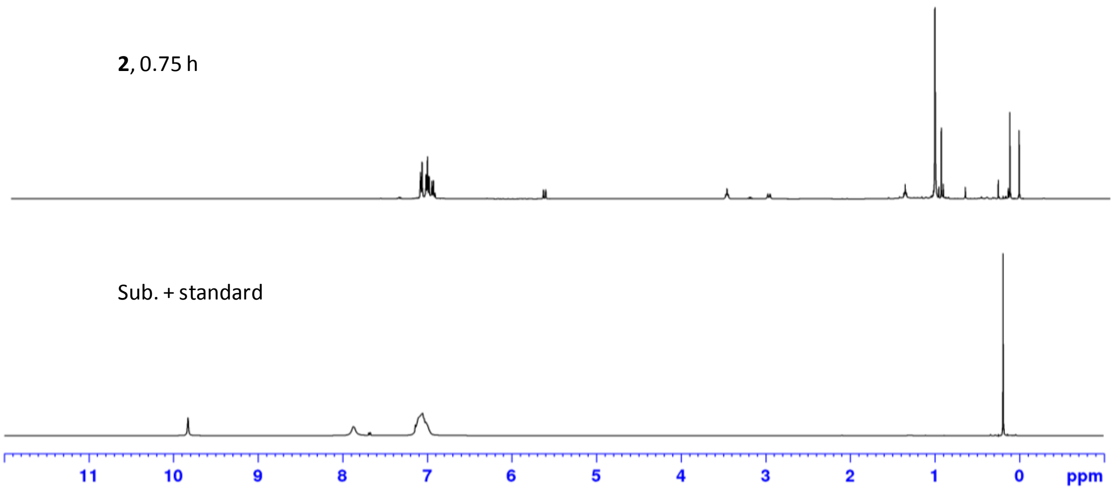

Figure S56: ^{1}H NMR spectrum for the hydroboration of benzophenone imine catalysed by **2** (10 mol% Al) in C_6D_6

Benzophenone imine catalysed by $[iBu_3Al(H)Li]_2$, 3

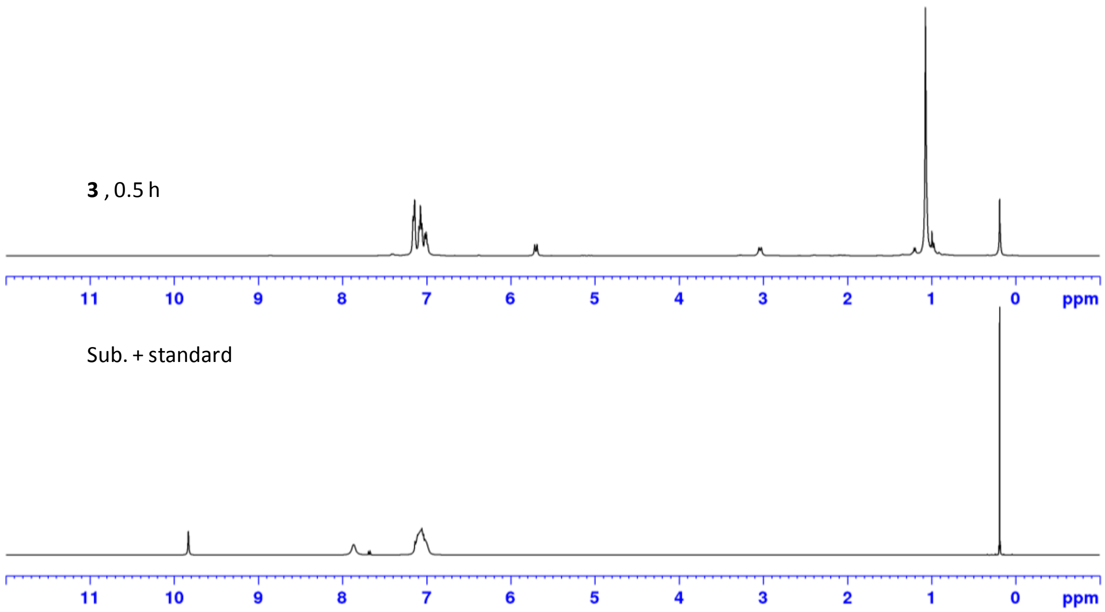

Figure S57: ^{1}H NMR spectrum for the hydroboration of benzophenone imine catalysed by **3** (10 mol% Al) in C_6D_6

Benzophenone imine catalysed by $iBu_2Al(TMP)$, 4

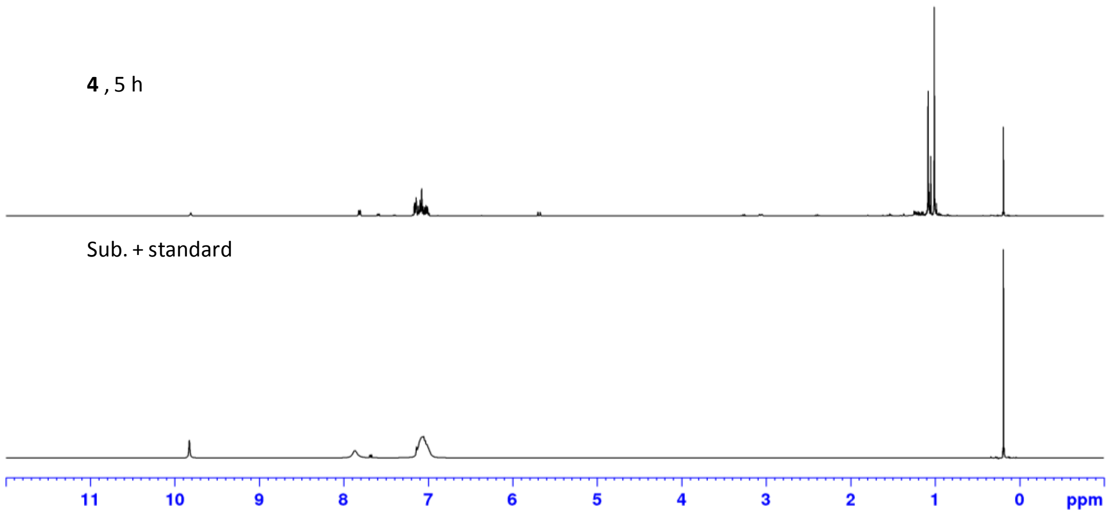

Figure S58: ^{1}H NMR spectrum for the hydroboration of benzophenone imine catalysed by **4** (10 mol% Al) in C_6D_6

Benzophenone imine catalysed by $HMDS_2AlH$, 5

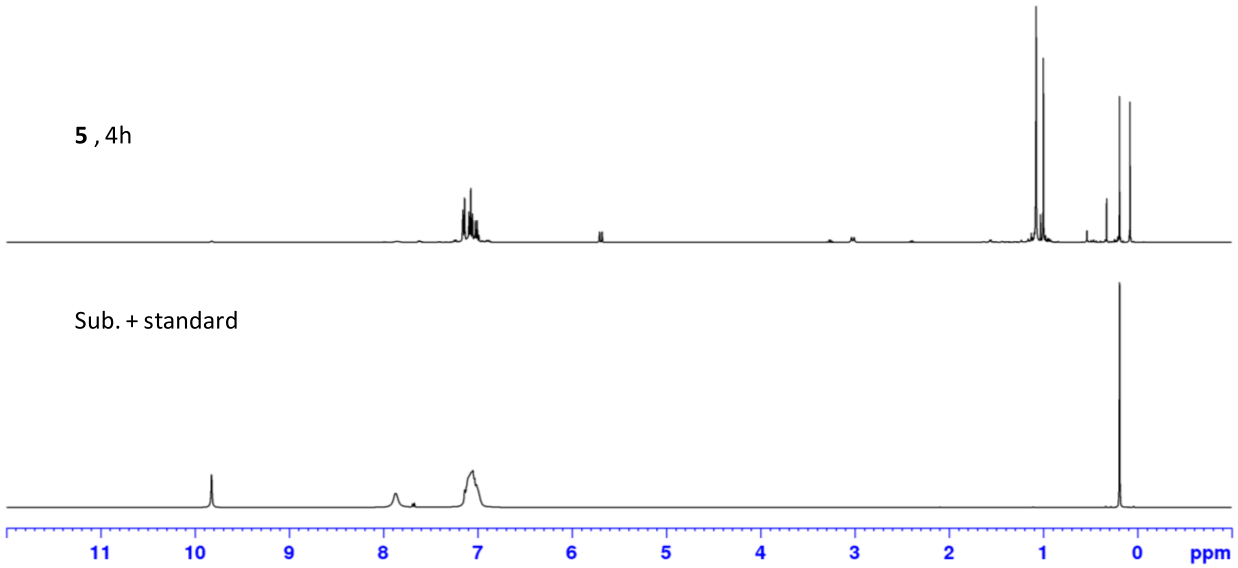

Figure S59: ^{1}H NMR spectrum for the hydroboration of benzophenone imine catalysed by **5** (10 mol% Al) in C_6D_6

Benzophenone imine catalysed by DIBAL(H), 6

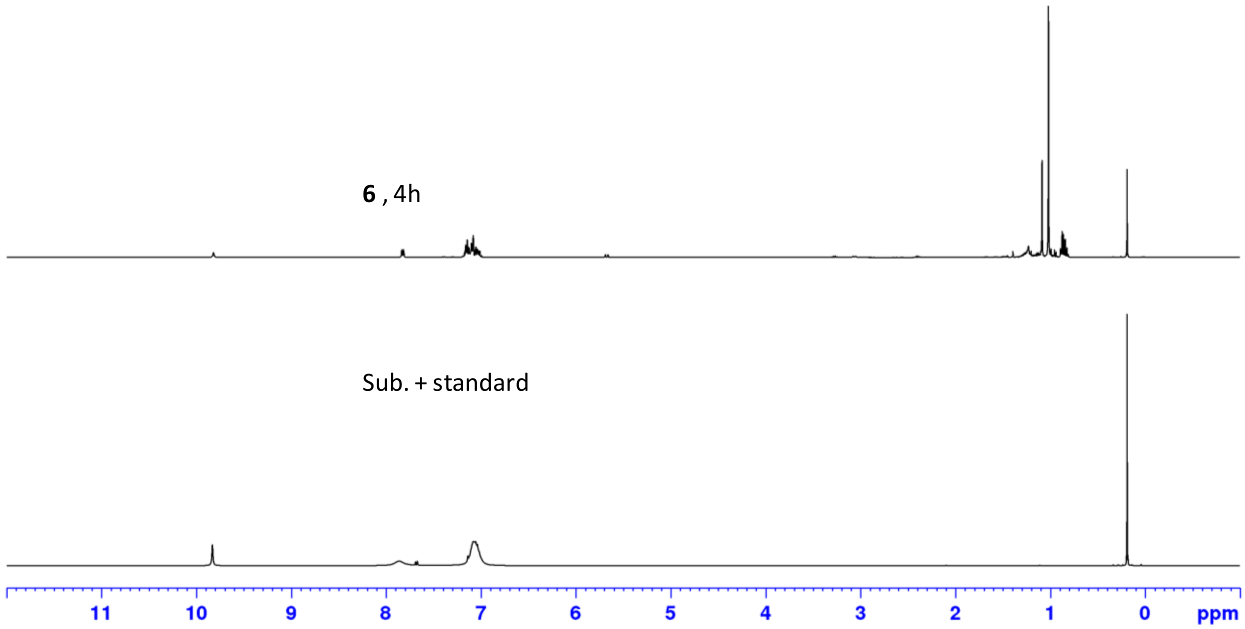

Figure S60: ^{1}H NMR spectrum for the hydroboration of benzophenone imine catalysed by **6** (10 mol% Al) in C_6D_6

Stoichiometric Reactions of Alkynes:

Phenyl acetylene + $[iBu_3Al(H)Li]_2$, **3**

In a J. Young's NMR tube $[iBu_3Al(H)Li]_2$ (0.026 g; 0.0625 mmol) was dissolved in C_6D_6 (0.5 mL) and phenyl acetylene (0.14 mL; 0.125 mmol) was added.

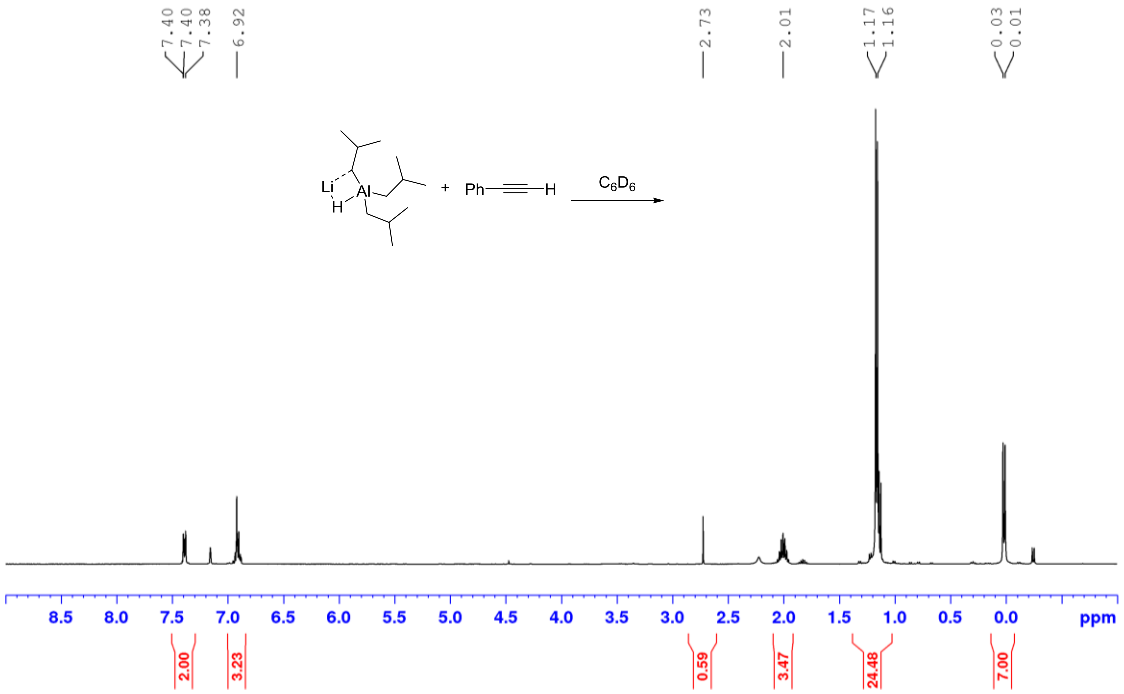

Figure S61: J Young's NMR reaction between phenyl acetylene + iBu3Al(H)Li, 3, in C6D6, showing no reaction at room temperature

Phenyl acetylene + iBu$_2$Al(TMP), **4**

In a J. Young's NMR tube iBu$_2$Al(TMP) (0.070 g; 0.25 mmol) was dissolved in C_6D_6 (0.5 mL) and phenyl acetylene added (0.030 mL; 0.25 mmol)

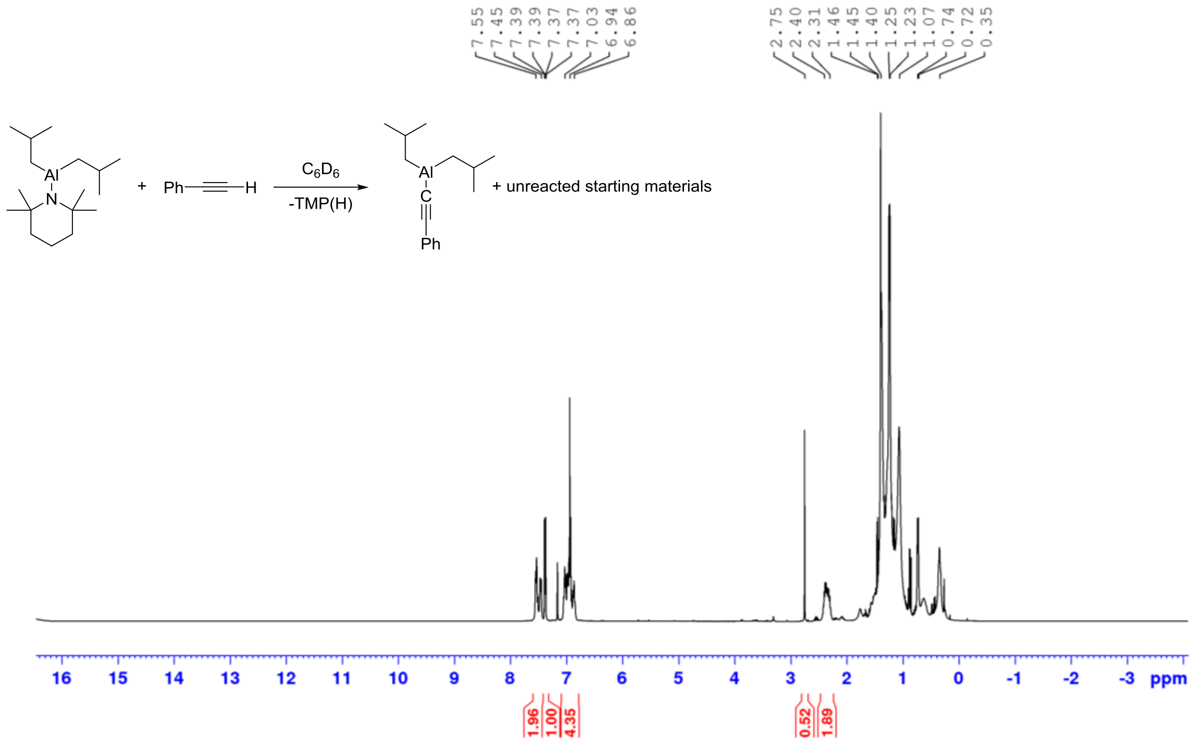

Figure S62: J Young's NMR reaction between phenyl acetylene + iBu$_2$Al(TMP), **4**, in C_6D_6, showing slow deprotonation at room temperature.

Phenyl acetylene + iBu$_2$AlH, **6**

In a J. Young's NMR tube DIBAL (0.0178 g; 0.125 mmol) was dissolved in C_6D_6 (0.5 mL) and then phenyl acetylene (0.021 mL; 0.125 mmol) was added.

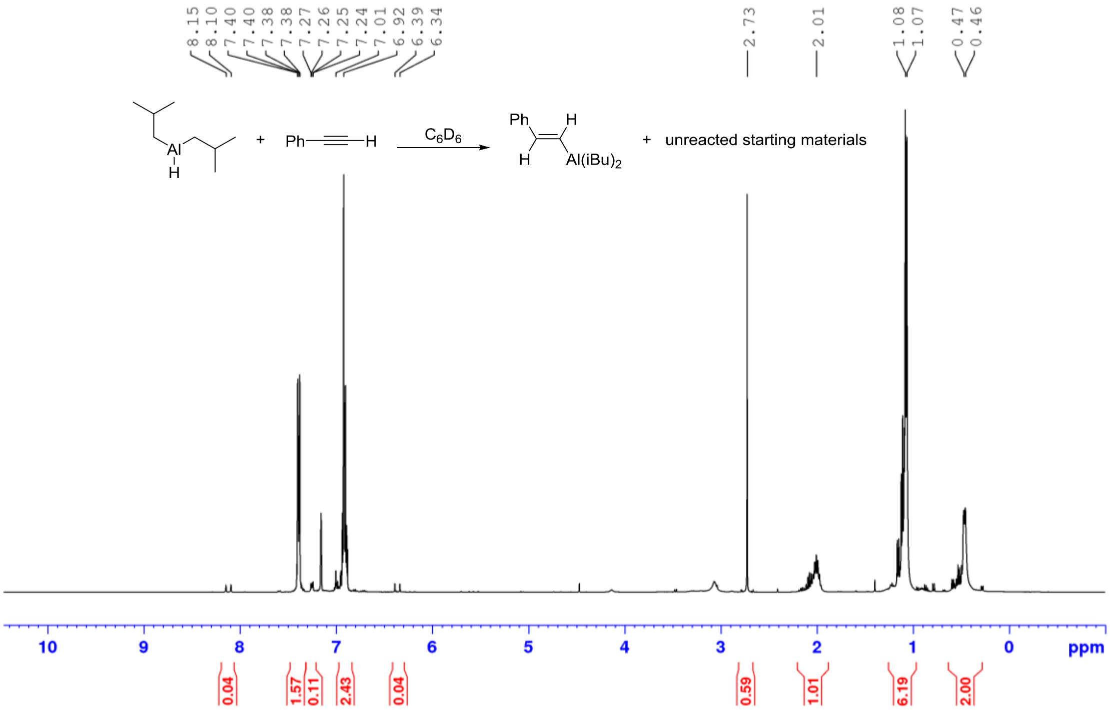

Figure S63: J Young's NMR reaction between phenyl acetylene + iBu$_2$AlH, **6**, in C_6D_6, showing slow hydroalumination at room temperature.

Hydroboration of Alkynes

Hydroboration reactions of phenyl acetylene and diphenylacetylene were performed at 0.5 mmol scale in d8-toluene (0.5 mL) at 110 °C using 10 mol% [Al] catalyst **1**, **3**, **4**, or **6**.

Phenyl acetylene catalysed by [iBu$_2$Al(TMP)(H)Li]$_2$, 1

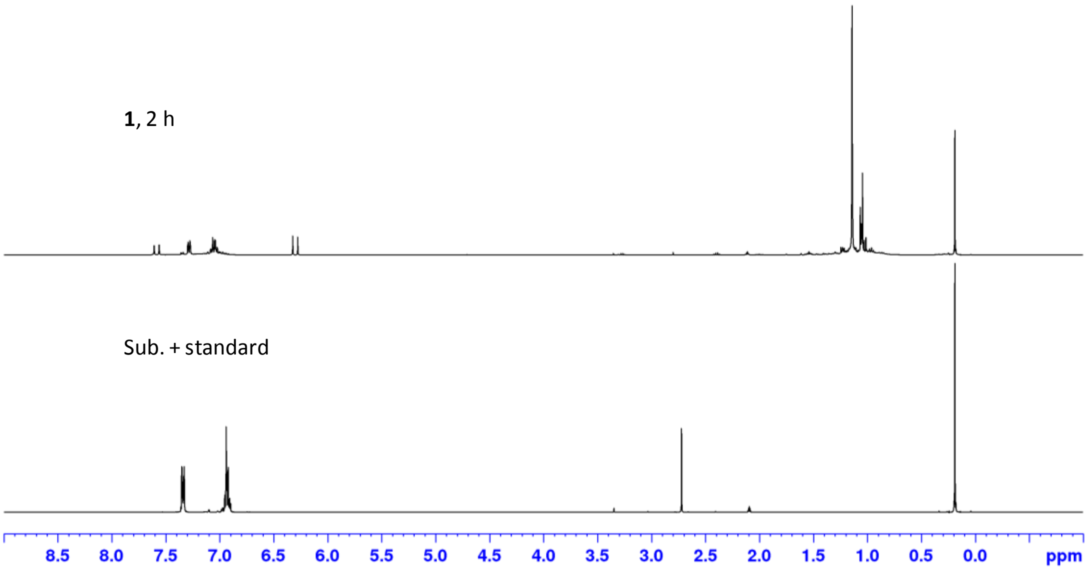

Figure S64: ^{1}H NMR spectrum for the hydroboration of phenyl acetylene catalysed by **1** (10 mol% Al) in d$_8$-toluene at 110 °C

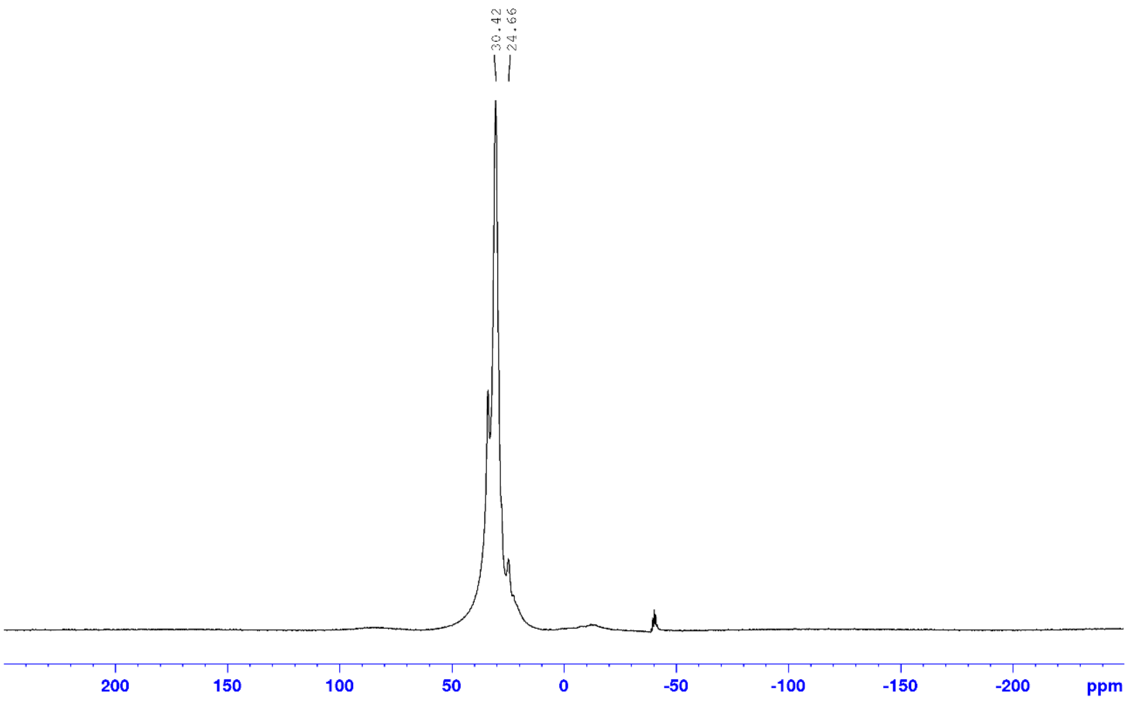

Figure S65: ^{11}B NMR spectrum for the hydroboration of phenyl acetylene catalysed by **1** (10 mol% Al) in d$_8$-toluene

Phenyl acetylene catalysed by [$iBu_3Al(H)Li]_2$, **3**

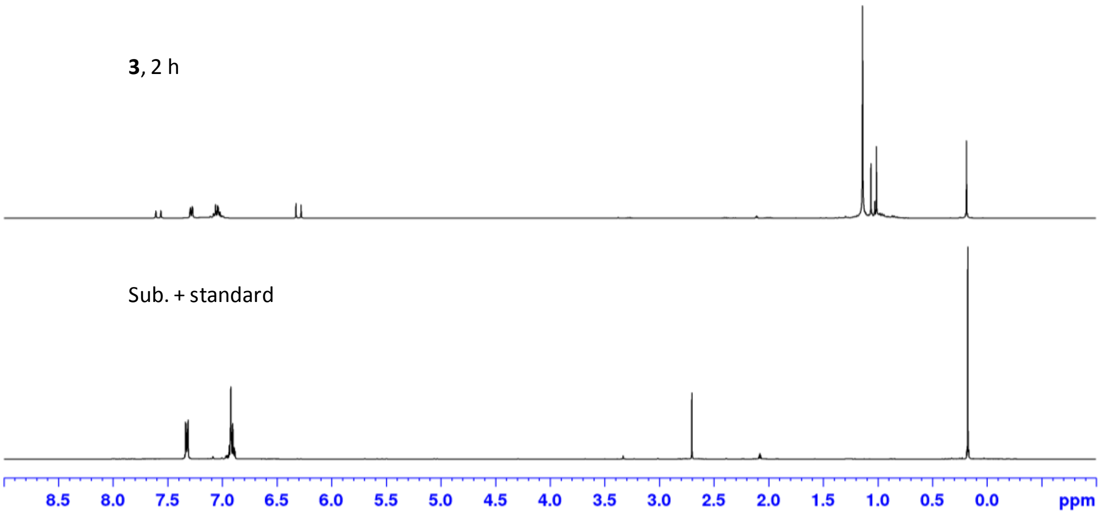

Figure S66: ^{1}H NMR spectrum for the hydroboration of phenyl acetylene catalysed by **3** (10 mol% Al) in d_8-toluene at 110 ^{o}C

Phenyl acetylene catalysed by $iBu_2Al(TMP)$, **4**

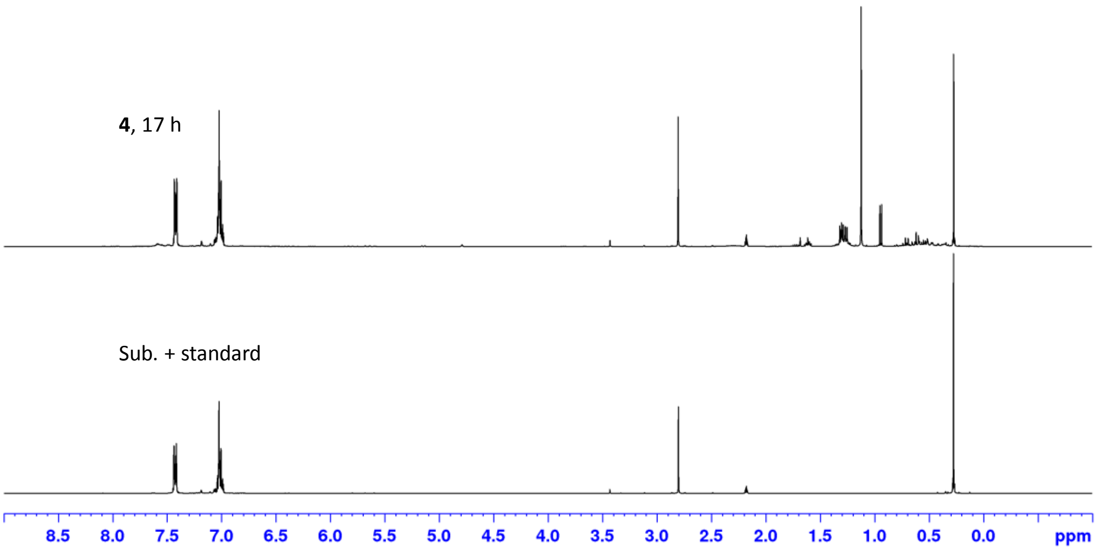

Figure S67: ^{1}H NMR spectrum for the hydroboration of phenyl acetylene catalysed by **4** (10 mol% Al) in d_8-toluene at 110 ^{o}C

Diphenyl acetylene catalysed by $[iBu_2Al(TMP)(H)Li]_2$, **1**

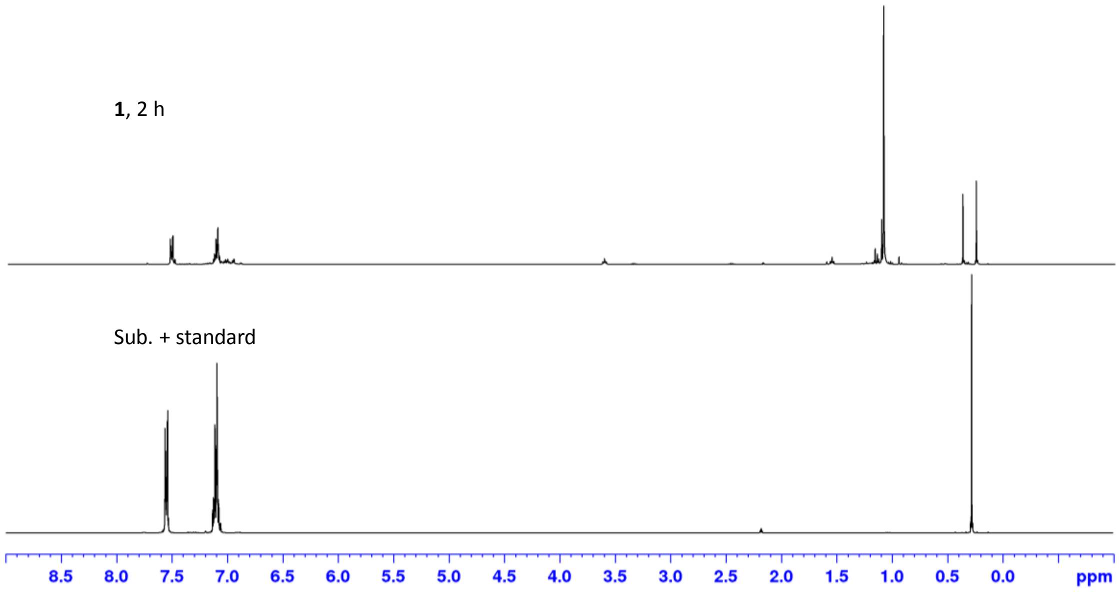

Figure S68: ^{1}H NMR spectrum for the hydroboration of diphenyl acetylene catalysed by **1** (10 mol% Al) in d_8-toluene at 110 ^{o}C

Diphenyl acetylene catalysed by $[iBu_2Al(TMP)(H)Li]_2$, **3**

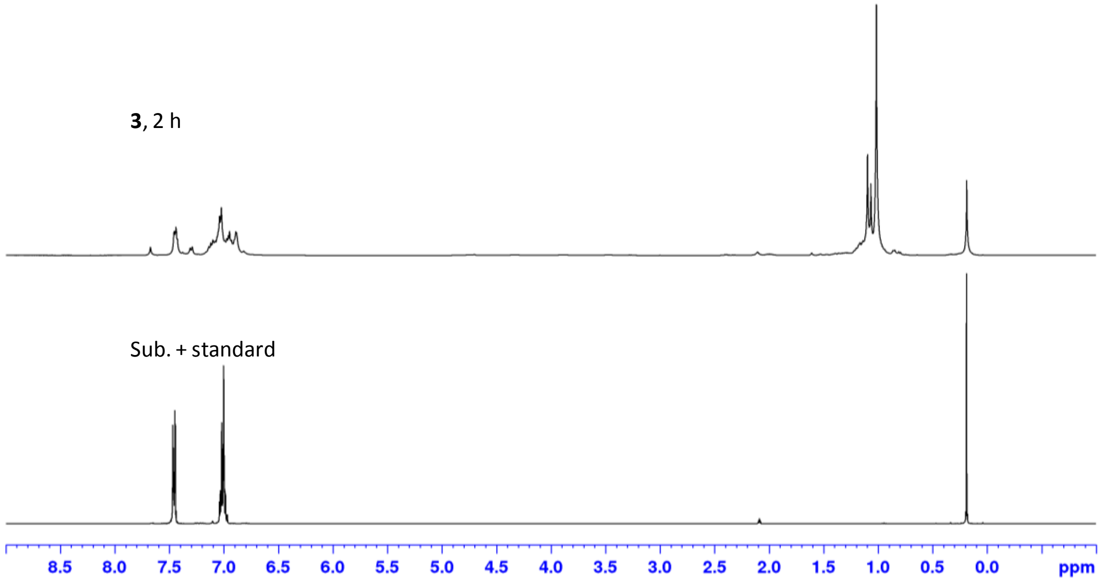

Figure S69: ^{1}H NMR spectrum for the hydroboration of diphenyl acetylene catalysed by **3** (10 mol% Al) in d_8-toluene at 110 ^{o}C

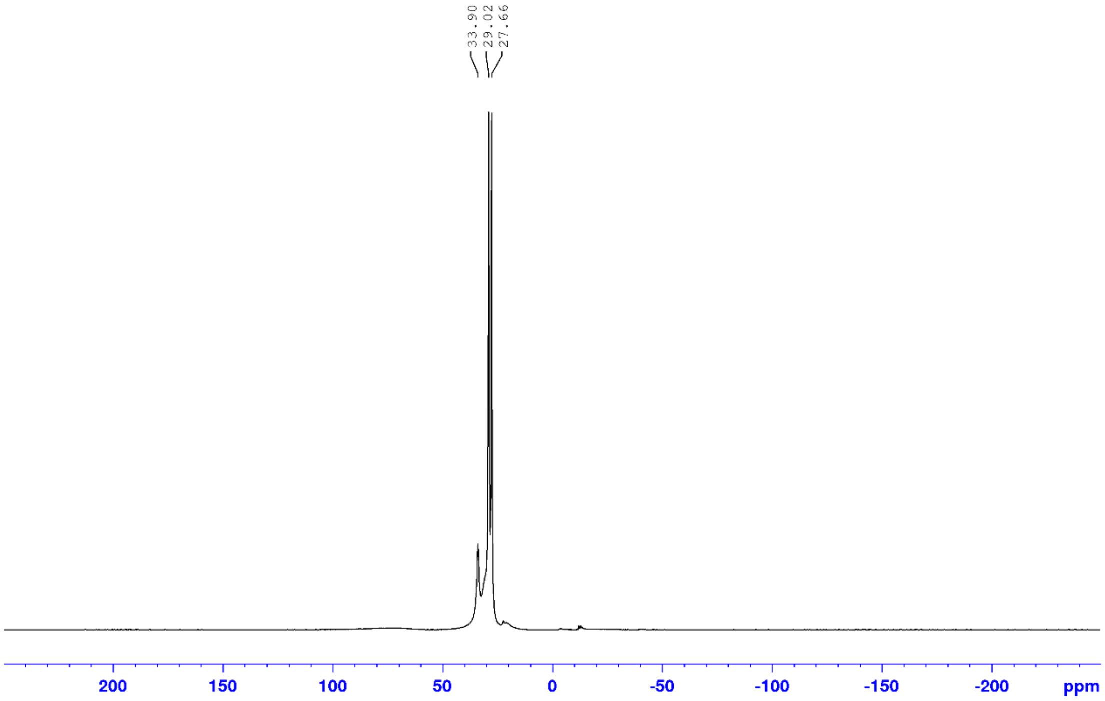

Figure S70: ^{11}B NMR spectrum for the hydroboration of diphenyl acetylene catalysed by **3** (10 mol% Al) in d_8-toluene

1-phenyl-1-propyne catalysed by $[iBu_2Al(TMP)(H)Li]_2$, 3

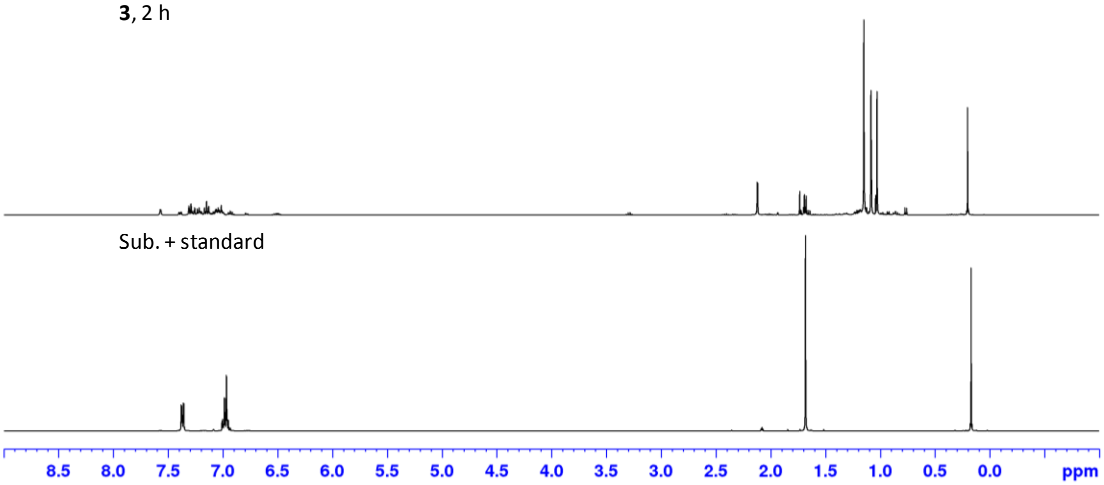

Figure S71: ^{1}H NMR spectrum for the hydroboration of diphenyl 1-phenyl-1-propyne catalysed by **3** (10 mol% Al) in d_8-toluene at 110 ^{o}C

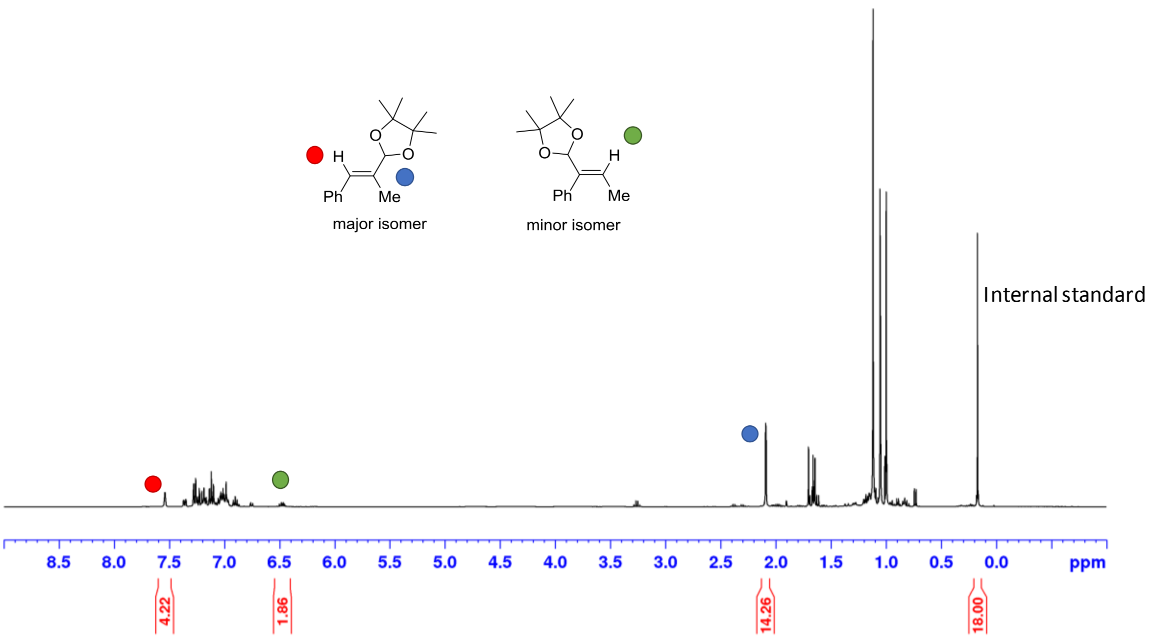

Figure S72: ^{1}H NMR spectrum for the hydroboration of diphenyl 1-phenyl-1-propyne catalysed by **3** (10 mol% Al) in d_8-toluene at 110 °C, showing ratio of major to minor product as 2.2: 1.

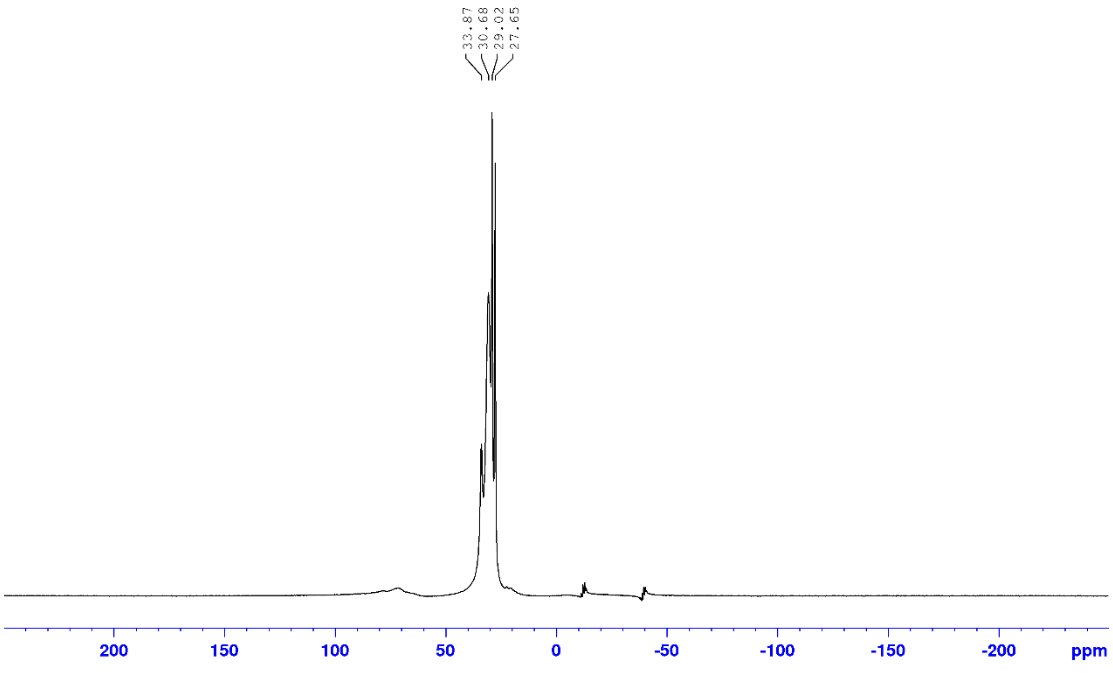

Figure S73: ^{11}B NMR spectrum for the hydroboration of 1-phenyl-1-propyne catalysed by **3** (10 mol% Al) in d_8-toluene

Table S1: Crystal structure data and refinement details for compounds **7** and **8**

**Compound**	**[(TMP)(Ph$_2$(H)CO)Al(μ-OC(H)Ph$_2$)]$_2$, 7**	**[iBu$_2$Al(μ-NCPh$_2$]$_2$, 8**
**Empirical formula**	$C_{70}H_{80}Al_2N_2O_4$	$C_{42}H_{56}Al_2N_2$
**Mol. Mass**	1067.32	642.89
**Crystal system**	Monoclinic	Triclinic
**a/ Å**	14.04490(10)	10.4347(6)
**b/ Å**	17.85020(10)	19.3559(14)
**c/Å**	23.1821(2)	21.3099(13)
**α/ °**	90	70.236(6)
**β/ °**	92.8660(10)	89.901(5)
**ϒ/ °**	90	81.826(5)
**V/ Å^3**	5804.58(7)	4004.4(5)
Z	4	4
**λ/ Å**	Cu Kα (λ = 1.54184)	Mo Kα (λ = 0.71073)
**Measured reflections**	72409	30347
**Unique reflections**	11508	15688
**R_{int}**	0.0736	0.055
**Observed rflns [I > 2σ(I)]**	10297	8801
**GooF**	1.025	0.994
**R [on *F*, obs rflns only]**	0.0458	0.0678
**ωR [on F^2, all data]**	0.1237	0.1621
**Largest diff. peak/ hole e/ Å^{-3}**	0.409/ -0.263	0.72/ -0.55

1. D. R. Armstrong, E. Crosbie, E. Hevia, R. E. Mulvey, D. L. Ramsay and S. D. Robertson, *Chem. Sci.*, 2014, **5**, 3031-3045.
2. S. Christoph and A. Reiner, *European Journal of Inorganic Chemistry*, 2013, **2013**, 3302-3306.
3. R. E. Mulvey, J. Okuda, L. E. Lemmerz, R. McLellan, N. R. Judge, A. R. Kennedy, S. A. Orr, M. Uzelac, E. Hevia and S. D. Robertson, *Chem. -Eur. J.*, 2018, **0**.
4. G. M. Sheldrick, *Acta Crystallogr.*, 2008, **A64**, 112 - 122.
5. G. M. Sheldrick, *Acta Crystallogr.*, 2015, **C71**, 3 -8
6. A. P. Dove, V. C. Gibson, E. L. Hormnirun, E. L. Marshall, J. A. Segal, A. J. P. White and D. J. Williams, *Dalton Trans.*, 2003, 3088 - 3097.
7. O. V. Dolomanov, L. J. Bourhis, R. J. Gildea, J. A. K. Howard and H. Puschmann, *J. Appl. Crystallogr.*, 2009, **42**, 339 - 341.
